# Tip growth in a strongly concentrated conformal growth model follows local geodesics

**DOI:** 10.1007/s00440-026-01494-w

**Published:** 2026-05-21

**Authors:** Frankie Higgs

**Affiliations:** Department of Mathematical Sciences, University of Bath, Claverton Down, Bath, BA2 7AY UK

**Keywords:** Random growth, Conformal mapping, Scaling limits, Harmonic measure, Martingales, 60F99, 60D05, 30C20, 60J67, 60G42

## Abstract

We analyse the *aggregate Loewner evolution* (ALE), introduced in 2018 by Sola, Turner and Viklund to generalise versions of diffusion limited aggregation (DLA) in the plane using complex analysis. They showed convergence of the ALE for certain parameters to a single growing slit. Started from a non-trivial initial configuration of *k* needles and the same parameters, we show that the small-particle scaling limit of ALE is the *Laplacian path model*, introduced by Carleson and Makarov, in which the tips grow along geodesics towards ∞. Our proof involves analysis of Loewner’s equation near its singular points, and we extend martingale methods to the backward equation, where what we have to control is non-adapted. Most conformal growth models introduce an extra regularisation factor to deal with the singularities in Loewner’s equation at the sharp tips and right-angle bases of slit particles. As an intermediate step we prove a limit result for a model with no such regularisation factor, developing methods which should prove useful in analysing other weakly-regularised models with non-trivial limits.

## Introduction

We study two models of growth in the plane, identified with $\mathbb {C}$. The aggregate Loewner evolution (ALE) model was introduced in [1] as a version of diffusion limited aggregation (DLA) which could take advantage of the natural isotropy of $\mathbb {C}$ and complex-analytic tools. Via the Riemann mapping theorem, we have a one-to-one correspondence between a certain family of conformal maps and the collection of compact simply-connected subsets of $\mathbb {C}$. By composing simple conformal maps (corresponding to “particles”) we construct a map representing more complicated clusters. The continuity of the one-to-one correspondence between maps and clusters allows us to prove limit results about the cluster by analysing the corresponding map, so a powerful set of tools from complex analysis becomes available for analysing growth models.

The Laplacian path model (LPM) was introduced in [2] as a model of tip growth, in which *k* simple curves attached to the unit disc grow towards ∞ in the Riemann sphere. The growth speeds are controlled by a certain Laplacian field, which allows the LPM to model “screening” effects, in which the growth rate of each curve is affected by how much its “view of ∞” is blocked by other curves. The strength of the influence of this field is parameterised by a single $\eta \in \mathbb {R}$.

We will prove that the ALE model of particle aggregation converges, in the limit as individual particles become small, to the LPM. This is an extension of the result of [1], that ALE started from a disc with no slits initially attached converges to an LPM with one arm, i.e. to a straight line. Our methods involve an explicit analysis of the tips of particles, where usual martingale methods are difficult to apply due to singularities in *Loewner’s equation* which describes both the ALE and LPM.

## Preliminaries

### Loewner’s equation

Loewner’s equation describes growing sets in the complex plane. The aggregate Loewner evolution (ALE) model of particle growth is defined by composing conformal maps, but we can also view it as a solution to Loewner’s equation with a certain driving function. Loewner’s equation also defines the claimed limit: the Laplacian path model (LPM).

Loewner’s equation encodes families of growing sets by measures on a cylinder. Continuity properties of the encoding allow us to show the sets are close if the corresponding measures are close.

#### Definition 2.1.1

Let $\mathbb {D}= \{ z \in \mathbb {C}: |z| < 1 \}$ be the open unit disc, and $\Delta = \mathbb {C}_{\infty } \setminus \overline{\mathbb {D}}$ the open *exterior disc*, the complement of the closed unit disc in the Riemann sphere. Let $\mathbb {T}= \partial \mathbb {D}$ denote their common boundary, the unit circle.

Given a compact, simply connected $K \subseteq \mathbb {C}$ with 0∈K (and $K \not = \{0\}$), by the Riemann mapping theorem there is a unique conformal map $f_K: \Delta \rightarrow \Delta \setminus K$ with $f_K(\infty ) = \infty$ and $f_K(z) = e^c z + O(1)$ for some $c = c(K) \in \mathbb {R}$ as $z \rightarrow \infty$. We call *c*(*K*) the *(logarithmic) capacity* of *K*.

If *K* is a strict superset of $\overline{\mathbb {D}}$, then c(K)>0.^1^ Another useful property of logarithmic capacity is *additivity*: if $f_K$ can be written $f_{K_1} \circ f_{K_2}$, then $c(K) = c(K_1) + c(K_2)$.

We can encode certain families of growing compact sets $(K_t)_{t \ge 0}$ using *Loewner’s equation*.

#### Definition 2.1.2

Let $\xi : [0, T] \rightarrow \mathbb {R}$ be a càdlàg function^2^. Then there is a unique solution $\varphi : [0,T]\times \Delta \rightarrow \Delta$ to Loewner’s equation,

2.1 $$\begin{aligned} \varphi _0(z) = z, \quad \frac{\partial }{\partial t} \varphi _t(z) = \varphi _t'(z) z \frac{z + e^{i\xi _t}}{z - e^{i\xi _t}}, \quad z \in \Delta , \end{aligned}$$

corresponding to a growing cluster $K_t:= \mathbb {C}_\infty \setminus \varphi _t(\Delta )$ which is parameterised by capacity, i.e. $c(K_t) = t$. The *driving function* $\xi _t$ encodes where the growth at time *t* is located on the cluster boundary.

For the existence and uniqueness of the solution to (2.1), see [3, Theorem 3.4] (note that every càdlàg function is piecewise continuous, meeting the conditions in [3]).

We can generalise (2.1) to encode clusters growing at arbitrary parts of their boundary, specified by a measure:

#### Definition 2.1.3

Given a family of probability measures on $\mathbb {T}$, $(\mu _t)_{t \ge 0}$, subject to measurability conditions on $t \mapsto \mu _t$ there is a unique solution to Loewner’s equation

2.2 $$\begin{aligned} \varphi _0(z) = z, \quad \frac{\partial }{\partial t} \varphi _t(z) = \varphi _t'(z) \int _\mathbb {T}z \frac{z + e^{i\theta }}{z - e^{i\theta }}\,\textrm{d}\mu _t(\theta ), \quad z \in \Delta . \end{aligned}$$

It is a simple generalisation to start Loewner’s equation from a non-trivial initial condition $\varphi _0$, provided $\varphi _0$ is a conformal map of the form $f_K$ for some *K* as above.

### Particle aggregation models

We construct the aggregate Loewner evolution (ALE) cluster by composing conformal maps.

#### Definition 2.2.1

Let $\textbf{c}> 0$, and define $d = d(\textbf{c}) > 0$ to be the unique length such that $\overline{\mathbb {D}}\cup (1,1+d]$ has capacity $\textbf{c}$.

We can determine explicitly that *d* satisfies $(d+2)^2/(d+1) = 4e^{\textbf{c}}$, and asymptotically $d \sim 2\textbf{c}^{1/2}$ as $\textbf{c}\rightarrow 0$.

Let $f^{\textbf{c}}$ be the unique conformal map $\Delta \rightarrow \Delta \setminus (1,1+d]$ with $f^{\textbf{c}}(z) \sim e^{\textbf{c}}z$ as $z \rightarrow \infty$.

Given an angle $\theta \in \mathbb {R}$, we can define the rotated map

$$\begin{aligned}&f^{\theta ,\textbf{c}} : \Delta \rightarrow \Delta \setminus e^{i\theta }(1,1+d],\\&f^{\theta ,\textbf{c}}(z) = e^{i\theta } f^{\textbf{c}}(e^{-i\theta }z). \end{aligned}$$

Given a sequence of angles $(\theta _n)_{n \ge 1}$ and of capacities $(c_n)_{n \ge 1}$, let $f_j = f^{\theta _j,c_j}$ and

$$\begin{aligned} \Phi _n := f_1 \circ f_2 \circ \dots \circ f_n. \end{aligned}$$

Define the *n*th cluster $K_n$ as the complement of $\Phi _n(\Delta )$, so

$$ \Phi _n : \Delta \rightarrow \mathbb {C}_{\infty } \setminus K_n. $$

Note that the total capacity is $c(K_n) = \sum _{k=1}^{n} c_k$.

Any cluster constructed this way can be encoded by Loewner’s equation. For t≥0 let $n_t = \inf \{ n \ge 1: \sum _{k=1}^{n} c_k > t \}$ (if $c_n = \textbf{c}$ for all *n*, then $n_t = \left\lfloor t/\textbf{c} \right\rfloor +1$). Let $\xi _t:= \theta _{n_t}$, and let $(\varphi _t)_{t \ge 0}$ be the solution to (2.1) with driving measure $(\xi _t)_{t \ge 0}$. Then for all *n*, $\varphi _{c(K_n)} = \Phi _n$.

### Aggregate Loewner evolution

If $(\theta _n)_{n \ge 1}$ and $(c_n)_{n \ge 1}$ are stochastic processes, then $(K_n)_{n \ge 1}$ as constructed in Section 2.2 is a stochastic process in the space of compact subsets of $\mathbb {C}$. The aggregate Loewner evolution ALE($\alpha ,\eta ,\sigma$), introduced in [1], is one such process.

**Fig. 1:**
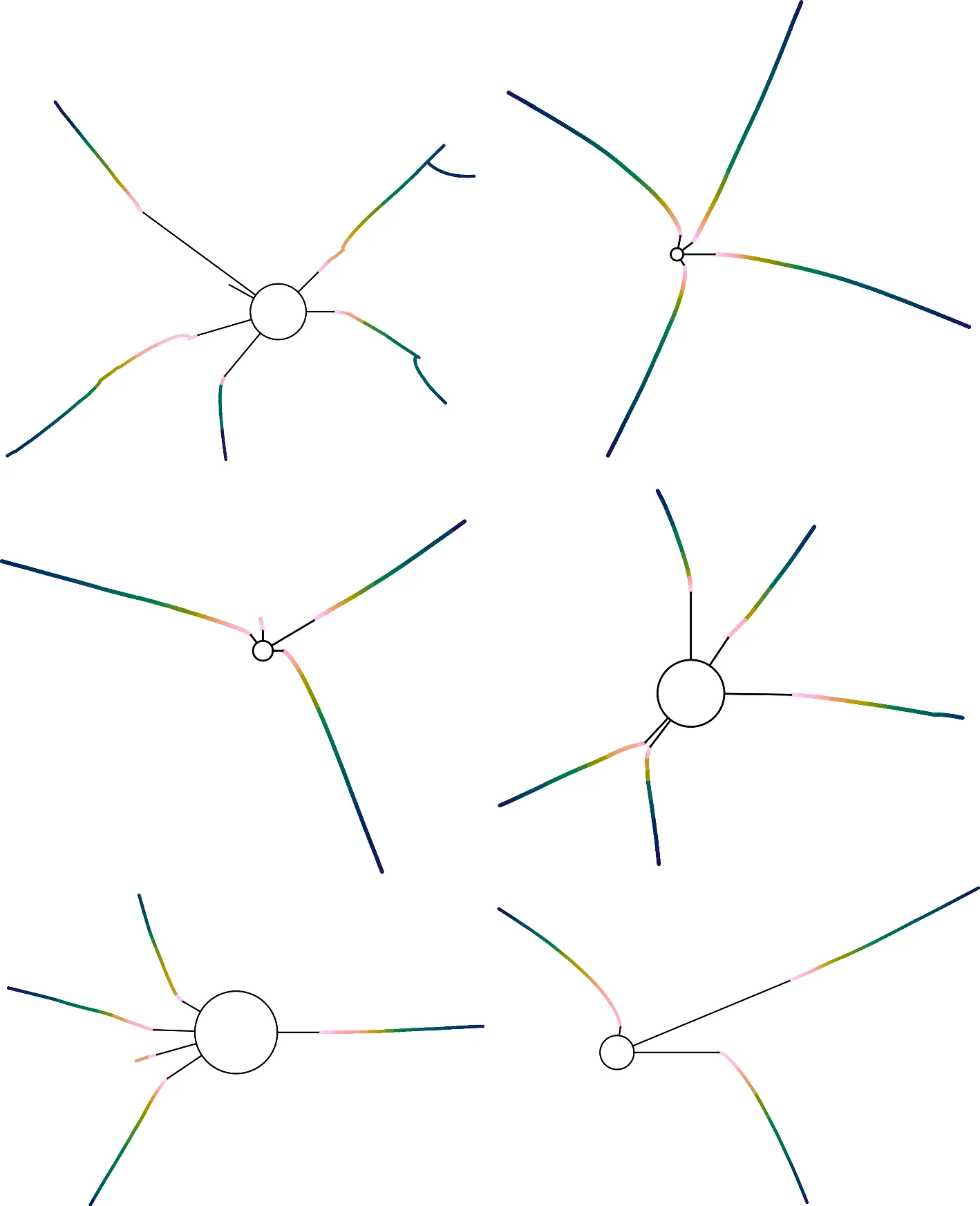
Simulations of the aggregate Loewner evolution started from a non-trivial configuration of arms. The initial cluster is drawn with thin black lines, and the attached particles are coloured by arrival time (the lighter particles arrived earlier). In the top-left diagram σ is large enough that some particles are not attached at the tips of the existing slits and we see “branching” behaviour which does not occur in the limiting regime. In the three diagrams on the left one slit seems to grow much slower than the rest, agreeing with the observation in [2] that there is competition between the arms in the Laplacian path model, with only a certain number (depending on η) likely to survive (i.e. growth to a length proportional to the diameter of the entire cluster) as $T \rightarrow \infty$. The code used for the simulations is available at https://github.com/frankiehiggs/ALE-from-slits

#### Definition 2.3.1

We define the ALE inductively: for n≥0 we choose $\theta _{n+1}$, conditional on $\theta _1, \dots , \theta _n$, according to the probability density function

$$\begin{aligned} h_{n+1}(\theta ) = \frac{1}{Z_n} \left| \Phi _n'\left( e^{\sigma + i\theta } \right) \right| ^{-\eta },\, \theta \in (-\pi , \pi ], \end{aligned}$$

where $Z_n:= \int _{\mathbb {T}} | \Phi _n'(e^{\sigma + i\theta })|^{-\eta }\,\textrm{d}\theta$ is a normalising factor.

The particle capacities can also be modified. For n≥0 let

$$ c_{n+1} = \textbf{c}| \Phi _n'(e^{\sigma + i\theta _{n+1}})|^{-\alpha }. $$

Several simulations of the ALE in the setting of Theorem 2.1 are shown in Figure 1.

#### Remark 1

We have introduced three parameters: $\alpha \in \mathbb {R}$, $\eta \in \mathbb {R}$, and σ>0. In this paper we will work with α=0, which means that all particles have the same capacity. After the statement of Theorem 2.1 we will discuss the effect of changing α.

If σ=0 then the poles and zeroes of $\Phi _n'$ on $\partial \Delta = \mathbb {T}$ mean that the measure $h_{n+1}$ would not necessarily be well-defined. We will take σ>0 but with $\sigma \rightarrow 0$ as $\textbf{c}\rightarrow 0$.

The other parameter, η, controls the influence of *harmonic measure*. If η=0 then $(\theta _n)_{n\ge 1}$ is a sequence of independent random variables uniformly distributed on $(-\pi ,\pi ]$, corresponding to the Hastings–Levitov model [4].

We can view $|(\Phi _{n}^{-1})'(z)|$ as the “density of harmonic measure” at $z \in \partial K_n$, and so when η>0, $h_{n+1}$ is concentrated on areas of high harmonic measure, and when η<0 it is concentrated on areas of low harmonic measure.

#### Remark 2

In [5], a phase transition was proved for negative η: for η<-2 and sufficiently small σ the ALE cluster converges to a *Schramm–Loewner evolution*.

The authors of [1] showed for η>1 that the scaling limit of an ALE with initial cluster $\overline{\mathbb {D}}$ is a single straight slit. In fact they showed a stronger degeneracy result: with probability tending to 1 as $\textbf{c}\rightarrow 0$, each particle attaches near the tip of its immediate predecessor. Theorem 2.1 extends this result to the case where $K_0$ is the union of $\overline{\mathbb {D}}$ with more than one slit.

A series of videos illustrating the effects of η and α is available at this link.^3^

### Laplacian path model

The Laplacian path model (LPM) was defined in 2002 by Carleson and Makarov [2] to generalise several models of needle-like growth in mathematical physics. We define the Laplacian path model as a growing set in the complex plane using Loewner’s equation (2.2): the LPM is encoded by an initial configuration of *k* arms $K_0$ (which uniquely determines $\varphi _0$), and a driving measure $(\mu _t)_{t \ge 0}$.

#### Definition 2.4.1

Let $K_0$ be the union of rays of the form $e^{i\theta _j} (1, 1+d_j]$, where the $\theta _j$ are distinct angles in $(-\pi ,\pi ]$ and $d_j > 0$ for j=1,⋯,k. Let $\Delta _0 = \Delta \setminus K_0$, and $\Phi _0^{\textrm{LPM}}$ the unique conformal map $\Phi _0^{\textrm{LPM}}: \Delta \rightarrow \Delta _0$ satisfying $\Phi _0^{\textrm{LPM}}(z) = e^{c_0} z + O(1)$ as $z \rightarrow \infty$ for some positive $c_0$.

We define the LPM cluster with parameter η, which has *k* growing slits whose tips at time t>0 are at $a^j_t \in \Delta \setminus K_0$, via Loewner’s equation with initial condition $\varphi _0(z) = \Phi _0^{\textrm{LPM}}(z)$. We have the driving measure

2.3 $$\begin{aligned} \mu ^{\textrm{LPM}}_t = \sum _{j=1}^{k} p^j_t \delta _{\phi ^j_t}, \end{aligned}$$

where $e^{i\phi ^j_t}$ is the preimage of $a^j_t$ under $\Phi _t^{\textrm{LPM}}$, and

2.4 $$\begin{aligned} p^j_t := \frac{|(\Phi _t^{\textrm{LPM}})''(e^{i\phi ^j_t})|^{-\eta }}{Z_t}, \end{aligned}$$

and

2.5 $$\begin{aligned} Z_t := \sum _{j=1}^{k} |(\Phi _t^{\textrm{LPM}})''(e^{i\phi ^j_t})|^{-\eta }. \end{aligned}$$

#### Remark 3

We can think of the growth of each slit in the geodesic LPM at time *t* as being in the direction of the hyperbolic geodesic from $a^j_t$ to ∞ in $\Delta \setminus K_t$, at a speed proportional to $|(\Phi _t^{\textrm{LPM}})''(e^{i\phi ^j_t})|^{-(\eta -1)}$.

#### Remark 4

The weight given to the *j*th slit by (2.4) depends on the second derivative of $\Phi _t^{\textrm{LPM}}$, while the probability of attaching near a given point in the ALE model depends on the first derivative of $\Phi _t^{\textrm{ALE}}$.

#### Remark 5

In [2], Carleson and Makarov obtained the definition involving the second derivative by considering growth speeds proportional to the Laplacian field $\nabla G_t$, where $G_t$ is the Green’s function of $\Delta \setminus K_t$, given by $G_t(z) = \log |(\Phi _t^{\textrm{LPM}})'(z) |$.

We could also derive $\mu _t^{\textrm{LPM}}$ by removing the regularisation from the ALE attachment measure. For σ>0, let $\mu _t^\sigma$ be the regularised ALE measure on $\mathbb {T}$ corresponding to the LPM cluster at time *t*, i.e. the measure with density $h^\sigma (\theta ) \propto (\Phi _t^{\textrm{LPM}})'(e^{\sigma + i\theta })|^{-\eta }$. If η>1 then as $\sigma \rightarrow 0$ the density becomes concentrated around the zeroes of $(\Phi _t^{\textrm{LPM}})'$ at $\phi _t^j$ for j=1,⋯,k, and $|(\Phi _t^{\textrm{LPM}})'(e^{\sigma + i\phi ^j_t})| \sim \sigma |(\Phi _t^{\textrm{LPM}})''(e^{i\phi _t^j})|$. Hence $\mu _t^\sigma \rightarrow \mu _t^{\textrm{LPM}}$ as $\sigma \rightarrow 0$ for a fixed time *t*.

#### Remark 6

The η in Definition 2.4.1 corresponds to η-1 in the definition given in [2]; we have shifted it to match the η used in the definition of the ALE in [1].

#### Remark 7

For any configuration of *k* slits, $K_0$ is a “gearlike domain” as defined in [6], and so Schwarz–Christoffel methods can be used to compute an expression for $\Phi _0^{\textrm{LPM}}$. To compute the expression, the preimages of all the tips and bases of the slits must be determined, which can be done numerically using methods from [6]. We used this to compute the maps for arbitrary slit configurations in our simulation of the ALE shown in Figure 1.

Almost all known results about the Laplacian path model appear in [2] and [7]. In [2], Carleson and Makarov defined both the geodesic LPM above and the *needle LPM*, in which the cluster is a collection of straight slits which grow at speed proportional to $|(\Phi ^{\textrm{LPM}}_t)''(e^{i\phi ^j_t})|^{-(\eta -1)}$.

Selander examined in his PhD thesis [7] a version of the geodesic LPM in which the weights $p^j_t$ are fixed constants, and the needle LPM with growth speeds proportional to harmonic measure at the tips, which corresponds to η=3/2. Each of these can be viewed as a simplification of a “non-branching DLA”.

For the needle LPM with η=3/2, Selander proved results about the stability of stationary solutions: slit configurations in which the ratio of lengths remains constant for all t≥0. For his modified geodesic model, he proved convergence as $t \rightarrow \infty$ to a stationary configuration determined by the weights, when started from any initial configuration.

For a simplified “chordal” geodesic LPM, in which finitely many curves grow from the tip of an infinite half-line, Carleson and Makarov proved a stability result: starting the process from a two-arm configuration, if η<2 then both arms always survive, while there are configurations in which only one arm survives if η>2. We will use some of the techniques they developed for the chordal case when we analyse the ALE and non-chordal LPM.

For the unsimplified geodesic LPM (i.e. the model defined by (2.3)), Carleson and Makarov also proved a stability result. Theorem 5 of [2] states that if $1< \eta < \eta _c$ for a critical value $\eta _c = \frac{18}{3 + 4\log 2} \approx 3.11815$ then the symmetric three-armed cluster is a *stable* stationary solution, i.e. the driving measure for an LPM started from an initial condition which is *close* to three symmetric arms in a certain sense converges, as $t \rightarrow \infty$, to a symmetric driving measure $\frac{1}{3}(\delta _{\theta } + \delta _{\theta +2\pi /3} + \delta _{\theta +4\pi /3})$. Moreover, it is unstable for $\eta > \eta _c$, in that small perturbations may lead to convergence to a different stationary solution.

They also proved limiting results for the simpler “needle” LPM, and made a number of conjectures about the geodesic LPM.

Although little progress has been made since 2002 on analysing the geodesic LPM, various modifications have been studied and applied. In [8], Gubiec and Szymczak apply a similar construction to model finger growth in the half-plane. We hope that relating the LPM to the more active area of conformal growth models will revive interest in the model and many of the open problems stated in [2].

The LPM and similar models have also been used to model the development of cracks in materials and formation of systems of rivers and streams. In particular, the chordal geodesic LPM with two needles at the tip of a half-line can be used to model the bifurcation of a stream. Carleson and Makarov proved that the angle between the two resulting streams in the chordal LPM must be 2π/5 (also predicted by other authors using conformal mappings [9, 10]), which agrees with the average angle of $72^\circ$ measured in a Florida stream system [11, 12].

### Main result

Our main theorem concerns convergence of the ALE as $\textbf{c}\rightarrow 0$ to the Laplacian path model cluster started from the same initial conditions.

#### Theorem 2.1

For a fixed T>0, let $(\Phi ^{\textrm{ALE}}_t)_{t\in [0,T]}$ be the ALE($\alpha ,\eta , \sigma$) map started from the initial cluster $K_0 \cup \overline{\mathbb {D}}$, where $K_0 = \bigcup _{j=1}^{k} e^{i\phi ^j_0}(1, 1+d_j]$ for $d_j > 0$ and distinct $\phi ^j_0 \in [0,2\pi )$. Let $\mu ^{\textrm{ALE}}_t$ be the driving measure for $\Phi ^{\textrm{ALE}}$, i.e. $\mu ^{\textrm{ALE}}_t = \delta _{\theta _{\left\lfloor t/\textbf{c} \right\rfloor +1}}$. Let the parameters be α=0, η>1 and $\sigma = \textbf{c}^{\gamma }$ for $\gamma = \max \left( \frac{2(\eta +2)}{\eta -1}, 8\right)$ (see the remark after Theorem 4.1 for an explanation of each term).

Let $(\Phi ^{\textrm{LPM}}_t)_{t \in [0,T]}$ be the map for the LPM started from the same initial conditions with the same parameter η, and let $\mu ^{\textrm{LPM}}_t$ be the driving measure $\mu ^{\textrm{LPM}}_t = \sum _j \bar{p}^j_t \delta _{\bar{\phi }^j_t}$.

Then

as $\textbf{c}\rightarrow 0$, $\mu ^{\textrm{ALE}}_t \otimes m_{[0,T]}$ converges in distribution to $\mu ^{\textrm{LPM}}_t \otimes m_{[0,T]}$ as random elements of the space of measures $S = \mathbb {T}\times [0,T]$, where $m_{[0,T]}$ is normalised Lebesgue measure on [0, *T*].

In particular this means if $K^{\textrm{ALE}}_t$ and $K^{\textrm{LPM}}_t$ are the respective clusters at time *t*, we have $K^{\textrm{ALE}}_T \rightarrow K^{\textrm{LPM}}_T$ weakly as a random element of the space of compact subsets of $\mathbb {C}$ (equipped with the Carathéodory topology).

#### Remark 8

In the slit convergence result of [1], a different value of γ is used, which is $\Theta ((\eta -1)^{-2})$ as $\eta \downarrow 1$, while the γ given above is $\Theta ((\eta -1)^{-1})$. The slit convergence result of [1] starts with a single slit of capacity $\textbf{c}$, while ours begin at a macroscopic size, so it is not surprising that they require a much smaller σ for their attachment measure to detect the tip of that small initial slit.

#### Remark 9

Convergence of $K_n \rightarrow K$ in the Carathéodory topology is equivalent to convergence of the corresponding maps $f_{K_n} \rightarrow f_K$ on compact subsets of Δ [13, Chapter 3.6].

#### Remark 10

In [1, Corollary 10] it is shown that if α>0, the ALE started from the trivial initial cluster $\overline{\mathbb {D}}$ still converges to the union of $\overline{\mathbb {D}}$ with a single slit. We conjecture that a similar modification of our Theorem 2.1 should also be true.

#### Conjecture 2.2

Let *T*, $K_0$, η and σ all be as in Theorem 2.1 and suppose $\alpha \ge 0$. Run the ALE$(\alpha , \eta , \sigma )$ process until the capacity reaches *T*, the ALE cluster converges as $\textbf{c}\rightarrow 0$ to the Laplacian path model with parameter $\alpha + \eta$.

We expect that proving Conjecture 2.2 would be similar to the α=0 case. The computations in Section 4 would become messier and the martingale arguments in Section 5.2 become a bit more difficult but fundamentally the same arguments should apply.

To show the convergence of the ALE model to the LPM, we introduce two intermediate models which we will call the auxiliary ALE, and the multinomial model.

The auxiliary ALE attaches particles exactly at the tips of its *k* slits, essentially replacing the ALE attachment measure, which is supported on $\mathbb {T}$ at each step, with a finite-dimensional process supported on *k* atoms like the LPM.

We will then introduce the multinomial model to replace the regularised derivative in the ALE with the second derivative *on* the boundary. This is quite simple. The most difficult and involved step is showing that the multinomial model, which is a discrete-time random growth model, converges to the continuous-time deterministic LPM. We essentially prove a strong law of large numbers at each tip: if we have attached a large number of slits by time *t* and the probability of attaching at slit *j* is *p*, then this is similar to a solution to Loewner’s equation at time *t* with a driving function that always gives weight *p* to the tip of slit *j*. It is complicated by the fact that the probabilities and weights both change over time, as do the preimages of the tips. Analysing the fluctuation of the weights at each tip from the Laplacian path model’s (2.4) is the most technical part of Section 5.2, involving controlling strong feedback in the dynamics. Another important technical detail is that Loewner’s equation is singular at the tips.

## Analytic tools

### Remark 11

Frequently throughout the paper, we will use a constant *A* which changes from line to line, having the properties that 0<A<∞ and that *A* depends only on the parameters which we are holding fixed, such as *T*, $K_0$ and η in Theorem 2.1.

We will occasionally note explicitly on which parameters *A* depends, but it is always independent of the parameter $\textbf{c}$ which we allow to tend to 0, and of the number of particles.

The choice of a slit as our particle shape is very convenient because the map and its derivative have explicit expressions:

### Proposition 3.1

Let $\textbf{c}> 0$ and $f = f^{\textbf{c}}: \Delta \rightarrow \Delta \setminus (1,1+d]$ as above. Let $\beta = \beta _\textbf{c}$ be the unique angle in (0,π) such that $f(e^{i\beta }) = 1$. Then $\beta \sim 2\textbf{c}^{1/2}$ for small $\textbf{c}$, and for $w \in \Delta$,

3.1 $$\begin{aligned} f(z) = \frac{e^{\textbf{c}}}{2w}(w+1) \left( w+1 + \sqrt{w^2 + 2(1-2e^{-\textbf{c}})w + 1}\right) - 1 \end{aligned}$$

and

3.2 $$\begin{aligned} f'(w) = \frac{f(w)}{w} \frac{w-1}{(w-e^{i\beta })^{1/2}(w-e^{-i\beta })^{1/2}}. \end{aligned}$$

### Proof

See [1, Equation (8) and Lemma 4]. □

We can find useful bounds on $|f^{\prime }\left(w\right)|$ using (3.2):

### Corollary 3.2

There are universal constants $A_1, A_2 > 0$ such that for all $\textbf{c}< 1$, for $w \in \Delta$, if $|w - e^{ i\beta }| \le \frac{3}{4}\beta$, then

$$\begin{aligned} A_1 \frac{\beta ^{1/2}}{|w - e^{i\beta }|^{1/2}} \le |f'(w)| \le A_2 \frac{\beta ^{1/2}}{|w - e^{i\beta }|^{1/2}}, \end{aligned}$$

and similarly if $|w - e^{-i\beta }| \le \frac{3}{4}\beta$.

Moreover, there is a third constant $A_3$ such that if $\min \{ | w - e^{i\beta } |, | w - e^{-i\beta } | \} > \frac{3}{4}\beta$, then

$$ |f'(w)| \le A_3. $$

Near $e^{\pm i \beta }$, *f* has a non-linear effect on distances, quantified in [5, Lemma 12]:

### Lemma 3.3

For $w \in \Delta$, for all $\textbf{c}< 1$, if $| w - e^{i\beta } | \le \beta / 2$, then

$$\begin{aligned} \begin{aligned} |f(w) - 1| =&2(e^{\textbf{c}} - 1)^{1/4} |w - e^{i\beta }|^{1/2}\\&\times \left( 1 + O\left[ \frac{|w - e^{i\beta }|}{\textbf{c}^{1/2}} \vee \textbf{c}^{1/4} |w - e^{i\beta }|^{1/2} \right] \right) . \end{aligned} \end{aligned}$$

One useful tool for analysing Loewner’s equation is the *backward equation*. See [13, Chapter 4]^4^.

### Definition 3.0.1

Fix T>0. Let $\xi : [0,T] \rightarrow \mathbb {R}$ be a càdlàg function, and $(\varphi _t)_{t \in [0,T]}$ the solution to Loewner’s equation with driving function ξ. Then the *backward equation* is the system of ordinary differential equations

3.3 $$\begin{aligned} u_0(z) = z, \quad \frac{\partial }{\partial t} u_t(z) = u_t(z) \frac{u_t(z) + e^{i\xi _{T-t}}}{u_t(z) - e^{i\xi _{T-t}}},\quad z \in \Delta , \end{aligned}$$

for t∈[0,T]. Then $u_T = \varphi _T$, but it is not usually true that $u_t = \varphi _t$ for t<T.

### Remark 12

It is often easier to analyse $u_t$ than $\varphi _t$ since the former is governed by an ordinary rather than partial differential equation.

### Remark 13

If the driving function ξ corresponds to an aggregation process, so $\xi _t = \theta _{\left\lfloor t/\textbf{c} \right\rfloor +1}$, then solving Loewner’s (forward) equation gives $\varphi _{N\textbf{c}} = f_1 \circ \dots \circ f_N$. Understanding $\varphi _{N\textbf{c}}(z)$, $\varphi _{N\textbf{c}}'(z)$, etc. requires analysing $f_N(z)$, $f_{N-1}(f_N(z))$, etc. For example, $\varphi _{N\textbf{c}}'(z) = \prod _{n=1}^{N} f_n'((f_{n+1}\circ \dots \circ f_N)(z))$, but the location of $(f_{n+1}\circ \dots \circ f_N)(z)$ is difficult to understand using the forward equation. However, if we solve the backward equation (3.3), we have $u_{n\textbf{c}} = f_{N-n+1} \circ \dots \circ f_N$ for all n≤N, so we could write $\varphi _{N\textbf{c}}'(z) = \prod _{n=1}^{N} f_n'(u_{(N-n)\textbf{c}}(z))$.

When keeping track of points on the circle, it is also useful to have estimates on *angular distortion* away from the tip and bases of the slit:

### Lemma 3.4

For any positive constant L>0, there is a constant $A_{L} > 0$ such that if $e^{i\alpha }$ and $e^{i\alpha '}$ are each at a distance at least *L* from 1 then

3.4 $$\begin{aligned} |f'(e^{i\alpha })| \le e^{A_L \textbf{c}} \end{aligned}$$

and

3.5 $$\begin{aligned} e^{-A_L \textbf{c}} | \alpha - \alpha ' | \le | \arg f(e^{i\alpha }) - \arg f(e^{i\alpha '}) | \le e^{A_L \textbf{c}} | \alpha - \alpha ' | \end{aligned}$$

for all sufficiently small $\textbf{c}$.

### Proof

The estimate (3.4) follows by expanding (3.2). When $|\theta | > \beta$, [1, equation (37)] says that

$$ 1 + \cos (\textrm{arg} f(e^{i\theta })) = (1 + \cos \theta )e^\textbf{c}$$

and this gives us (3.5). □

In [1], the results of which we extend here, the authors developed a useful set of estimates on the solutions $u^0$ and $u^1$ to (3.3) with respective driving functions $\xi ^0$ and $\xi ^1$.

### Lemma 3.5

(Lemma 11 of [1]) Suppose $z_0 \in {\Delta }$, T>0 and $\xi ^0:(0,T] \rightarrow \mathbb {R}$ are given and let

$$ \Lambda _t = \int _0^t \frac{2 |u_s^0(z_0)|^2 \textrm{d}s}{|(u_s^0)'(z_0)||u_s^0(z_0)e^{-i \xi ^0_{T-s}}-1|^2}. $$

There exists some absolute constant *A* such that, for all |z|>1 satisfying

3.6 $$\begin{aligned} |z-z_0|&\le A^{-1} \inf _{0 \le t \le T} \left( \frac{|u_t^0(z_0)e^{-i \xi ^0_{T-t}} -1|}{|(u_t^0)'(z_0)|} \wedge \left( \int _0^t \frac{|(u_s^0)'(z_0)| }{ |u_s^0(z_0)e^{-i \xi ^0_{T-s}} -1|^3} \textrm{d}s \right) ^{-1} \right) , \end{aligned}$$

we have, for all $0 \le t \le T$,

3.7 $$\begin{aligned} \left| \log \frac{u^0_t(z) - u^0_t(z_0)}{(z-z_0)(u^0_t)'(z_0)}\right| \le A |z-z_0| \int _0^t \frac{|(u^0_s)'(z_0)|\textrm{d}s}{|u_s^0(z_0)e^{-i \xi ^0_{T-s}} -1|^3} \end{aligned}$$

(where we interpret the left hand side as being equal to 0 if $z=z_0$) and

3.8 $$\begin{aligned} \left| \log \frac{(u^0_t)'(z)}{(u^0_t)'(z_0)} \right| \le A |z-z_0| \int _0^t \frac{|(u^0_s)'(z_0)|\textrm{d}s}{|u_s^0(z_0)e^{-i \xi ^0_{T-s}} -1|^3} . \end{aligned}$$

Furthermore, *A* can be chosen so that if, in addition, $\xi ^1:(0,T] \rightarrow \mathbb {R}$ satisfies

3.9 $$\begin{aligned} \Vert \xi ^1-\xi ^0\Vert _T \le \frac{1}{A} \inf _{0 \le t \le T} \left( \frac{|u_t^0(z_0)e^{-i \xi ^0_{T-t}} -1|}{|(u_t^0)'(z_0)|\Lambda _t+|u^0_t(z_0)|} \wedge \left( \int _0^t \frac{\Lambda _s|(u_s^0)'(z_0)| + |u^0_s(z_0)|}{ |u_s^0(z_0)e^{-i \xi ^0_{T-s}} -1|^3} \textrm{d}s \right) ^{-1} \right) , \end{aligned}$$

then, for all $0 \le t \le T$,

3.10 $$\begin{aligned} \left| u^1_t(z)-u^0_t(z) \right| \le A|(u^0_t)'(z_0)| \Vert \xi ^1 - \xi ^0\Vert _T \Lambda _t \end{aligned}$$

and

3.11 $$\begin{aligned} \left| \log \frac{(u^1_t)'(z)}{(u^0_t)'(z)} \right| \le A \Vert \xi ^1-\xi ^0\Vert _T \int _0^t \frac{\Lambda _s|(u_s^0)'(z_0)| + |u^0_s(z_0)|}{ |u_s^0(z_0)e^{-i \xi ^0_{T-s}} -1|^3} \textrm{d}s. \end{aligned}$$

## Removing the regularisation

In this section we reduce the ALE, which has an attachment density supported on $\mathbb {T}$, to the “multinomial model,” which, like the LPM, grows only at the tips, and the probability of attaching to a tip at time *t* is determined by the second derivative of the map corresponding to the cluster.

The proofs in this section mostly involve careful calculations to estimate $|\Phi _n'|$ around relevant points.

### ALE to auxiliary model

First we define the auxiliary model, which is a version of the ALE in which growth occurs exactly at the tips. This first step is a type of dimension reduction, in which we restrict possible attachment points from all of $\mathbb {T}$ to only *k* points.

#### Definition 4.1.1

Let $K_0$ be of the same form as above, and $\Phi _0^{*} = \Phi _0^{\textrm{LPM}}$. We will choose an attachment point θ as we do for the ALE, but before attaching a particle at θ, we rotate the entire cluster so that θ is the preimage of the tip of a slit. We define the map $\Phi _n^*$ of the auxiliary model inductively: At step *n* we still have a configuration of *k* curves (which are not necessarily line segments). Let the preimage under $\Phi _n^*$ of the tip of the *j*th curve be $\exp (i\bar{\phi }^j_n)$. Choose $\theta _{n+1}^*$ according to the conditional density

$$\begin{aligned} h(\theta \,|\, \theta _1, \dots , \theta _n) = \frac{1}{Z_n^*} | (\Phi _t^*)'(e^{\sigma + i\theta }) |^{-\eta }, \end{aligned}$$

and if $j_n = \mathop {\textrm{argmin}}_j |e^{i\theta _{n+1}^*} - e^{i\bar{\phi }^j_n}|$ (which almost surely uniquely exists), let $\hat{\theta }_{n+1}^* = \bar{\phi }^{j_n}_n$, and $\delta _{n+1} = \theta _{n+1}^* - \hat{\theta }_{n+1}^*$.

Then if $R_{\theta }(z) = e^{i\theta }z$, set

$$\begin{aligned} \Phi _{n+1}^* = R_{\delta _{n+1}} \circ \Phi _n^* \circ R_{-\delta _{n+1}} \circ f_{\theta _{n+1}^*}. \end{aligned}$$

#### Remark 14

**Fig. 2:**
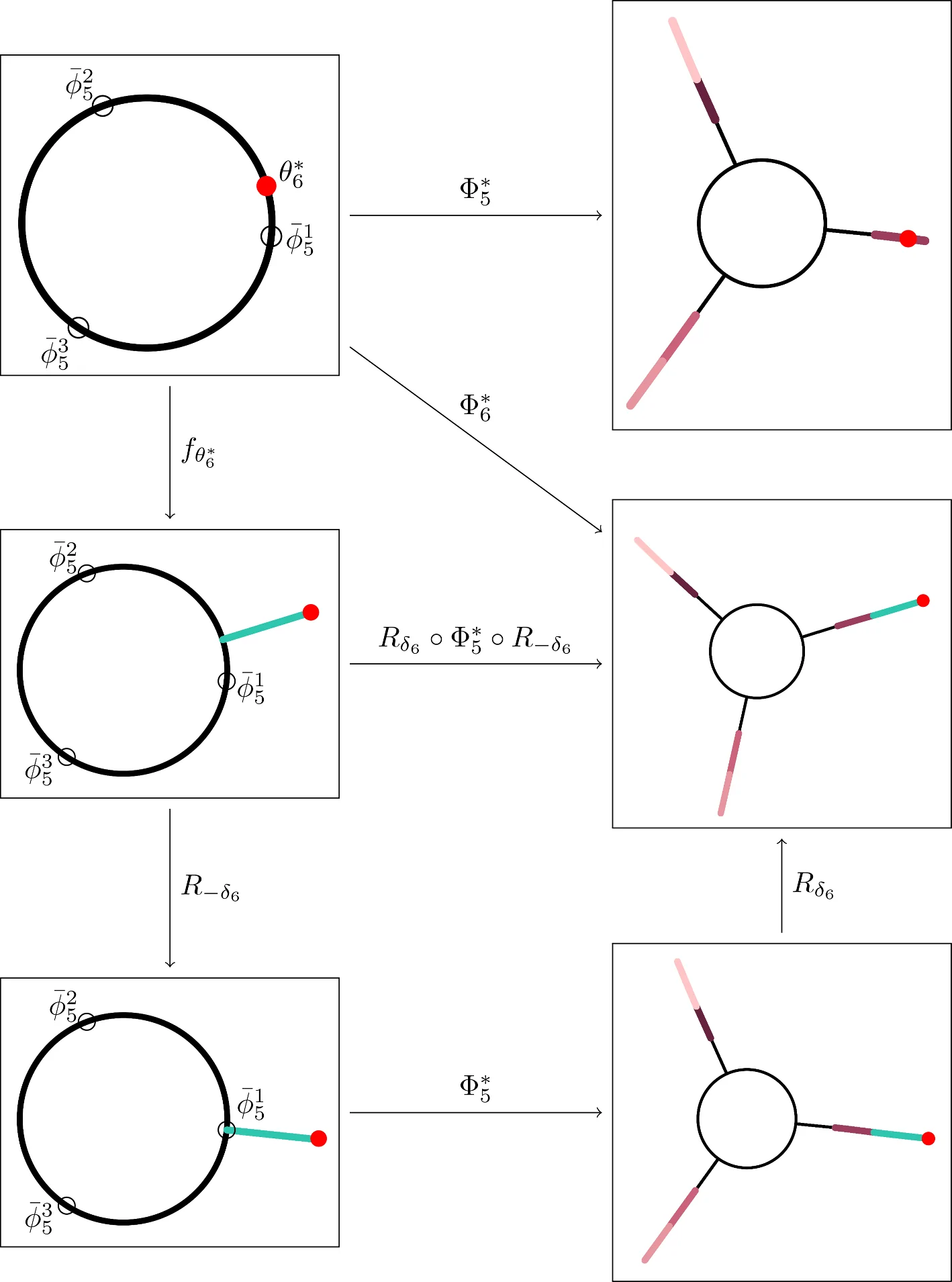
One step in the construction of the auxiliary model. The large dot at one tip represents the image of $\exp (i \theta _{6}^*)$ under each map. Comparing $K_5$ and $K_6$, we can see that both have three curves but the attachment points have been rotated from one cluster to the next

This procedure of choosing the (n+1)th angle and attaching a slit to a rotated version of the *n*th cluster is illustrated in Figure 2.

#### Remark 15

Since $f_\theta \circ R_{-\delta } = R_{-\delta } \circ f_{\theta +\delta }$, we can write $\Phi ^{*}_n$ in two ways:

4.1 $$\begin{aligned} \Phi ^{*}_n&= R_{\delta _1 + \dots + \delta _n} \circ \Phi _0 \circ R_{-\delta _1} \circ f_{\theta _1^*} \circ R_{-\delta _2} \circ \dots \circ R_{-\delta _n} \circ f_{\theta _n^*}\nonumber \\&= R_{\delta _1 + \dots + \delta _n} \circ \Phi _0 \circ R_{-(\delta _1 + \dots + \delta _n)} \circ f_{\theta _1^* + \delta _2 + \delta _3 + \dots + \delta _n} \circ f_{\theta _2^* + \delta _3 + \dots + \delta _n} \circ \dots \circ f_{\theta _n^*}, \end{aligned}$$

and this latter expression is also equal to

$$\begin{aligned} R_{\delta _1 + \dots + \delta _n} \circ \Phi _0 \circ R_{-(\delta _1 + \dots + \delta _n)} \circ f_{\hat{\theta }_1^* + \delta _1 + \dots + \delta _n} \circ f_{\hat{\theta }_2^* + \delta _2 + \dots + \delta _n} \circ f_{\hat{\theta }_n^* + \delta _n}. \end{aligned}$$

#### Definition 4.1.2

We denote the preimages of the *j*th tip by $e^{i\phi ^j_n}$ in the ALE model, and by $e^{i\bar{\phi }^j_n}$ in the auxiliary model. Define

$$\begin{aligned} j_n&:= \mathop {\textrm{argmin}}\limits _j | e^{i\theta _n} - e^{i\phi ^j_n} |,\\ j_n^*&:= \mathop {\textrm{argmin}}\limits _j | e^{i\theta _n^*} - e^{i\bar{\phi }^j_n} |. \end{aligned}$$

If we construct a version of each of the ALE and auxiliary models on a common probability space, then we can define the stopping time

$$\begin{aligned} \tau _{\textrm{coupling}}:= \min \{ n \ge 1 : j_n \not = j_n^* \}. \end{aligned}$$

To show that the ALE and auxiliary models are close, we will couple the models so that at each step the probability $j_n = j_n^*$ is close to 1, and show that with high probability the ALE lands very near a tip.

First we will examine the regions near the tips in each model, and find that the probability of landing near each tip is similar. Then we will prove in each case that the probability of not landing near any tip is *o*(1), generalising the main result of [1], with a similar argument.

#### Definition 4.1.3

The coupling relies on both models attaching near tips, so we define another stopping time which triggers when a particle in either model is too far from its nearest tip. Let

$$\begin{aligned} \tau _D = \inf \{ n \ge 1 : |\theta _n^* - \hat{\theta }_n^*|> D {\text { or }} \min _{j} |\theta _n - \phi ^j_n| > D \}, \end{aligned}$$

where $$D = \sqrt{\sigma }$$.

We will establish a bound on $\mathbb {P}[ \tau _D < n ]$ which does not depend on the exact nature of the coupling, and then use this bound to show that a coupling exists under which $\mathbb {P}[ \tau _{\textrm{coupling}}\le \left\lfloor T/\textbf{c} \right\rfloor ]$ is small.

#### Remark 16

**Fig. 3:**
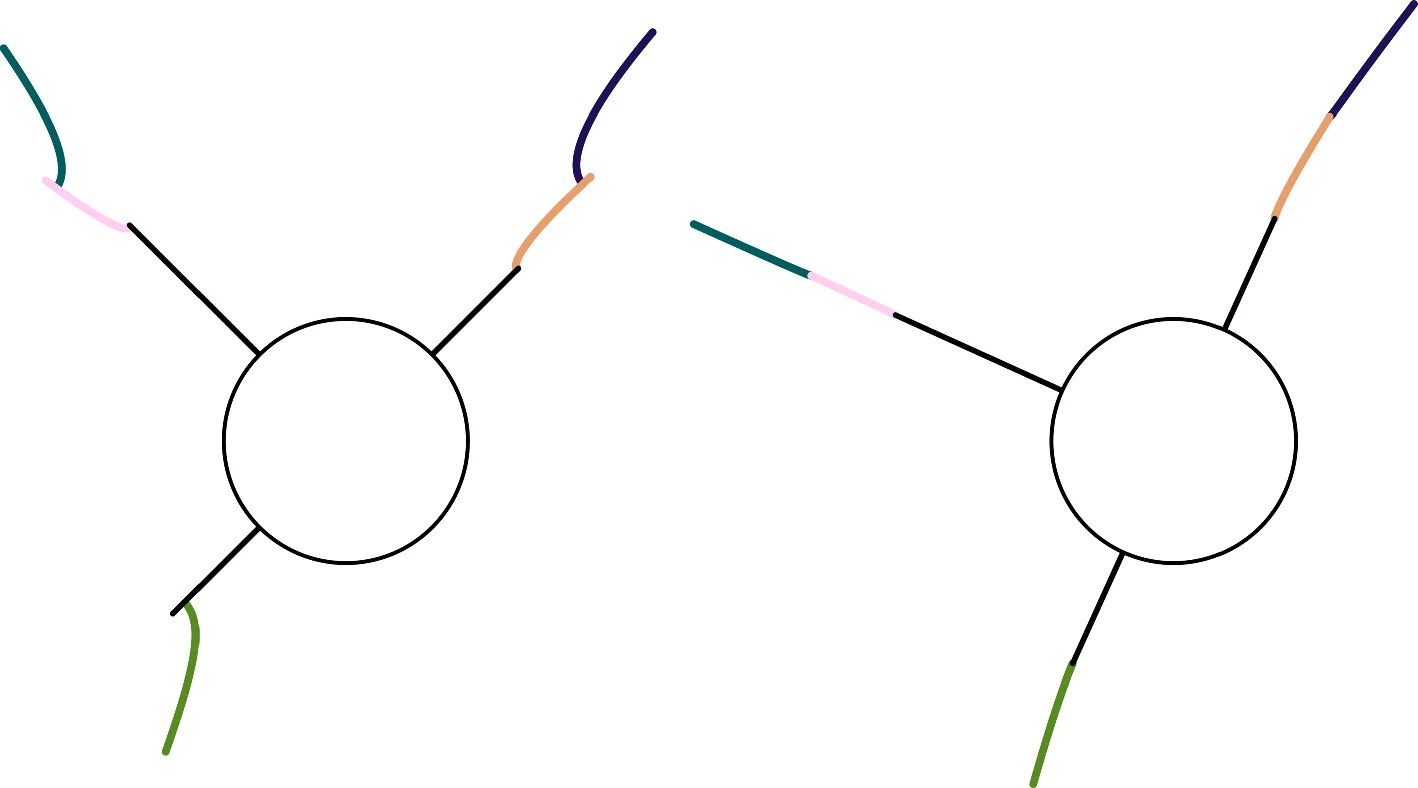
Coupled ALE (left) and auxiliary (right) models at a time $n < \tau _D \wedge \tau _{\textrm{coupling}}$ started from a three-arm configuration. Note the slight rotation of three initial arms in the auxiliary model, and that the curves are repelled from each other

The ALE and auxiliary models will have a common weak limit if with high probability $\tau _D \wedge \tau _{\textrm{coupling}}> T/\textbf{c}$. This event means that every particle in the ALE model has been attached within *D* of a “main” tip, and every particle in the auxiliary model has chosen the same slit to attach to as the ALE model. An example of the coupling of these two models is shown in Figure 3.

#### Theorem 4.1

There exists a constant $A = A(K_0, k, \eta , T)$ such that the ALE and auxiliary models can be constructed on a common probability space and on this space

$$\begin{aligned} \mathbb {P}(\tau _D \wedge \tau _{\textrm{coupling}}\le \left\lfloor T/\textbf{c} \right\rfloor ) \le A \textbf{c}^{1/2} \end{aligned}$$

provided $$\sigma < \textbf{c}^{\gamma }$$ for $$\gamma = \frac{2(\eta +2)}{\eta -1} \vee \frac{5\eta + 10}{2\eta } \vee 8$$.

#### Remark 17

The three terms determining γ come from three separate requirements in the proof of this section’s result: we require $\sigma < \textbf{c}^8$ so that the derivatives of each model look similar near the “main” tips, as in Proposition 4.3. We require that $\sigma < \textbf{c}^{\frac{5\eta +10}{2\eta }}$ so that “old” particles do not contribute in the ALE model, and we require that $\sigma < \textbf{c}^{\frac{2(\eta +2)}{\eta -1}}$ so that the measures are concentrated very tightly around the tip of each particle, with each particle attached within distance *D* of a main tip.

**Fig. 4:**
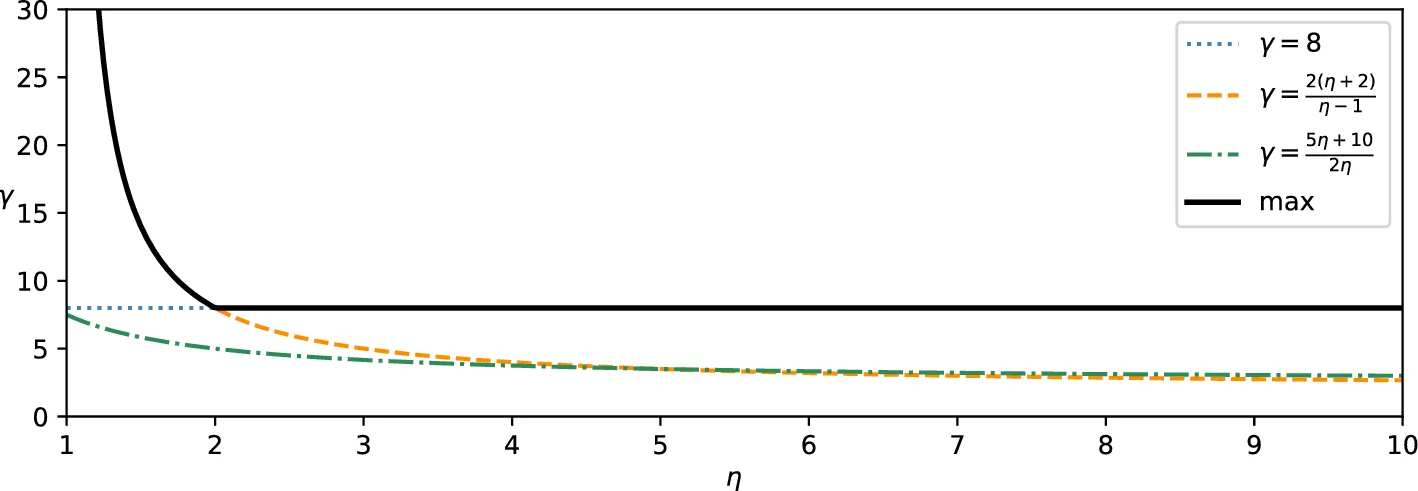
A plot of the exponent γ used to control the regularisation in Theorem 4.1 as a function of the parameter η. Each of the three terms determining γ is plotted separately, with their maximum shown as a solid black curve

All three terms and their maximum are plotted in Figure 4.

In fact, the $\frac{5\eta + 10}{2\eta }$ term is redundant since it is always less than 8, but we retain it because the assumption $\gamma \ge \frac{5\eta + 10}{2\eta }$ makes some of the arguments clearer, as it corresponds to one of the exponents in Proposition 4.2.

#### Remark 18

It would be possible to use a slightly smaller γ if we replaced the $A\textbf{c}^{1/2}$ with a weaker *o*(1) upper bound in Theorem 4.1 (which is all we require to prove Theorem 2.1) but we have left it in its current form to avoid complicating the proofs further.

The proof of Theorem 4.1 will follow from a proposition controlling $\tau _D$ and another controlling $\tau _D \wedge \tau _{\textrm{coupling}}$, both of which we state now.

#### Proposition 4.2

For any n<N,

$$\begin{aligned} \mathbb {P}[ n+1 = \tau _D \,|\, n < \tau _D ] \le A \textbf{c}^{-\eta } \sigma ^{\frac{\eta -1}{2}} + A \textbf{c}^{-2\eta }\sigma ^{\eta -1} + A \textbf{c}^{-\frac{5\eta }{4} - \frac{1}{2}} \sigma ^{\eta /2}. \end{aligned}$$

If $\sigma < \textbf{c}^{\gamma }$ where $\gamma = \frac{2(\eta +2)}{\eta -1} \vee \frac{5\eta + 10}{2\eta } \vee 8$, then this implies $\mathbb {P}[ n+1 = \tau _D \,|\, n < \tau _D ] \le A \textbf{c}^2$ and so $\mathbb {P}[ \tau _D \le \left\lfloor T/\textbf{c} \right\rfloor ] \le AT\textbf{c}$.

#### Proposition 4.3

Assume that $\sigma < \textbf{c}^8$. We can construct the coupling of the ALE and auxiliary models in such a way that for all $n \le T/\textbf{c}$, on the event $\{n < \tau _D \wedge \tau _{\textrm{coupling}}\}$, the conditional probability that $j_{n+1} \not = \bar{j}_{n+1}$ or $n+1 = \tau _D$ is almost surely bounded by

$$\begin{aligned} ATk\textbf{c}^{3/2} + \mathbb {P}\left[ \delta _{n+1}> D \,|\, n< \tau _D \right] + \mathbb {P}\left[ \min _j |\theta _{n+1} - \phi ^j_n | > D \,\bigg |\, n < \tau _D \right] . \end{aligned}$$

#### Proof of Theorem 4.1

Note

$$ \mathbb {P}(\tau _D \wedge \tau _{\textrm{coupling}}\le \left\lfloor T/\textbf{c} \right\rfloor ) \le \mathbb {P}(\tau _D \le \left\lfloor T/\textbf{c} \right\rfloor ) + \mathbb {P}(\tau _{\textrm{coupling}}\le \left\lfloor T/\textbf{c} \right\rfloor \,|\, \tau _D > \left\lfloor T/\textbf{c} \right\rfloor ). $$

By Proposition 4.2, the first term in the sum above is less than $A \textbf{c}$ under any coupling. Then using Proposition 4.3 we can construct a coupling such that for any $n \le \left\lfloor T/\textbf{c} \right\rfloor$,

$$\begin{aligned} \mathbb {P}( \tau _{\textrm{coupling}}= n+1 \,|\, n< \tau _{\textrm{coupling}}{\text { and }} \tau _D > \left\lfloor T/\textbf{c} \right\rfloor )&\le A\textbf{c}^{3/2} + 2 \mathbb {P}( n+1 = \tau _D \,|\, n < \tau _D ), \end{aligned}$$

and the second term is bounded by $A\textbf{c}^2$. Therefore, summing over $n < \left\lfloor T/\textbf{c} \right\rfloor$,

$$ \mathbb {P}( \tau _{\textrm{coupling}}\le \left\lfloor T/\textbf{c} \right\rfloor \,|\, \tau _D > \left\lfloor T/\textbf{c} \right\rfloor ) \le A\textbf{c}^{1/2} $$

as required. □

Now we have to prove Propositions 4.2 and 4.3. We begin with Proposition 4.3 as its proof is somewhat simpler.

#### Proof of Proposition 4.3

The result follows simply if we can prove the claim: for $n \le T/\textbf{c}$, on the event $\{n < \tau _D \wedge \tau _{\textrm{coupling}}\}$,

$$\begin{aligned} \sup _{|\theta | < \textbf{c}^2} \left| \log \frac{ (\Phi _n^{\textrm{ALE}})'(e^{\sigma + i\theta }e^{i\phi ^j_n}) }{ (\Phi _n^*)'(e^{\sigma + i\theta }e^{i\bar{\phi }^j_n}) } \right| \le AT\textbf{c}^{3/2} \end{aligned}$$

almost surely.

Denote the preimages under $\Phi ^{\textrm{ALE}}_n$ of the *k* tips in the ALE model by $\phi ^1_n, \dots , \phi ^k_n$. For each $j \in \{1,2,\dots ,k\}$ denote the subsequence of $(\theta ^{\textrm{ALE}}_\ell )_{\ell = 1}^n$ consisting of the times at which a particle is attached to the *j*th slit by

$$ \theta ^{\textrm{ALE}}_{n_j(1)}, \theta ^{\textrm{ALE}}_{n_j(2)}, \dots , \theta ^{\textrm{ALE}}_{n_j(N_j)}. $$

Near $\phi _n^j$ we can decompose $\Phi ^{\textrm{ALE}}_n$ as

4.2 $$\begin{aligned} \Phi ^{\textrm{ALE}}_n = \Phi ^{\textrm{ALE}}_0 \circ \Psi _{0}^j \circ f^{\textrm{ALE}}_{n_j(1)} \circ \Psi ^j_1 \circ f^{\textrm{ALE}}_{n_j(2)} \circ \Psi ^j_2 \circ \dots \circ f^{\textrm{ALE}}_{n_j(N_j)} \circ \Psi ^j_{N_j}, \end{aligned}$$

where for $0< \ell < N_j$, $\Psi ^j_\ell = f^{\textrm{ALE}}_{n_j(\ell )+1} \circ f^{\textrm{ALE}}_{n_j(\ell )+2} \circ \dots \circ f^{\textrm{ALE}}_{n_j(\ell +1)-1} = \left( \Phi ^{\textrm{ALE}}_{n_j(\ell )}\right) ^{-1} \circ \Phi ^{\textrm{ALE}}_{n_j(\ell +1)-1}$, the map which attaches every particle landing at slits *other than* the *j*th between the ℓth and (ℓ+1)th time a particle lands on the *j*th slit. The two other maps are $\Psi ^j_{0} = \left( \Phi ^{\textrm{ALE}}_{0}\right) ^{-1} \circ \Phi ^{\textrm{ALE}}_{n_j(1)-1}$ and $\Psi ^j_{N_j} = \left( \Phi ^{\textrm{ALE}}_{n_j(N_j)}\right) ^{-1} \circ \Phi ^{\textrm{ALE}}_{n}$, which correspond to the particles before the first attachment and after the last attachment to the *j*th slit, respectively. Note that any of these $\Psi ^j_\ell$ maps may be the identity map on Δ if $n_j(\ell +1) = n_j(\ell ) + 1$. The important common feature of all these $\Psi ^j_\ell$ maps is that their derivatives have no poles or zeroes near the *j*th slit.

Since the event we have conditioned on implies $n < \tau _{\textrm{coupling}}$, the subsequence of $(\theta ^{*}_\ell )_{\ell = 1}^{n}$ consisting of times when a particle was attached to the *j*th slit is

$$ \theta ^{*}_{n_j(1)}, \theta ^{*}_{n_j(2)}, \dots , \theta ^{*}_{n_j(N_j)} $$

with the same $n_j$ as above. There is a similar decomposition to (4.2), complicated only slightly by the cluster rotation. Let $\Delta _{l}^{n}:= \delta _l+\delta _{k+1}+\dots +\delta _n$. Recall that $\theta ^{*}_l = \hat{\theta }^*_l + \delta _l$. Then

$$\begin{aligned} \Phi ^{*}_n&= R_{\Delta _1^n} \circ \Phi ^{*}_0 \circ R_{-\Delta _{1}^{n}}\circ \\&\phantom {==}\circ \overline{\Psi }^j_0 \circ f^{*}_{\hat{\theta }^*_{n_j(1)} + \Delta _{n_j(1)}^{n}} \circ \overline{\Psi }^j_1 \circ \dots \circ f^{*}_{\hat{\theta }^*_{n_j(N_j)} + \Delta _{n_j(N_j)}^{n}} \circ \overline{\Psi }^j_{N_j}, \end{aligned}$$

where $\overline{\Psi }^j_\ell = f^{*}_{\hat{\theta }^*_{n_j(\ell )+1}+\Delta _{n_j(\ell )+1}^n} \circ \dots \circ f^{*}_{\hat{\theta }^*_{n_j(\ell +1)-1}+\Delta _{n_j(\ell +1)-1}^{n}}$ for $0< \ell < N_j$ and the two end-cases are also defined similarly to $\Psi ^j_0$ and $\Psi ^j_{N_j}$.

Now fix $\theta \in (-\textbf{c}^2,\textbf{c}^2)$ and we will compare the densities $|(\Phi ^{\textrm{ALE}}_n)'(e^{\sigma + i \theta }e^{i\phi ^j_n})|^{-\eta }$ and $|(\Phi ^{*}_n)'(e^{\sigma + i \theta }e^{i\bar{\phi }^j_n})|^{-\eta }$ when $N_j > 0$. We omit the simpler case $N_j = 0$.

First we will bound

$$ \left| \log \frac{ (\Psi ^j_{N_j})'(e^{\sigma + i \theta }e^{i\phi ^j_n}) }{ (\overline{\Psi }^j_{N_j})'(e^{\sigma + i \theta }e^{i\bar{\phi }^j_n}) } \right| . $$

The two maps $\Psi ^j_{N_j}$ and $\overline{\Psi }^j_{N_j}$ are solutions to Loewner’s equation with driving functions

$$ \xi _t = \theta ^{\textrm{ALE}}_{\left\lfloor t/\textbf{c} \right\rfloor +n_j(N_j)+1}, \quad \overline{\xi }_t = \hat{\theta }^*_{\left\lfloor t/\textbf{c} \right\rfloor +n_j(N_j)+1} + \Delta _{\left\lfloor t/\textbf{c} \right\rfloor +n_j(N_j)+1}^n $$

respectively, for $t \in [0, (n-n_j(N_j))\textbf{c})$. Remark 3.7 of [7] notes that the points $e^{i\bar{\phi }^j_n}$ are repelled from each other on the circle. We therefore know that there is an L>0 depending only on the initial conditions and *T* such that $e^{\sigma +i\theta }e^{i\phi ^j_n}$ and $e^{\sigma + i \theta }e^{i\bar{\phi }^j_n}$ keep at least a distance *L* away from $\xi _t$ and $\overline{\xi }_t$ respectively. We will apply Lemma 3.5, so note $\Lambda _t \asymp t$, condition (3.9) is met for sufficiently small $\textbf{c}$, and the bound on the right of (3.6) is ≍1.

We know $e^{i \phi ^j_n} = \left( \Psi ^j_{N_j} \right) ^{-1}( \theta ^{\textrm{ALE}}_{n_j(N_j)} )$ and $e^{i \bar{\phi }^j_n} = \left( \overline{\Psi }^j_{N_j} \right) ^{-1}( \hat{\theta }^*_{n_j(N_j)} + \Delta _{n_j(N_j)}^n )$, and so applying Lemma 3.5 gives us, since $(n-n_j(N_j))\textbf{c}\le T$,

$$\begin{aligned} \left| \log \frac{ (\Psi ^j_{N_j})'(e^{\sigma + i\theta } e^{i\phi _n^j}) }{ (\overline{\Psi }^j_{N_j})'(e^{\sigma + i\theta } e^{i\bar{\phi }_n^j}) } \right|&\le A_L T \left( \left| \phi _n^j - \bar{\phi }_n^j \right| + |\delta _1| + \dots + |\delta _{n-1}| \right) , \end{aligned}$$

and a further calculation using explicit estimates for $f^{-1}$ similar to (3.5) tells us

$$ |\phi _n^j - \bar{\phi }_n^j| \le A_L (|\delta _1| + \dots + |\delta _n|). $$

Hence, with a constant *A* depending on $A_L$ and *T*,

4.3 $$\begin{aligned} \left| \log \frac{ (\Psi ^j_{N_j})'(e^{\sigma + i\theta } e^{i\phi _n^j}) }{ (\overline{\Psi }^j_{N_j})'(e^{\sigma + i\theta } e^{i\bar{\phi }_n^j}) } \right|&\le A \left( |\delta _1| + \dots + |\delta _{n}| \right) . \end{aligned}$$

The next two terms in the expansions of each derivative are the two largest,

4.4 $$\begin{aligned} (f^{\textrm{ALE}}_{n_j(N_j)})' \left( \Psi ^j_{N_j}(e^{\sigma + i\theta } e^{i\phi _n^j})\right) \quad {\text {and}}\quad (f^{*}_{\hat{\theta }^*_{n_j(N_j)} + \Delta _{n_j(N_j)}^{n}})' \left( \overline{\Psi }^j_{N_j}(e^{\sigma + i\theta } e^{i\bar{\phi }_n^j})\right) . \end{aligned}$$

It is clear from (3.2) that, to first order, the size of $|f^{\prime }\left(z\right)|$ depends on |z-1|. This means that to find the size of the two derivatives above, we are most interested in

$$ \left| \Psi ^j_{N_j}(e^{\sigma + i\theta } e^{i\phi _n^j}) - e^{i\theta ^{\textrm{ALE}}_{n_j(N_j)}}\right| \quad {\text {and}}\quad \left| \overline{\Psi }^j_{N_j}(e^{\sigma + i\theta } e^{i\bar{\phi }_n^j}) - e^{i(\hat{\theta }^*_{n_j(N_j)} + \Delta _{n_j(N_j)}^n) } \right| . $$

If we apply (3.7), the first of these is

4.5 $$\begin{aligned} \left| \Psi ^j_{N_j}(e^{\sigma + i\theta } e^{i\phi _n^j}) - e^{i\theta ^{\textrm{ALE}}_{n_j(N_j)}} \right|&= \left| \Psi ^j_{N_j}(e^{\sigma + i\theta } e^{i\phi _n^j}) - \Psi ^j_{N_j}(e^{i\phi _n^j}) \right| \nonumber \\&= \left| (e^{\sigma +i\theta }-1)e^{i\phi _n^j} (\Psi ^j_{N_j})'(e^{i\phi _n^j}) \right| \left[ 1 + O\left( |e^{\sigma +i\theta }-1| \right) \right] \nonumber \\&= |e^{\sigma + i\theta } - 1| \left| (\Psi ^j_{N_j})'(e^{i\phi _n^j})\right| \left[ 1 + O\left( |e^{\sigma +i\theta }-1| \right) \right] \end{aligned}$$

and similarly the second term is

4.6 $$\begin{aligned} |e^{\sigma + i\theta } - 1| \left| (\overline{\Psi }^j_{N_j})'(e^{i\bar{\phi }_n^j})\right| \left[ 1 + O\left( |e^{\sigma +i\theta }-1| \right) \right] . \end{aligned}$$

Our previous bounds on $\left| \log \frac{ (\Psi ^j_{N_j})'(e^{\sigma + i\theta } e^{i\phi _n^j}) }{ (\overline{\Psi }^j_{N_j})'(e^{\sigma + i\theta } e^{i\bar{\phi }_n^j}) }\right|$ apply equally to $\left| \log \frac{ (\Psi ^j_{N_j})'(e^{i\phi _n^j}) }{ (\overline{\Psi }^j_{N_j})'(e^{i\bar{\phi }_n^j}) }\right|$, so (3.2) allows us to directly compare the derivatives in (4.4).

Firstly, a calculation using the explicit form of *f*(*z*) in (3.1) shows that if $$|z-1| < \textbf{c}^{1/2}$$ then f(z)=f(1)+O(|z-1|)=1+d+O(|z-1|). The next estimate z=1+O(|z-1|) is obvious. Putting this estimate into the denominator of the second fraction in (3.2), we get $$|z - e^{\pm i \beta }|^{1/2} = |1 - e^{i\beta }|^{1/2} (1 + O\left( \frac{|z-1|}{\textbf{c}^{1/2}}\right) )$$. Thus for *z* close to 1 the behaviour of $|f^{\prime }\left(z\right)|$ mainly depends on |z-1|, and we can write

$$\begin{aligned} |f'(z)| = \frac{1+d(\textbf{c})}{|1-e^{i\beta }|}\, |z-1| \left( 1 + O(\textbf{c}^{-1/2} |z-1|) \right) . \end{aligned}$$

Therefore

4.7 $$\begin{aligned} \left| \log \frac{ (f^{\textrm{ALE}}_{n_j(N_j)})'\left( \Psi ^j_{N_j}(e^{\sigma + i\theta } e^{i\phi _n^j}) \right) }{ (f^{*}_{\hat{\theta }^*_{n_j(N_j)} + \Delta _{n_j(N_j)}^{n}})'\left( \overline{\Psi }^j_{N_j}(e^{\sigma + i\theta } e^{i\bar{\phi }_n^j}) \right) } \right|&\le \left| \log \frac{ \left| (\Psi ^j_{N_j})'(e^{i\phi _n^j})\right| }{ \left| (\overline{\Psi }^j_{N_j})'(e^{i\bar{\phi }_n^j})\right| } \right| + A \frac{|e^{\sigma + i\theta } - 1|}{\textbf{c}^{1/2}} \nonumber \\&\le A \left( |\delta _1| + \dots + |\delta _{n}| + \frac{|e^{\sigma + i\theta } - 1|}{\textbf{c}^{1/2}} \right) . \end{aligned}$$

Now as $|f^{\textrm{ALE}}_{n_j(N_j)}(\Psi _{N_j}(e^{\sigma + i\theta }e^{i\phi _{n}^{j}}))| \ge 1 + \textbf{c}^{1/2}$ and $|f^{*}_{n_j(N_j)}(\overline{\Psi }_{N_j}(e^{\sigma + i\theta }e^{i\bar{\phi }_{n}^{j}}))| \ge 1 + \textbf{c}^{1/2}$, we can apply Lemma 3.5 to compare the two remaining derivatives

$$ \left| \left( \Phi ^{\textrm{ALE}}_0 \circ \Psi _{0}^j \circ f^{\textrm{ALE}}_{n_j(1)} \circ \dots \circ \Psi _{N_j-1}^j \right) '\left( f^{\textrm{ALE}}_{n_j(N_j)}(\Psi _{N_j}(e^{\sigma + i\theta }e^{i\phi _{n}^{j}})) \right) \right| $$

and

$$\begin{aligned} \Bigg | \left( R_{\Delta _1^n} \circ \Phi ^{*}_0 \circ R_{-\Delta _1^n} \circ \overline{\Psi }^j_0 \circ f^{*}_{\hat{\theta }^*_{n_j(1)} + \Delta _{n_j(1)}^{n}} \circ \dots \circ \overline{\Psi }^j_{N_j-1} \right) ' \Big (f^{*}_{n_j(N_j)}(\overline{\Psi }_{N_j}(e^{\sigma + i\theta }e^{i\bar{\phi }_{n}^{j}})) \Big ) \Bigg |. \end{aligned}$$

The two maps above whose derivatives we consider can be generated by the backward equation using driving functions which we will call $\xi ^0$ (generating $\Phi ^{\textrm{ALE}}_0 \circ \dots \circ \Psi _{N_j-1}^j$) and $\xi ^1$ (generating $R_{\Delta _1^n} \circ \dots \circ \overline{\Psi }^j_{N_j-1}$). We can now use Lemma 3.5 to compare the above derivatives. We have the bound $\Vert \xi ^0 - \xi ^1 \Vert _{\infty } \le |\delta _1| + \dots + |\delta _n|$. Taking $z_0 = f^{\textrm{ALE}}_{n_j(N_j)}(\Psi _{N_j}(e^{\sigma + i\theta }e^{i\phi _{n}^{j}}))$, we have $|z_0| - 1 \ge \textbf{c}^{1/2}$, so using standard estimates for conformal maps [3, Chapter 4.4, Corollary 6] we have $\frac{\textbf{c}^{1/2}}{A} \le |(u_t^0)'(z_0)| \le \frac{A}{\textbf{c}^{1/2}}$ for all 0<t<T, where the constant *A* depends only on *T* and $K_0$.

This gives us, modifying the constant $A = A(T, K_0)$ appropriately (as we will throughout), $\frac{\textbf{c}^{1/2}}{A} t \le |\Lambda _t| \le \frac{A}{\textbf{c}^{3/2}}t$.

The right-hand side of (3.6) is bounded below by

$$\begin{aligned} \inf _{0 < t \le T} \left( \frac{\textbf{c}^{1/2}}{A \textbf{c}^{-1/2}} \wedge \left( t \frac{A\textbf{c}^{-1/2}}{\textbf{c}^{3/2}} \right) ^{-1} \right) \ge A^{-1} \textbf{c}^{2}, \end{aligned}$$

so the condition (3.6) is satisfied by $z = f^{*}_{\hat{\theta }^*_{n_j(N_j)} + \Delta _{n_j(N_j)}^n}( \overline{\Psi }^j_{N_j}(e^{\sigma + i\theta } e^{i\bar{\phi }_n^j}) )$.

The resulting bounds on $\left| \log \frac{(u_t^0)'(z)}{(u_t^0)'(z_0)} \right|$ and $\left| \log \frac{(u_t^1)'(z)}{(u_t^0)'(z)} \right|$ are then, by Lemma 3.5,

$$ \left| \log \frac{(u_t^0)'(z)}{(u_t^0)'(z_0)} \right| \le A |z-z_0| t \textbf{c}^{-2} $$

and

$$ \left| \log \frac{(u_t^1)'(z)}{(u_t^0)'(z)} \right| \le A (|\delta _1| + \dots + |\delta _n|) \textbf{c}^{-7/2}. $$

Expanding (3.1) for *w* close to 1, we can estimate

4.8 $$\begin{aligned} f(w) = 1 + d(\textbf{c}) + O\left( \frac{|w - 1|^2}{\textbf{c}^{1/2}} + |w-1| \right) , \end{aligned}$$

so using (4.5) and (4.6),

$$\begin{aligned} |z - z_0|&\le (1+d(\textbf{c})) \left| \theta ^{\textrm{ALE}}_{n_j(N_j)} - (\hat{\theta }^*_{n_j(N_j)} + \Delta _{n_j(N_j)}^n) \right| \\&\phantom {\le } + A \frac{|e^{\sigma + i\theta } - 1|^2}{\textbf{c}^{1/2}} \left( \left| (\Psi ^j_{N_j})'(e^{i\phi _n^j})\right| + \left| (\overline{\Psi }^j_{N_j})'(e^{i\bar{\phi }_n^j})\right| \right) \\&\le A( |\delta _1| + \dots + |\delta _n| + \textbf{c}^{-1/2} |e^{\sigma + i\theta } - 1|^2 ). \end{aligned}$$

Hence

$$\begin{aligned} \left| \log \frac{ \left( R_{\Delta _1^n} \circ \Phi ^{*}_0 \circ R_{-\Delta _1^n} \circ \dots \circ \overline{\Psi }^j_{N_j-1} \right) '\left( f^{*}_{n_j(N_j)}(\overline{\Psi }_{N_j}(e^{\sigma + i\theta }e^{i\bar{\phi }_{n}^{j}})) \right) }{ \left( \Phi ^{\textrm{ALE}}_0 \circ \Psi _{0}^j \circ f^{\textrm{ALE}}_{n_j(1)} \circ \dots \circ \Psi _{N_j-1}^j \right) '\left( f^{\textrm{ALE}}_{n_j(N_j)}(\Psi _{N_j}(e^{\sigma + i\theta }e^{i\phi _{n}^{j}})) \right) } \right| \end{aligned}$$

is

4.9 $$\begin{aligned} \left| \log \frac{ (u_T^1)'(z ) }{ (u_T^0)'(z_0) } \right|&\le \left| \log \frac{ (u_T^1)'(z) }{ (u_T^0)'(z) } \right| + \left| \log \frac{ (u_T^0)'(z ) }{ (u_T^0)'(z_0) } \right| \nonumber \\&\le A \left( (|\delta _1| + \dots + |\delta _n|)\textbf{c}^{-7/2} + |e^{\sigma + i\theta } - 1|^2 \textbf{c}^{-5/2} \right) . \end{aligned}$$

Putting together (4.3), (4.7) and (4.9) we have

$$\begin{aligned} \left| \log \frac{ (\Phi ^{\textrm{ALE}}_n)'(e^{\sigma + i\theta }e^{i\phi _{n}^{j}}) }{ (\Phi ^{*}_n)'(e^{\sigma + i\theta }e^{i\bar{\phi }_{n}^{j}}) } \right|&\le A \left( \frac{|\delta _1| + \dots + |\delta _n|}{\textbf{c}^{7/2}} + \frac{|e^{\sigma + i\theta } - 1|}{\textbf{c}^{1/2}} + \frac{|e^{\sigma + i\theta } - 1|^2}{\textbf{c}^{5/2}} \right) \end{aligned}$$

and we can check that this is bounded by $AT \textbf{c}^{3/2}$, so our claim and the result follow. □

The following three lemmas will be used to prove Proposition 4.2.

First we show that the probability of attaching more than distance *D* from *any* tip is $o(\textbf{c})$ in each model. We will demonstrate it only for the ALE, because identical proofs work for the auxiliary model, setting D=0 instead of $D = \sqrt{\sigma }$.

#### Lemma 4.4

There exists a constant *A* depending only on η, *T* and the initial conditions such that for any $1 \le n < \left\lfloor T/\textbf{c} \right\rfloor$, on the event $\{ n < \tau _D \}$ we have

$$ Z_n \ge \textbf{c}^{\eta } \sigma ^{-(\eta - 1)} $$

almost surely.

The above bound is not sharp: the $\textbf{c}^{\eta }$ can be removed, but the proof is more complicated, and the bound in the lemma is all that we require.

#### Proof

For $|\theta | < \textbf{c}$ and a fixed *j*, let $z = e^{\sigma + i (\phi _n^j + \theta )}$, and using (4.2) we can write

$$ |(\Phi ^{\textrm{ALE}}_n)'(z)| = \left| (\Psi _{N_j}^j)'(z)\right| \left| (f^{\textrm{ALE}}_{n_j(N_j)})'(\Psi _{N_j}^j(z))\right| \left| (\Phi ^{\textrm{ALE}}_0 \circ \dots \circ \Psi _{N_j - 1}^j)'( f^{\textrm{ALE}}_{n_j(N_j)}(\Psi _{N_j}^j(z)) ) \right| . $$

Applying Lemma 3.5 to the first term in this decomposition with $z_0 = e^{i\phi _n^j}$, because $n \le T/\textbf{c}$ we have a constant lower bound L>0 on the distance between $u_t(z_0)$ and the driving function. Therefore we have from (3.8) that

4.10 $$\begin{aligned} |(\Psi _{N_j}^j)'(z)| = |(\Psi _{N_j}^j)'(e^{i\phi _n^j})| (1 + O( \textbf{c})). \end{aligned}$$

From (3.2) and (4.5) we obtain

4.11 $$\begin{aligned} |(f^{\textrm{ALE}}_{n_j(N_j)})'(\Psi _{N_j}^j(z))|&= \frac{1 + d(\textbf{c})}{|1 - e^{i\beta _\textbf{c}}|}(1 + O(\textbf{c}^{1/2})) \left| \Psi _{N_j}^j(z) - e^{i\theta ^{\textrm{ALE}}_{n_j(N_j)}} \right| \nonumber \\&= \frac{1 + d(\textbf{c})}{|1 - e^{i\beta _\textbf{c}}|} | z - e^{i\phi _n^j} | \left| (\Psi _{N_j}^j)'(e^{i\phi _n^j}) \right| (1 + O(\textbf{c}^{1/2})). \end{aligned}$$

Analysis of Loewner’s reverse equation when *z* is far from the driving measure shows that there is a constant A>0 with

$$ A^{-1} \le \left| (\Psi _{N_j}^j)'(e^{i\phi _n^j}) \right| \le A, $$

(see [1, equation (26)]) and using (4.5) and (4.8) we know

$$ |f^{\textrm{ALE}}_{n_j(N_j)}(\Psi _{N_j}^j(z))| - 1 \ge \textbf{c}^{1/2}, $$

and so by standard conformal map estimates (see [3, Chapter 4.4]),

4.12 $$\begin{aligned} \frac{\textbf{c}^{1/2}}{A} \le \left| (\Phi ^{\textrm{ALE}}_0 \circ \dots \circ \Psi _{N_j - 1}^j)'( f^{\textrm{ALE}}_{n_j(N_j)}(\Psi _{N_j}^j(z)) ) \right| \le A \textbf{c}^{-1/2}. \end{aligned}$$

Then combining (4.10), (4.11) and (4.12), we have

$$\begin{aligned} |(\Phi ^{\textrm{ALE}}_n)'(e^{\sigma + i(\phi _n^j + \theta )})|^{-\eta }&\ge A \textbf{c}^{\eta } | e^{\sigma + i\theta } - 1 |^{-\eta }. \end{aligned}$$

Thus, using the bound $|e^{\sigma + i\theta }-1|^2 = (e^{\sigma }-1)^2 + 2e^{\sigma }(1 - \cos \theta ) \le A (\sigma ^2 + \theta ^2)$ for $|\theta | < \textbf{c}$ and substituting $x = \theta /\sigma$, we get

$$\begin{aligned} Z_n&\ge A \textbf{c}^{\eta } \int _{-\textbf{c}}^{\textbf{c}} \frac{\textrm{d}\theta }{|e^{\sigma + i\theta } - 1|^{\eta }}\\&\ge A \textbf{c}^{\eta } \sigma ^{-(\eta - 1)} \int _{-\textbf{c}/\sigma }^{\textbf{c}/\sigma } \frac{\textrm{d}x}{(1 + x^2)^{\eta /2}}, \end{aligned}$$

and since $\sigma < \textbf{c}$, the integral term is bounded below by a constant. □

#### Lemma 4.5

Let $$\zeta \in \Delta $$. If $$\textbf{c}$$ is sufficiently small, then $$\log |f(\zeta )| < \textbf{c}^{1/2}$$ implies that $|f^{\prime }\left(\zeta \right)|>1$.

#### Proof

Let $e^{r + i\theta } = f(\zeta )$. We can use Proposition 3.1 to obtain

$$\begin{aligned} (f^{-1})'(w) = \frac{f^{-1}(w)}{w} \frac{w-1}{\sqrt{(w+1)^2 - 4e^{\textbf{c}}w}} \end{aligned}$$

for $$w \in \Delta \setminus (1, 1+d(\textbf{c})]$$.

Then we know that $|f^{-1}(w)| < |w|$ for any *w*, and if we write $w = e^{r + i\theta }$, then we can calculate that

$$ \left| \frac{w-1}{(w+1)^2 - 4e^c w} \right|< 1 \iff \cos \theta < \frac{e^\textbf{c}}{\cosh r}. $$

Hence $$|(f^{-1})'(w)| < 1$$, and so $|f^{\prime }\left(\zeta \right)|>1$.

Since $\cosh ^{-1}(e^{\textbf{c}}) \sim (2\textbf{c})^{1/2}$ for small $\textbf{c}$, if $\textbf{c}$ is sufficiently small and $r < \textbf{c}^{1/2}$, then the condition $\cos \theta < \frac{e^\textbf{c}}{\cosh r}$ is satisfied for any θ. □

#### Lemma 4.6

For any sufficiently large constant $A_T$, there exists a constant B>0 depending on *T* and $K_0$ such that on the event $\{n < \tau _{\textrm{coupling}}\}$, i.e. when the ALE and auxiliary models choose the same particle at each of the first *n* steps, the following is almost surely true: for any $\theta \in \mathbb {T}$, if k≤n satisfies

$$\begin{aligned} | (f^{\textrm{ALE}}_{l+1} \circ f^{\textrm{ALE}}_{l+2} \circ \dots \circ f^{\textrm{ALE}}_{n})(e^{i\theta }) - e^{i\theta ^{\textrm{ALE}}_{l}} | \ge | e^{i (\beta _\textbf{c}+ \frac{\textbf{c}^{1/2}}{A_T})} - 1 | {\text { for all }} k \le l \le n, \end{aligned}$$

and $$|e^{i\theta } - e^{i\theta ^{\textrm{ALE}}_n}| \ge |e^{i (\beta _\textbf{c}+ \frac{\textbf{c}^{1/2}}{A_T})} - 1| $$ then $$ \left| (f^{\textrm{ALE}}_{k} \circ \dots \circ f^{\textrm{ALE}}_{n})'(e^{i\theta }) \right| \le B \textbf{c}^{-1/2}. $$

#### Proof

On the event $\{n < \tau _{\textrm{coupling}}\}$, each angle $\theta ^{\textrm{ALE}}_{l}$ is within *D* of the $j_l$th tip, $\phi ^{j_l}_{l-1}$. Define the coupled angle sequence $(\bar{\theta }_l)_{l\le n}$ and corresponding particle maps $(\bar{f}_l)_{l\le n}$ inductively by $\bar{\theta }_1 = \phi ^{j_1}_0$, and $\bar{\theta }_l = \bar{\phi }^{j_l}_{l-1}$ for l>1, where $\bar{\phi }^{j_l}_{l-1}$ is the preimage of the $j_l$th tip under $\bar{\Phi }_{l-1} = \bar{f}_1 \circ \dots \circ \bar{f}_{l-1}$.

The difference between the two angle sequences is then almost surely $\sup _{l \le n} |\theta ^{\textrm{ALE}}_l - \bar{\theta }_l| < A \textbf{c}^{-1}D$, for a constant A>0. Then using the bound on $\left| \log \frac{(u^1_t)'(z)}{(u^0_t)'(z)} \right|$ from Lemma 3.5, we can establish the bound we want on the ALE process by establishing it using the angle sequence $(\bar{\theta }_l)_{l \le n}$, since $D = \sqrt{\sigma }$ is very small. We can also think of this as first considering D=0 and then perturbing the result.

Since we have fixed a T<∞, there is a positive constant *L* such that $L < \min _{i \not = j} |\bar{\phi }^i_l - \bar{\phi }^j_l|$ for all $1 \le l \le n$. Let $A_{L}$ be the corresponding constant in the bounds (3.4) and (3.5) from Lemma 3.4. For any given $\theta \in \mathbb {T}$ there can be at most one *j* such that there exists an l≤n with $|\bar{f}_l(e^{i\theta }) - \bar{\phi }^j_{l-1}| \le L e^{- A_{L} T} =: m$. If there is no such *j*, or the corresponding *l* is less than *k*, then by (3.4), $\left| (\bar{f}_{k} \circ \dots \circ \bar{f}_{n})'(e^{i\theta }) \right| \le e^{A_m T}.$

Suppose that such a *j* does exist. We will split the angle sequence $(\bar{\theta }_l)_{k \le l \le n}$ into times when particles are attached to slit *j* and times when particles are attached elsewhere.

Set $n_0 = \max \{ k \le l \le n: \bar{\theta }_l = \bar{\phi }^j_l \}$, then for i≥0 set $n_i' = \max \{ k \le l < n_i: \bar{\theta }_l \not = \bar{\phi }^j_l \}$ and $n_{i+1} = \max \{ k \le l < n_i': \bar{\theta }_l = \bar{\phi }^j_l \}$ until one of the sets is empty, and call the last well defined value $n_{p+1}$ (if $n_{p}'$ is the final well-defined value of the above maxima, then set $n_{p+1} = n_{p}'$). Define the capacities $t_0 = (n - n_0)\textbf{c}$, and for i≥0, $t_i' = (n_i - n_i')\textbf{c}$ and $t_{i+1} = (n_i' - n_{i+1})\textbf{c}$. The map $\bar{f}_{k} \circ \dots \circ \bar{f}_{n}$ is then generated by using the backwards equation (3.3) with a driving function which first is at a distance at least *L* from $\bar{\phi }^j_n$ for a period of duration $t_0 \ge 0$, then takes the constant value $\phi ^j_{n_0}$ for a period of duration $t_0' \ge \textbf{c}$, then is distance at least *L* from tip *j* again for duration $t_1 \ge \textbf{c}$, and so on, terminating after a total time $(n-k+1)\textbf{c}\le T$.

We can therefore decompose $\bar{f}_{k} \circ \dots \circ \bar{f}_{n}$ as

$$\begin{aligned} \Psi _{p+1} \circ f_{t_p', \bar{\phi }^j_{n_p'}} \circ \Psi _p \circ \dots \circ f_{t_0', \bar{\phi }^j_{n_0'}} \circ \Psi _0 \end{aligned}$$

where the Ψ maps are generated by driving functions bounded away from the *j*th slit.

Using (3.4), we have $$ |\Psi _i'( f_{t_{i-1}',\bar{\phi }^j_{n_{i-1}'}} \circ \dots \circ \Psi _0(e^{i\theta }) )| \le e^{A_{L} t_i} $$ for each *i*, and so the total contribution to $$ \left| (\bar{f}_{k} \circ \dots \circ \bar{f}_{n})'(e^{i\theta }) \right| $$ by $\Psi^{\prime }$ terms is bounded by a constant $$e^{A_{L}T}$$.

The other terms can give a larger contribution. Let $\delta _i$ be the distance in $\mathbb {R}/2\pi \mathbb {Z}$ between $\arg [(\Psi _i \circ \dots \circ \Psi _0)(e^{i\theta })]$ and $\bar{\phi }^j_{n_i}$, and similarly let $\delta _i'$ be the distance between $\arg [(f_{t_{i}',\bar{\phi }^j_{n_{i}'}} \circ \Psi _i \circ \dots \circ \Psi _0)(e^{i\theta })]$ and $\bar{\phi }^j_{n_i}$. Then using (3.2) we can compute

$$\begin{aligned} \left| f_{t_{i}',\bar{\phi }^j_{n_{i}'}}'( \Psi _i \circ \dots \circ \Psi _0)(e^{i\theta }) )\right| = e^{t_i' / 2} \frac{ |e^{i\delta _i} - 1| }{ |e^{i\delta _i'} - 1| }. \end{aligned}$$

Using (3.5), we can calculate $\frac{\delta _{i+1}}{\delta _i'} \le e^{A_{L}t_i}$, and so the total contribution from all of the $f_{t_{i}', \bar{\phi }^j_i}'$ terms is, possibly increasing the constant $A_{L}$,

$$\begin{aligned} e^{\frac{1}{2}\sum _{i=0}^{p} t_i'} \frac{ \left| e^{i\delta _0} - 1 \right| }{ \left| e^{i\delta _p'} - 1 \right| } \prod _{i=0}^{p-1} \frac{|e^{i\delta _{i+1}} - 1|}{|e^{i\delta _i'} - 1|}&\le e^{(\frac{1}{2} + A_{L}) T} \frac{2}{\textbf{c}^{1/2}/A_T}\\&= B \textbf{c}^{-1/2} \end{aligned}$$

as required. □

#### Proof of Proposition 4.2

First note that on the event $\{n < \tau _D\}$, we have

$$\mathbb {P}[ n+1 < \tau _D \,|\, (\theta _1, \dots , \theta _n) ] \le \left( 1 - \sum _j \int _{\phi ^j_n-D}^{\phi ^j_n+D} h_n(\theta ) \,\textrm{d}\theta \right) + \left( 1 - \sum _j \int _{\bar{\phi }^j_n-D}^{\bar{\phi }^j_n+D} h_n^*(\theta )\,\textrm{d}\theta \right) $$

almost surely. We will hence bound $1 - \sum _j \int _{-D}^{D} h_n( \phi ^j_n +\theta )\,\textrm{d}\theta$, and a similar bound will apply to the auxiliary term. Let $z = e^{\sigma + i\theta }$. Note that

$$\begin{aligned} 1 - \sum _j \int _{-D}^{D} h_n(\phi ^j_n + \theta ) \textrm{d}\theta&= \sum _j \int _{D< |\theta - \phi ^j_n| < \textbf{c}^2} h_n(\theta )\textrm{d}\theta + \int _{\{ |\theta - \phi ^j_n| \ge \textbf{c}^2\, \forall j \}} h_n(\theta )\textrm{d}\theta . \end{aligned}$$

If $D< |\theta - \phi _n^j| < \textbf{c}^2$ for some *j*, then we can use (4.10), (4.11) and (4.12) to establish the almost-sure upper bound

4.13 $$\begin{aligned} |(\Phi ^{\textrm{ALE}}_n)'(e^{\sigma + i\theta })|^{-\eta } \le A |e^{\sigma + i\theta } - e^{i\phi _n^j}|^{-\eta } \le A D^{-\eta }. \end{aligned}$$

Then using (4.13) and Lemma 4.4, almost surely

$$\begin{aligned} \int _{\phi _n^j + D}^{\phi _n^j + \textbf{c}^2} h_n(\theta ) \textrm{d}\theta&\le A \textbf{c}^{-\eta } D^{-(\eta - 1)} \sigma ^{\eta -1}. \end{aligned}$$

Next we suppose θ is further than $\textbf{c}^2$ from any tip $\phi _n^j$.

We will classify the point $z = e^{\sigma + i\theta }$ based on its projection $\hat{z} = e^{i\theta }$ and follow them both through their backwards evolutions, so let $z_k = (f_k \circ f_{k+1} \circ \dots \circ f_n)(z)$, and also let $\hat{z}_k = (f_k \circ f_{k+1} \circ \dots \circ f_n)(\hat{z})$. Let *L* be a lower bound on $\min _{l \le n}\min _{i \not = j}|\phi _l^i - \phi _l^j|$, let $A_{L/2}$ be the constant in Lemma 3.4 corresponding to points further than *L*/2 from 1, and let $A_T = e^{A_{L/2}T}$. Observe that there can be at most two values of *k* such that both $\hat{z}_k \in \mathbb {T}$ and $|\hat{z}_k - e^{i\theta ^{\textrm{ALE}}_{k-1}}| < |e^{i(\beta _\textbf{c}+ \frac{\textbf{c}^{1/2}}{A_T})} - 1|,$ because after the second condition is met once, applying $\left\lfloor T/\textbf{c} \right\rfloor$ maps cannot move $\hat{z}_k$ further than β away from a tip, and it will be taken off the circle the next time a slit is attached near to it. This gives us three cases depending on the number of *k*s.

**Case I:** First suppose there is no such *k*. Then by Lemma 4.6, $|(f^{\textrm{ALE}}_{1} \circ \dots \circ f^{\textrm{ALE}}_{n})'(\hat{z})| \le B\textbf{c}^{-1/2}$ almost surely, so by (3.7) from Lemma 3.5,

$$ |(f^{\textrm{ALE}}_{1} \circ \dots \circ f^{\textrm{ALE}}_{n})(z) - (f^{\textrm{ALE}}_{1} \circ \dots \circ f^{\textrm{ALE}}_{n})(\hat{z})| \le B \textbf{c}^{-1/2}\sigma . $$

This gives us $|(f^{\textrm{ALE}}_{1} \circ \dots \circ f^{\textrm{ALE}}_{n})(z)| - 1 \le B \textbf{c}^{-1/2}\sigma$, and hence by Lemma 4.5, $|(f^{\textrm{ALE}}_{1} \circ \dots \circ f^{\textrm{ALE}}_{n})'(z)| > 1.$ Let *j* be the index of the closest $\phi _0^j$ to $(f^{\textrm{ALE}}_{1} \circ \dots \circ f^{\textrm{ALE}}_{n})(\hat{z})$. We can decompose $\Phi _0$ as $f_{t_j,\phi _0^j} \circ \Psi ^j_0$, where $\Psi ^j_0$ is generated in the reverse equation by a driving function run for total time $c_0 - t^j$ which stays at least distance *L*/4 from $(u_s(\hat{z}))_{s \le c_0 - t^j}$ at all times. Then using equation (26) of [1],

$$ e^{-A c_0} \le |(\Psi _0^j)'( (f^{\textrm{ALE}}_{1} \circ \dots \circ f^{\textrm{ALE}}_{n})(\hat{z}) )| \le e^{A c_0} $$

for an appropriate constant $A = A(K_0)$. If none of the *n* particles have been attached to the initial *j*th slit, then the assumption we made on θ tells us $|(\Psi _0^j \circ f^{\textrm{ALE}}_{1} \circ \dots \circ f^{\textrm{ALE}}_{n})(\hat{z}) - e^{i\phi _0^j}| \ge \textbf{c}/ A$. If any particles *were* attached to the *j*th slit, then we must have $|(\Psi _0^j \circ f^{\textrm{ALE}}_{1} \circ \dots \circ f^{\textrm{ALE}}_{n})(\hat{z}) ) - e^{i\phi _0^j}| \ge \textbf{c}^{1/2}/A > \textbf{c}/A$, otherwise some *k* as above would exist. In either case we have

$$ |(\Psi _0^j \circ f^{\textrm{ALE}}_{1} \circ \dots \circ f^{\textrm{ALE}}_{n})(z) ) - e^{i\phi _0^j}| \ge \textbf{c}/2A, $$

and so

$$ |f_{t_j,\phi _0^j}'( (\Psi _0^j \circ f^{\textrm{ALE}}_{1} \circ \dots \circ f^{\textrm{ALE}}_{n})(z) )| \ge \textbf{c}/18A. $$

Thus, for a modified constant *A*, $|\Psi _0'(z)| \ge \textbf{c}/A$, and so $h_n(\theta ) = O( \sigma ^{\eta - 1}/\textbf{c}^{2\eta } )$.

**Case II:** If there is only one such *k*, we must have $|\hat{z}_k - e^{i\theta ^{\textrm{ALE}}_{k-1}}| \ge \frac{\textbf{c}^{1/2}}{2 A_T^2},$ since there is an l≥k with $\theta ^{\textrm{ALE}}_{l}$ attached at the same slit as $\theta ^{\textrm{ALE}}_{k-1}$ and if $\hat{z}_k$ were any closer to $e^{i\theta ^{\textrm{ALE}}_{k-1}}$ then $\hat{z}_{l+1}$ would be within $|e^{i(\beta _\textbf{c}+ \frac{\textbf{c}^{1/2}}{A_T})} - 1|$ of $e^{i\theta ^{\textrm{ALE}}_l}$.

So $|(f^{\textrm{ALE}}_{k-1})'( \hat{z}_k )| \ge \left| f'\left( \exp \left( i\frac{\textbf{c}^{1/2}}{2A_T^2}\right) \right) \right| \ge A$ for some constant A>0, then using Lemma 3.5 and Lemma 4.6

we can derive

4.14 $$\begin{aligned} |(f^{\textrm{ALE}}_{k-1})'( z_k )| \ge A^{-1}, \end{aligned}$$

since $$\sigma \ll \textbf{c}^{3/2}$$.

To compute $|(\Phi _0 \circ f^{\textrm{ALE}}_{1} \circ \dots \circ f^{\textrm{ALE}}_{k-2})' (\hat{z}_{k-1})|$, consider two cases: (a) $|\hat{z}_k - e^{i\theta ^{\textrm{ALE}}_{k-1}}| \le |e^{i(\beta - \frac{\textbf{c}^{1/2}}{A_T})} - 1|$, or (b) $|\hat{z}_k -e^{i\theta ^{\textrm{ALE}}_{k-1}}| > |e^{i(\beta - \frac{\textbf{c}^{1/2}}{A_T})} - 1|$.

In case (a), $| \hat{z}_{k-1} | - 1 \ge |f(e^{i(\beta - \frac{\textbf{c}^{1/2}}{A_T})})| - 1 \ge \frac{\textbf{c}^{1/2}}{A}$. By an identical argument to that which established (4.12),

$$ |(\Phi _0 \circ f^{\textrm{ALE}}_{1} \circ \dots \circ f^{\textrm{ALE}}_{k-2})'( \hat{z}_{k-1} )| \ge \frac{\textbf{c}^{1/2}}{A}, $$

and again this easily extends to

4.15 $$\begin{aligned} |(\Phi _0 \circ f^{\textrm{ALE}}_{1} \circ \dots \circ f^{\textrm{ALE}}_{k-2})'( z_{k-1} )| \ge \frac{\textbf{c}^{1/2}}{A}, \end{aligned}$$

since $\sigma \ll \textbf{c}^{3/2}$. Combining (4.15) with (4.14) and Lemma 4.5, we have

$$\begin{aligned} |(\Phi ^{\textrm{ALE}}_n)'(z)| \ge \frac{\textbf{c}}{A}, \end{aligned}$$

and so $$h_n(\theta ) = O( \sigma ^{\eta -1}/\textbf{c}^{2\eta } )$$.

In case (b), we have

$$ \min _{\pm } | \hat{z}_k - e^{i(\theta ^{\textrm{ALE}}_{k-1} \pm \beta _\textbf{c})} | < \frac{2 \textbf{c}^{1/2}}{A_T}, $$

and so

$$ \min _{\pm } | z_k - e^{i(\theta ^{\textrm{ALE}}_{k-1} \pm \beta _\textbf{c})} | < \frac{3 \textbf{c}^{1/2}}{A_T}. $$

Without loss of generality the minimum is achieved in both cases by $e^{i(\theta ^{\textrm{ALE}}_{k-1} + \beta _\textbf{c})}$. Let $\delta = |z_k - e^{i(\theta ^{\textrm{ALE}}_{k-1} + \beta _\textbf{c})}|$, then by Corollary 3.2,

4.16 $$\begin{aligned} | (f^{\textrm{ALE}}_{k-1})'(z_k) | \ge A^{-1} \frac{ \textbf{c}^{1/4} }{ \delta ^{1/2} } \end{aligned}$$

and Lemma 3.3 tells us that $| z_{k-1} - e^{i\theta ^{\textrm{ALE}}_{k-1}} | \asymp \textbf{c}^{1/4} \delta ^{1/2}$. If the attachment point $\theta ^{\textrm{ALE}}_{k-1}$ is on the *j*th slit, then it is within *D* of the preimage $\phi ^j_{k-2}$ of $\theta ^{\textrm{ALE}}_{l}$ under $f^{\textrm{ALE}}_{l+1} \circ \dots \circ f^{\textrm{ALE}}_{k-2}$, where l<k-1 was the previous time a particle was attached at the *j*th slit (without loss of generality such an *l* exists, as we can decompose the initial condition to have a slit of capacity $\textbf{c}$ at the top of a longer slit in position *j*).

By (4.11), the size of $|(f^{\textrm{ALE}}_{l})'(z_{l+1})|$ depends on $|z_{l+1} - e^{i\theta ^{\textrm{ALE}}_l}|$, which, similarly to (3.5), satisfies

$$ e^{-AT} \le \frac{ |z_{l+1} - e^{i\theta ^{\textrm{ALE}}_l}| }{ | z_{k-1} - e^{i\phi _{k-2}^j} | } \le e^{AT}. $$

Then since

$$\begin{aligned} | z_{k-1} - e^{i\theta ^{\textrm{ALE}}_{k-1}} |&\le | z_{k-1} - e^{i\phi _{k-2}^j} | + | e^{i\phi _{k-2}^j} - e^{i\theta ^{\textrm{ALE}}_{k-1}} |\\&\le | z_{k-1} - e^{i\phi _{k-2}^j} | + 2D, \end{aligned}$$

we have, for some constant *A*,

$$ A^{-1} \textbf{c}^{1/4} \delta ^{1/2} \le | z_{k-1} - e^{i\phi _{k-2}^j} | + 2D \le e^{AT} |z_{l+1} - e^{i\theta ^{\textrm{ALE}}_l}| + 2D, $$

and so

$$\begin{aligned} |z_{l+1}-e^{i\theta ^{\textrm{ALE}}_l}| \ge \sigma \vee \left( \frac{\textbf{c}^{1/4}\delta ^{1/2}}{A} - 2D\right) . \end{aligned}$$

Then

$$ |(f^{\textrm{ALE}}_{l})'(z_{l+1})| \ge \frac{1}{A} \textbf{c}^{-1/2} |z_{l+1} - e^{i\theta ^{\textrm{ALE}}_l}|, $$

and so combining this with Lemma 4.5 and (4.16),

4.17 $$\begin{aligned} |(f^{\textrm{ALE}}_{l} \circ \dots \circ f^{\textrm{ALE}}_{n})'(z)|&\ge \frac{ \sigma \vee ( A^{-1}\textbf{c}^{1/4}\delta ^{1/2} - 2D )}{\delta ^{1/2}}. \end{aligned}$$

Since $|z_{k-1} - e^{i\phi _{k-2}^j}| \ge \sigma$, (4.17) is bounded below by $A^{-1} \textbf{c}^{-1/4} \frac{\sigma }{9D^2}$. Then since $| z_l | - 1 \ge \textbf{c}^{1/2}$, we have an overall lower bound on $|\Phi _n'(z)|$ of

$$ A^{-1} \textbf{c}^{1/2} \frac{ \sigma \vee ( A^{-1}\textbf{c}^{1/4}\delta ^{1/2} - 2D )}{\delta ^{1/2}}. $$

Note that δ is proportional to $|z - (f_n \circ \dots \circ f_{k+1})(e^{i(\theta ^{\textrm{ALE}}_{k-1}+\beta _\textbf{c})})|$, so we can integrate our bound on $| \Phi _n'(z) |^{-\eta }$ from σ to $\textbf{c}^2$ to get $\int _{\{ {\text {one}}\, k\, {\text {exists}} \}} h_n(\theta )\textrm{d}\theta \le A \sigma ^{-1} D^{\eta + 2} \textbf{c}^{-\frac{5\eta }{4} - \frac{1}{2}}$.

**Case III:** If there are two values of *k*, $k_2 < k_1$, we can find a bound of the same order using the same argument as above, replacing *k* by $k_1$ and *l* by $k_2$. □

### Auxiliary to multinomial model

Next we will define the *multinomial model*, in which the probability of attaching a particle at each tip depends on the second derivative of the relevant map. This essentially corresponds to taking $\sigma \rightarrow 0$ in the auxiliary model.

We explained the significance of the second derivative of the map for the Laplacian path model in Section 2.4, so the multinomial model can be viewed as a halfway point between the ALE and LPM.

#### Definition 4.2.1

Begin with the same initial condition as the other models, $\Phi ^{\textrm{multi}}_0 = \Phi _0$. Let the preimages of the *k* tips under $\Phi ^{\textrm{multi}}_n$ be $\phi _n^j$ for j=1,2,⋯,k. Choose $\theta _{n+1}$ from $\{ \phi _n^1, \dots , \phi _n^k \}$, with probabilities

$$ \mathbb {P}( \theta _{n+1} = \phi _n^j ) = \frac{ | (\Phi ^{\textrm{multi}}_n)''(e^{i\phi _n^j}) |^{-\eta } }{ Z_n }, $$

where $Z_n = \sum _j | (\Phi ^{\textrm{multi}}_n)''(e^{i\phi _n^j}) |^{-\eta }$. Define inductively the maps

$$\begin{aligned} \Phi ^{\textrm{multi}}_{n+1} = \Phi ^{\textrm{multi}}_n \circ f_{\theta _{n+1}}. \end{aligned}$$

If they choose the same tips, the auxiliary model (re-rotated to fix the basepoints) and the multinomial model coincide exactly. It is therefore fairly simple to prove a coupling exists as long as the probabilities assigned to the tips are close:

#### Proposition 4.7

Let $(\theta ^{*}_l)_{l \le n}$ be the angle sequence for the auxiliary model, without the rotation used in Section 4.1, and let $(\theta _l)_{l \le n}$ be the angle sequence for the multinomial model with the same initial conditions. Define the stopping time $\tau _{\not =} = \inf \{ l : \theta ^{*}_l \not = \theta _l \}$. On the event $\{ l < \tau _{\not =} \wedge n \}$, the conditional distributions of $\theta ^{*}_{l+1}$ and $\theta _{l+1}$ are almost surely supported on the same set $\{ \phi _l^1, \dots , \phi _l^k \}$, and when σ is as in Theorem 4.1 and $D = \sqrt{\sigma }$ we almost surely have

$$\begin{aligned} \max _{1 \le j \le k} | \mathbb {P}( \theta ^{*}_{l+1} = \phi _l^j \,|\, (\theta _k)_{k \le l} ) - \mathbb {P}( \theta _{l+1} = \phi _l^j \,|\, (\theta _k)_{k \le l} ) | \le A \textbf{c}^{-1}D \end{aligned}$$

for a deterministic constant *A* depending on *T* and η and $\Phi _0$.

#### Corollary 4.8

We can construct the coupling of $(\theta ^{*}_l)_{l \le n}$ and $(\theta _l)_{l \le n}$ in such a way that $\mathbb {P}(\tau _{\not =} < \left\lfloor T/\textbf{c} \right\rfloor ) \le AT \textbf{c}^{-2}D$.

The proof of Proposition 4.7 is relatively simple: since $(\Phi ^{*}_n)'(e^{i\phi _l^j}) = 0$, the value of $\mathbb {P}( \theta ^{*}_{l+1} = \phi _l^j )$ is asymptotically proportional to $|(\Phi ^{*}_n)''(e^{i\phi _l^j})|^{-\eta }$, and hence approximates $\mathbb {P}( \theta _{l+1} = \phi _l^j )$.

#### Proof of Proposition 4.7

We need to show $\int _{-D}^{D} |(\Phi ^{*}_l)'(e^{\sigma + i(\phi _l^j+\theta )})|^{-\eta } \,\textrm{d}\theta$ is proportional to $|(\Phi ^{*}_l)''(e^{i\phi _l^j})|^{-\eta }$. For |θ|<D, let γ be the line segment in $\overline{\Delta }$ from $e^{i\phi _l^j}$ to $e^{\sigma + i(\phi _l^j+\theta )}$. Then by the fundamental theorem of calculus,

$$\begin{aligned} (\Phi ^{*}_l)'(e^{\sigma + i(\phi _l^j+\theta )})&= (e^{\sigma + i\theta } - 1) e^{i\phi _l^j} (\Phi ^{*}_l)''(e^{i\phi _l^j}) + \int _\gamma (e^{\sigma + i(\phi _l^j + \theta )} - z) (\Phi ^{*}_l)^{(3)}(z) \,\textrm{d}z, \end{aligned}$$

and so

4.18 $$\begin{aligned} \left| \log \frac{ |(\Phi ^{*}_l)'(e^{\sigma + i(\phi _l^j+\theta )})| }{ |e^{\sigma + i\theta } - 1| \big |(\Phi ^{*}_l)''(e^{i\phi _l^j})\big | }\right|&\le \frac{ |e^{\sigma + i\theta } - 1| \sup _{z \in \gamma } | (\Phi ^{*}_l)^{(3)}(z) | }{ |(\Phi ^{*}_l)''(e^{i\phi _l^j})| }. \end{aligned}$$

We can decompose $\Phi ^{*}_l$ as $\Phi ^{*}_{k-1} \circ f_k \circ \Psi _k$, where $1 \le k \le l$ is the last time a particle was attached to slit *j* (if no particle has been attached we can regard the top part of the initial slit as a particle of capacity $\textbf{c}$, so without loss of generality we can assume at least one particle has been added to each slit). For *u* satisfying the backwards equation (3.3) with driving function ξ on [0, *T*], equation (26) of [1] gives an expression for the spatial derivative

$$ u_t'(z) = \exp \left( t - \int _0^t \frac{2 \,\textrm{d}s}{(u_s(z)e^{-i\xi _{T-s}}-1)^2} \right) . $$

Using this expression and its higher spatial derivatives, we find that for a constant *A* depending only on η, *T* and the initial conditions, we have bounds $A^{-1} \le |\Psi _k'(z)| \le A$, $|\Psi _k''(z)| \le A$, $|\Psi _k^{(3)}(z)| \le A$ for all $z \in \gamma$. Then by the chain rule, and as $f_k'(\Psi _k(e^{i\phi _l^j})) = 0$,

$$\begin{aligned} |(\Phi ^{*}_l)''(e^{i\phi _l^j})|&= \left| \Psi _k''(e^{i\phi _l^j}) (\Phi ^{*}_{k-1} \circ f_k)'(\Psi _k(e^{i\phi _l^j})) + (\Psi _k'(e^{i\phi _l^j}))^2 (\Phi ^{*}_{k-1} \circ f_k)''(\Psi _k(e^{i\phi _l^j})) \right| \\&\ge A^{-2} \left| (\Phi ^{*}_{k-1} \circ f_k)''(\Psi _k(e^{i\phi _l^j})) \right| \\&= A^{-2} |f_k''(\Psi _k(e^{i\phi _l^j}))| \times |(\Phi ^{*}_{k-1})'(f_k(\Psi _k(e^{i\phi _l^j})))|. \end{aligned}$$

Since $$\Psi _k(e^{i\phi _l^j}) = e^{i\theta ^{*}_k}$$,

4.19 $$\begin{aligned} |f_k''(\Psi _k(e^{i\phi _l^j}))| = f''(1) = \frac{1 + d(\textbf{c})}{2\sqrt{1-e^{-\textbf{c}}}} \ge \frac{\textbf{c}^{-1/2}}{4}, \end{aligned}$$

and $|f_k(\Psi _k(e^{i\phi _l^j}))| - 1 \ge \textbf{c}^{1/2}$, so $|(\Phi ^{*}_{k-1})'(f_k(\Psi _k(e^{i\phi _l^j})))| \ge \frac{\textbf{c}^{1/2}}{A}$. Hence we have a constant lower bound on $|(\Phi ^{*}_l)''(e^{i\phi _l^j})|$, and only need an upper bound on $\sup _{z \in \gamma } | (\Phi ^{*}_l)^{(3)}(z) |$. By repeated application of the chain rule with the same decomposition of $\Phi ^{*}_l$, similarly to the above we find

$$ | (\Phi ^{*}_l)^{(3)}(z) | \le A \textbf{c}^{-1} $$

for all $z \in \gamma$. Hence the right-hand side of (4.18) is almost surely bounded by $A \textbf{c}^{-1} D$, and so $\mathbb {P}( \theta ^{*}_{l+1} = \phi _l^j ) = e^{O(D/\textbf{c})} \mathbb {P}( \theta _{l+1} = \phi _l^j ).$ □

## Multinomial to Laplacian path model

The step from the multinomial model to the LPM is the most difficult and technically involved part of the proof of Theorem 2.1, so deserves its own section. We must track the movement of the preimages of the tips in the two models, which are governed by Loewner’s equation close to the driving function where it is singular, and the second derivative of the maps $\Phi _n^{\textrm{multi}}$ and $\Phi _{t}^{\textrm{LPM}}$, which are governed by the second spatial derivative of the same singular PDE.

As the Laplacian path model is generated by a driving *measure* rather than a function, we will first specify what we mean by convergence of driving measures.

### Definition 5.0.1

Given a metric space (*X*, *d*), and two Borel measures $\mu _1, \mu _2$ on *X*, the bounded Wasserstein distance $d_{\textrm{BW}}(\mu _1, \mu _2)$ is defined

$$ d_{\textrm{BW}}(\mu _1, \mu _2) = \sup _{\varphi \in \mathcal {H}} \left| \int _{X} \varphi \,\textrm{d}\mu _1 - \int _{X} \varphi \,\textrm{d}\mu _2 \right| , $$

where $$\mathcal {H} = \{ \varphi \in C(X): \Vert \varphi \Vert _{\textrm{Lip}} + \Vert \varphi \Vert _{\infty } \le 1 \}$$, for

$$ \Vert \varphi \Vert _{\textrm{Lip}} = \sup _{x \not = y} \frac{|\varphi (x) - \varphi (y)|}{d(x,y)}, \quad \Vert \varphi \Vert _{\infty } = \sup _{x} |\varphi (x)|. $$

### Proposition 5.1

Let (*X*, *d*) be a separable metric space, then $d_{\textrm{BW}}$ metrises weak convergence of finite measures on *X*, i.e. if μ, $\mu _n$ for $n \in \mathbb {N}$ are finite measures on *X*, then $\mu _n \Rightarrow \mu$ if and only if $d_{\textrm{BW}}(\mu _n, \mu ) \rightarrow 0$ as $n \rightarrow \infty$.

### Proof

See Theorem 11.3.3 of [14]. □

### Remark 19

Weak convergence $\mu _n \Rightarrow \mu$ of measures on *X* can also be implied by convergence $\int _X \varphi \,\textrm{d}\mu _n \rightarrow \int _X \varphi \,\textrm{d}\mu$ for bounded continuous functions φ. We use the smaller space of test functions $\varphi \in \mathcal {H}$ in this section because it is substantially easier to prove convergence of the integrals for our two measures in this case, as we make use of the bound on $\Vert \varphi \Vert _{\textrm{Lip}}$ in the proof of Corollary 5.3, and this still suffices to imply weak convergence of the measures.

### Remark 20

We can view each driving measure $(\mu _t)_{t \in [0,T]}$ as a single probability measure μ on the cylinder $S = \mathbb {T}\times [0,T]$ given by $\mu _t \otimes m_{[0,T]}$ where $m_{[0,T]}$ is normalised Lebesgue measure on [0, *T*]. Then by Proposition 1 of [15], weak convergence of these measures on the cylinder implies convergence of the corresponding clusters in the Carathéodory topology.

We will use Proposition 5.1 to deduce convergence of the cylinder measures corresponding to the multinomial model and LPM by showing that the preimages of the tips are close and the second derivatives are close at these preimages.

### Definition 5.0.2

Let

$$x_j(t) := e^{i\phi ^j_t} \quad {\text { and }} \quad \bar{x}_j(t) := e^{i\bar{\phi }^j_t}$$

where $\phi ^j_t$ and $\bar{\phi }^j_t$ are the preimages defined in the statement of Theorem 2.1.

For t∈[0,T] let

5.1 $$\begin{aligned} I^j_t := 1\{ \xi _t = x_j(t) \} = 1\{ \theta _{\left\lfloor t/\textbf{c} \right\rfloor +1} = \phi ^j_{\left\lfloor t/\textbf{c} \right\rfloor \textbf{c}} \} = I^j_{n\textbf{c}}, \end{aligned}$$

for $t \in [n\textbf{c},(n+1)\textbf{c})$, and note that $\mathbb {E}( I^l_{n\textbf{c}} | \theta _1, \dots , \theta _n) = p^l_{n\textbf{c}}$.

For all t≤T define

$$ \delta _\textrm{x}(t) := \sup _{s \le t} \sum _{j=1}^{k} | x_j(s) - \bar{x}_j(s) |, $$

and

$$ \delta _{\textrm{p}}(t) := \sup _{s \le t} \sum _{j=1}^{k} \left| \Phi _s''(x_j(s)) - \bar{\Phi }_s''(\bar{x}_j(s)) \right| . $$

Note that for some constant *A*, $|p_t^j - \bar{p}_t^j| \le A \delta _{\textrm{p}}(t)$ for all $j \in \{1, \dots , k\}$ and all t≤T.

Finally, let

$$ \delta _{\textrm{total}}(t) := \delta _{\textrm{x}}(t) + \delta _{\textrm{p}}(t). $$

### Theorem 5.2

Under the same assumptions as in Theorem 2.1, there exists a universal constant *R* and a constant $A = A(T,L,k,K_0,\eta )$ such that

$$\begin{aligned} \mathbb {P}\left[ \delta _\textrm{total}(T) \le A\textbf{c}^{1/2R} \right] \rightarrow 1 \end{aligned}$$

as $$\textbf{c}\rightarrow 0$$.

### Corollary 5.3

If $\mu _t = \delta _{\theta _{\left\lfloor t/\textbf{c} \right\rfloor +1}}$ for the multinomial model and $\bar{\mu }_t = \sum _{j=1}^{k} \bar{p}^j_t \delta _{\bar{\phi }^j_t}$ for the Laplacian path model, then

$$\begin{aligned} d_{\textrm{BW}}(\mu _t \otimes m_{[0,T]}, \bar{\mu }_t \otimes m_{[0,T]}) \rightarrow 0 \end{aligned}$$

in probability as $\textbf{c}\rightarrow 0$. Hence $\mu _t \otimes m_{[0,T]}$ converges in distribution, as a random element of the space of measures on $S = \mathbb {T}\times [0,T]$, to $\bar{\mu }_t \otimes m_{[0,T]}$.

To derive Corollary 5.3 from Theorem 5.2, we also use Proposition 5.4, which we will prove in the following section.

### Proof of Corollary 5.3

Let $\varphi \in \mathcal {H}$. For simplicity assume that *T* is an integer multiple of $\textbf{c}$. Then

$$\begin{aligned} \int _0^T&\int _{\mathbb {T}} \varphi \,\textrm{d}\mu _t\,\textrm{d}t - \int _0^T \int _{\mathbb {T}} \varphi \,\textrm{d}\bar{\mu }_t\,\textrm{d}t = \sum _{j=1}^k \int _0^T \left( I^j_t \varphi (x_j(t)) - \bar{p}^j_t \varphi ( \bar{x}_j(t) ) \right) \textrm{d}t\\&= \sum _{j=1}^k \int _0^T \left( (I^j_t - p^j_t) \varphi (x_j(t)) + (p^j_t - \bar{p}^j_t) \varphi (x_j(t)) + \bar{p}^j_t (\varphi (x_j(t)) - \varphi (\bar{x}_j(t))) \right) \textrm{d}t. \end{aligned}$$

The latter two terms are small with high probability using Theorem 5.2, so we only need to bound the “martingale” terms $\int _0^T (I^j_t - p^j_t)\varphi (x_j(t))\,\textrm{d}t$ for each *j*. We can write each term as

$$\begin{aligned} \sum _{n=0}^{\frac{T}{\textbf{c}}-1} \left[ (I^j_{n\textbf{c}}-p^j_{n\textbf{c}}) \int _{n\textbf{c}}^{(n+1)\textbf{c}} \varphi (x_j(t)) \,\textrm{d}t + \int _{n\textbf{c}}^{(n+1)\textbf{c}} (p^j_{n\textbf{c}} - p^j_t)\varphi (x_j(t)) \,\textrm{d}t \right] . \end{aligned}$$

Since $|p^j_{n\textbf{c}} - p^j_t| \le A \textbf{c}$ by Proposition 5.4, we have $|\sum _{n=0}^{\frac{T}{\textbf{c}}-1} \int _{n\textbf{c}}^{(n+1)\textbf{c}} (p^j_{n\textbf{c}} - p^j_t) \varphi (x_j(t))\,\textrm{d}t| \le AT\textbf{c}$. For the remaining term, we would like to simply compute second moments and so show the term is small, but need some way of doing this uniformly in φ. Choose some large *N*, and for simplicity assume it is a factor of $T/\textbf{c}$. Then write

$$\begin{aligned}&\sum _{m=1}^{N} \sum _{n=\frac{(m-1)T}{\textbf{c}N}}^{\frac{mT}{\textbf{c}N}-1} (I^j_{n\textbf{c}} - p^j_{n\textbf{c}})\int _{n\textbf{c}}^{(n+1)\textbf{c}}\varphi (x_j(t))\,\textrm{d}t\\&= \sum _{m=1}^{N} \textbf{c}\varphi (x_j(mT/N)) \sum _{n=\frac{(m-1)T}{\textbf{c}N}-1}^{\frac{mT}{\textbf{c}N}} (I^j_{n\textbf{c}} - p^j_{n\textbf{c}}) + \delta , \end{aligned}$$

where $|\delta | \le \frac{AT^2}{N}$ using $\Vert \varphi \Vert _{\textrm{Lip}}\le 1$. Now we will be able to find a bound independent of φ, as

$$\begin{aligned} \mathbb {E}\left( \textbf{c}\sum _{n=\frac{(m-1)T}{\textbf{c}N}}^{\frac{mT}{\textbf{c}N}-1} (I^j_{n\textbf{c}} - p^j_{n\textbf{c}}) \right) ^2&\le 4\textbf{c}^2 \frac{T}{\textbf{c}N} = \frac{4T\textbf{c}}{N} \end{aligned}$$

by conditional independence of the increments $I^j_{n\textbf{c}} - p^j_{n\textbf{c}}$. Define the event

$$\begin{aligned} E_m = \left\{ \left| \textbf{c}\sum _{n=\frac{(m-1)T}{\textbf{c}N}-1}^{\frac{mT}{\textbf{c}N}} (I^j_{n\textbf{c}} - p^j_{n\textbf{c}}) \right| \ge \frac{2\sqrt{T}\textbf{c}^{1/4}}{N} \right\} , \end{aligned}$$

then $\mathbb {P}(E_m) \le \textbf{c}^{1/2}N$, and so

$$\begin{aligned} \mathbb {P}\left( \sup _{\varphi \in \mathcal {H}} \left| \sum _{m=1}^{N} \textbf{c}\varphi (x_j(mT/N)) \sum _{n=\frac{(m-1)T}{\textbf{c}N}-1}^{\frac{mT}{\textbf{c}N}} (I^j_{n\textbf{c}} - p^j_{n\textbf{c}}) \right| \ge 2\sqrt{T} \textbf{c}^{1/4} \right)&\le \mathbb {P}\left( \bigcup _{m=1}^N E_m \right) \\&\le \textbf{c}^{1/2}N^2. \end{aligned}$$

We chose *N* arbitrarily, so if $1 \ll N \ll \textbf{c}^{-1/4}$, we have

$$\begin{aligned} \sup _{\varphi \in \mathcal {H}} \left| \int _0^T \int _{\mathbb {T}} \varphi \,\textrm{d}\mu _t \, \textrm{d}t - \int _0^T \int _{\mathbb {T}} \varphi \,\textrm{d}\bar{\mu }_t \, \textrm{d}t \right| \rightarrow 0 \end{aligned}$$

in probability, as required. □

Our proof of Theorem 5.2 has two parts: first we show in Proposition 5.4 that $t \mapsto (x_j(t), \bar{x}_j(t), p^j_t, \bar{p}^j_t)$ is Lipschitz on [0, *T*], which will allow us to deduce global bounds on $|x_j(t) - \bar{x}_j(t)|$ and $|p^j_t - \bar{p}^j_t|$ by showing that they are close at a sufficiently dense set of times.

Then in Proposition 5.5 and Proposition 5.6, we find bounds on $\delta _{\textrm{p}}(t)$ and $\delta _{\textrm{x}}(t)$ for t∈[0,T] which we can turn into a bound on $\delta _{\textrm{total}}(T)$ using a version of Grönwall’s inequality.

### Lipschitz bound on the locations and probabilities

#### Proposition 5.4

There exists a constant $A = A(k, K_0, T, \eta )$ such that, almost surely, for $0 \le t, s \le T$,

$$\begin{aligned} | x_j(t) - x_j(s) | \le A |t-s| \quad {\text { and }}\quad | p_t^j - p_s^j | \le A |t-s|, \end{aligned}$$

for all j=1,⋯,k in the multinomial model. For the Laplacian path model, for all *j* and for $0 \le t,s \le T$, we also have

$$\begin{aligned} | \bar{x}_j(t) - \bar{x}_j(s) | \le A |t-s| \quad {\text {and}}\quad | \bar{p}_t^j - \bar{p}_s^j | \le A |t-s|. \end{aligned}$$

#### Proof

Equation (2.7) of [2] gives a useful expression for the evolution of $x_j(t)$ over time:

5.2 $$\begin{aligned} \frac{\textrm{d}}{\textrm{d}t} x_j(t)&= \left\{ \begin{matrix} 0 &  {\text {if}}\, \xi _t = x_j(t),\\ - x_j(t) \frac{x_j(t) + x_l(t)}{x_j(t) - x_l(t)} &  {\text {if}}\, \xi _t = x_l(t)\, {\text {for}}\, l \not = j \end{matrix}\right. , \end{aligned}$$

and so as the denominator is bounded below by $L = L(k,K_0,T,\eta )$, we have

$$\begin{aligned} |x_j(t) - x_j(s)| \le \frac{2}{L} | t-s |. \end{aligned}$$

Controlling how $\Phi _t''(x_j(t))$ changes over time will involve a spatial Taylor expansion. Therefore we need to control the third derivative, so first we show that the third derivative at $x_j(t)$ takes a particularly convenient form if $\xi _{t-} = x_j(t)$. If $f_{s,x}(z)$ attaches a particle of capacity s>0 at $x \in \mathbb {T}$, then we can calculate from (3.2) that $f_{s,1}''(1) = \frac{1 + d(s)}{2\sqrt{1-e^{-s}}}$ and $f_{s,1}^{(3)}(1) = \frac{-3(1 + d(s))}{2\sqrt{1-e^{-s}}} = -3f_{s,1}''(1)$, therefore $f_{s,x}^{(3)}(x) = -\frac{3}{x}f_{s,x}''(x)$. Suppose $\Phi _t$ can be written as $\Phi _{t-s} \circ f_{s,x_j(t)}$ for some $s \in (0,\textbf{c}]$, then in this case we have $x_j(t-s) = x_j(t)$ and $\Phi _{t-s}'(x_j(t)) = 0$. Using the chain rule to calculate $(\Phi _{t-s} \circ f_{s,x_j(t)})''(x_j(t))$ and $(\Phi _{t-s} \circ f_{s,x_j(t)})^{(3)}(x_j(t))$, we also obtain $\Phi _t^{(3)}(x_j(t)) = -\frac{3 \Phi _t''(x_j(t))}{x_j(t)}$.

Suppose $t,t+\delta \in [m\textbf{c}, (m+1)\textbf{c}]$ with δ>0. We will show that $|\Phi _{t+\delta }''(x_j(t+\delta )) - \Phi _{t}''(x_j(t))| = O(\delta )$, which is sufficient to show that $t \mapsto \Phi _t''(x_j(t))$ is Lipschitz on [0, *T*]. There are two cases: either (a) $\xi _s = x_j(s)$ for all $s \in [m\textbf{c}, (m+1)\textbf{c})$ or (b) $\xi _s = x_l(s)$ for some l≠j over the same interval.

In case (a), $\Phi _{t+\delta } = \Phi _t \circ f_{\delta ,x_j(t)}$. Although we do not necessarily have $\Phi _{t}^{(3)}(x_j(t)) = -\frac{3 \Phi _{t}''(x_j(t))}{x_j(t)}$, we *do* have $\Phi _{t+\delta ^2}^{(3)}(x_j(t)) = -\frac{3 \Phi _{t+\delta ^2}''(x_j(t))}{x_j(t)}$, so let $t' = t + \delta ^2$ and $\delta ' = \delta - \delta ^2$, then write $\Phi _{t+\delta } = \Phi _{t'} \circ f_{\delta ', x_j(t)}$. To simplify the expressions write $x = x_j(t)$. Using the chain rule and the fact that $\Phi _{t'}'(x) = 0$, we obtain

5.3 $$\begin{aligned} \Phi _{t+\delta }''(x)&= \Phi _{t'}'(f_{\delta ',x}(x)) f_{\delta ',x}''(x) + 0\nonumber \\&= \Phi _{t'}'(f_{\delta ',x}(x)) \frac{1}{x} \frac{1 + d(\delta ')}{2\sqrt{1-e^{-\delta '}}}. \end{aligned}$$

Since $f_{\delta ',x}(x) - x = x d(\delta ')$, by Taylor’s theorem,

$$\begin{aligned} \Phi _{t'}'(f_{\delta ',x}(x))&= x d(\delta ')\Phi _{t'}''(x) + \frac{x^2 d(\delta ')^2}{2}\Phi _{t'}^{(3)}(x) + O(d(\delta ')^3)\\&= x d(\delta ') \left( 1 - \frac{3}{2}d(\delta ')\right) \Phi _{t'}''(x) + O(d(\delta ')^3). \end{aligned}$$

Then substituting this back into (5.3), we get

$$\begin{aligned} \Phi _{t+\delta }''(x)&= \frac{d(\delta ')(1+d(\delta '))}{2\sqrt{1-e^{-\delta '}}} \left( 1 - \frac{3}{2}d(\delta ')\right) \Phi _{t'}''(x) + O(d(\delta ')^2). \end{aligned}$$

Since $d(\delta ') = 2e^{\delta '}(1 + \sqrt{1 - e^{-\delta '}}) - 2 = 2\sqrt{\delta '} + O(\delta ')$, we can calculate $\frac{d(\delta ')(1+d(\delta '))}{2\sqrt{1-e^{-\delta '}}} = 1 + 3\sqrt{\delta '} + O(\delta ')$, and $1 - \frac{3}{2}d(\delta ') = 1 - 3\sqrt{\delta '} + O(\delta ')$, so

$$\begin{aligned} \Phi _{t+\delta }''(x)&= \Phi _{t'}''(x) + O(\delta '). \end{aligned}$$

Then writing $\Phi _{t'} = \Phi _t \circ f_{\delta ^2,x}$ and expanding with the chain rule and Taylor’s theorem as above, we do not have as much cancellation and get the slightly cruder expression

$$\begin{aligned} \Phi _{t'}''(x) = \frac{d(\delta ^2)(1+\delta ^2)}{2\sqrt{1-e^{-\delta ^2}}} \Phi _{t}''(x) + O(d(\delta ^2)), \end{aligned}$$

but this is good enough. It gives us $\Phi _{t'}''(x_j(t)) = \Phi _t''(x_j(t)) + O(\delta )$, and so

$$ \Phi _{t+\delta }''(x_j(t)) - \Phi _t''(x_j(t)) = O(\delta ). $$

In case (b), by the chain rule we have, for $s \in [t,\delta +t)$,

$$\begin{aligned} \frac{\textrm{d}}{\textrm{d}s} \Phi _s''(x_j(s))&= \left. \frac{\partial }{\partial s} \Phi _s''(z) \right| _{z=x_j(s)} + \Phi _s^{(3)}(x_j(s)) \frac{\textrm{d}}{\textrm{d}s} x_j(s). \end{aligned}$$

Differentiating (2.1) twice with respect to *z*, the first term above is

$$\begin{aligned} \left. \frac{\partial }{\partial s} \Phi _s''(z) \right| _{z=x_j(s)}&= \Phi _s^{(3)}(x_j(s)) x_j(s) \frac{x_j(s) + \xi _s}{x_j(s) - \xi _s} + \Phi _s''(x_j(s)) \left( 1 - \frac{2\xi _s^2}{(x_j(s) - \xi _s)^2} \right) \\&= -\Phi _s^{(3)}(x_j(s)) \frac{\textrm{d}}{\textrm{d}s} x_j(s) + \Phi _s''(x_j(s)) \left( 1 - \frac{2\xi _s^2}{(x_j(s) - \xi _s)^2} \right) \end{aligned}$$

by (5.2). Therefore

$$\begin{aligned} \frac{\textrm{d}}{\textrm{d}s} \Phi _s''(x_j(s))&= \Phi _s''(x_j(s)) \left( 1 - \frac{2\xi _s^2}{(x_j(s) - \xi _s)^2} \right) , \end{aligned}$$

which is bounded.

We have now shown that $t \mapsto \Phi _t''(x_j(t))$ is Lipschitz on [0, *T*], but to conclude that $t \mapsto p^j_t$ is Lipschitz we need $|\Phi _t''(x_j(t))|$ to be bounded below. This follows from the same argument used in the proof of Proposition 4.7. □

### Grönwall-type bound on the total error

We will prove the global bound in Theorem 5.2 using a singular version of Grönwall’s inequality from [16] applied to $\sum _{j=1}^{k} |x_j(t)- \bar{x}_j(t)|$ and $\sum _{j=1}^{k} |p^j_t - \bar{p}^j_t|$.

#### Proposition 5.5

Under the same conditions as Theorem 5.2, there exist constants $A = A(T,k,K_0,\eta ) < \infty$ such that

$$\begin{aligned} \mathbb {P}\left[ \delta _{\textrm{x}}(t) \le A\int _0^t \delta _{\textrm{total}}(s)\,\textrm{d}s + 3 \textbf{c}^{1/4} {\text { for all }} t \in [0,T] \right] \rightarrow 1 \end{aligned}$$

as $$\textbf{c}\rightarrow 0$$.

#### Proposition 5.6

There exist constants $A = A(T,k,K_0,\eta ) < \infty$ and R>0 such that

$$ \mathbb {P}\left[ \delta _{\textrm{p}}(t) \le A\int _0^t \frac{\delta _{\textrm{total}}(s)}{(t-s)^{51/100}}\textrm{d}s + A \textbf{c}^{1/2R} {\text { for all }} t \in [0,T] \right] \rightarrow 1 $$

as $$\textbf{c}\rightarrow 0$$.

We have stated these separately because Proposition 5.5 is much simpler to prove, while Proposition 5.6 will be divided into several steps.

#### Remark 21

In the arguments below we apply Loewner’s equation to points on the boundary of our domain, although in (2.2) we said that it applies to points in the *open* exterior disc. We could replace the points on the boundary with extremely close points in Δ and then take a limit to resolve this formally, and working on the boundary directly can be seen as a shorthand for this procedure.

#### Proof of Proposition 5.5

Throughout the proof, *A* represents a constant which may change from line to line, but all occurrences have a common upper bound depending only on *T*, *L*, *k*, $K_0$ and η.

Equation (2.7) in [2] shows that the movement of the preimages $\bar{\phi }^j_t$ in the Laplacian path model is determined by

5.4 $$\begin{aligned} \frac{\textrm{d}}{\textrm{d}t} \bar{x}_j(t)&= -\sum _{l \not = j} \bar{p}^j_t \bar{x}_j(t) \frac{\bar{x}_j(t) + \bar{x}_l(t)}{\bar{x}_j(t) - \bar{x}_l(t)} \end{aligned}$$

and similarly for the multinomial model,

5.5 $$\begin{aligned} \frac{\textrm{d}}{\textrm{d}t} x_j(t)&= \left\{ \begin{matrix} 0 &  {\text {if}}\, \xi _t = x_j(t),\\ - x_j(t) \frac{x_j(t) + x_l(t)}{x_j(t) - x_l(t)} &  {\text {if}}\, \xi _t = x_l(t)\, {\text {for}}\, l \not = j \end{matrix}\right. . \end{aligned}$$

Write

$$\begin{aligned} \lambda ^{j,l}_t = \left\{ \begin{matrix} 0 &  {\text {if }} j=l \\ -x_j(t) \frac{x_j(t)+x_l(t)}{x_j(t)-x_l(t)} &  {\text {otherwise}} \end{matrix} \right. , \end{aligned}$$

and likewise

$$\begin{aligned} \bar{\lambda }^{j,l}_t = \left\{ \begin{matrix} 0 &  {\text {if }} j=l \\ - \bar{x}_j(t) \frac{\bar{x}_j(t) + \bar{x}_l(t)}{\bar{x}_j(t) - \bar{x}_l(t)} &  {\text {otherwise.}} \end{matrix} \right. \end{aligned}$$

Therefore for t∈[0,T] we can write, integrating (5.4) and (5.5) with respect to time,

$$\begin{aligned} \bar{x}_j(t)&= \bar{x}_j(0) + \sum _{l \not = j} \int _0^t \bar{\lambda }^{j,l}_s \bar{p}^l_s \, \textrm{d}s,\\ x_j(t)&= x_j(0) + \sum _{l\not =j} \int _{0}^{t} \lambda ^{j,l}_s I^l_s \,\textrm{d}s, \end{aligned}$$

where $I^l_s$ is as defined in (5.1). To bound the size of $\bar{x}_j(t) - x_j(t)$, we can expand the difference of the integrands into three terms

$$\begin{aligned} \lambda ^{j,l}_s I^l_s - \bar{\lambda }^{j,l}_s \bar{p}^l_s&= (\lambda ^{j,l}_s - \bar{\lambda }^{j,l}_s) I^l_s + \bar{\lambda }^{j,l}_s (I^l_s - p^l_s) + \bar{\lambda }^{j,l}_s (p^l_s - \bar{p}^l_s). \end{aligned}$$

As the denominators in the above definitions of $\lambda ^{j,l}_t$ and $\bar{\lambda }^{j,l}_t$ are bounded below, we can linearise $|\lambda ^{j,l}_s - \bar{\lambda }^{j,l}_s| \le A\left( |x_j(s) - \bar{x}_j(s)| + |x_l(s) - \bar{x}_l(s)|\right) \le A \delta _{\textrm{x}}(s)$, and $\sum _{l\not =j}|\bar{\lambda }^{j,l}_s| |p_s^l - \bar{p}_s^l| \le A \delta _{\textrm{p}}(s)$, and so by the triangle inequality

5.6 $$\begin{aligned} | x_j(t) - \bar{x}_j(t) |&\le A \int _0^t \delta _{\textrm{total}}(s)\,\textrm{d}s + \sum _{l\not =j} \left| \int _0^t \bar{\lambda }^{j,l}_s (I^l_s - p^l_s)\,\textrm{d}s \right| . \end{aligned}$$

To finish our proof we need to bound the martingale-type terms $\int _0^t \bar{\lambda }^{j,l}_s (I^l_s - p^l_s)\,\textrm{d}s$. Note that for $n \in \mathbb {N}\cup \{0\}$ with $n \le T/\textbf{c}$, by Proposition 5.4,

$$\begin{aligned} \int _{n\textbf{c}}^{(n+1)\textbf{c}\wedge t} \bar{\lambda }_{s}^{j,l} (I^l_s - p^l_s) \,\textrm{d}s&= \int _{n\textbf{c}}^{(n+1)\textbf{c}\wedge t} \bar{\lambda }_{s}^{j,l} (I^l_{n\textbf{c}} - p^l_{n\textbf{c}}) \,\textrm{d}s + \int _{n\textbf{c}}^{(n+1)\textbf{c}\wedge t} \bar{\lambda }_{s}^{j,l} (p^l_{n\textbf{c}} - p^l_s)\, \textrm{d}s\\&= (I^l_{n\textbf{c}} - p^l_{n\textbf{c}})\int _{n\textbf{c}}^{(n+1)\textbf{c}\wedge t} \bar{\lambda }_{s}^{j,l} \,\textrm{d}s + O(\textbf{c}^2). \end{aligned}$$

We will bound each term in the sum on the right-hand side of (5.6), so fix some l≠k. Let $X_n:= (I^l_{n\textbf{c}} - p^l_{n\textbf{c}})\int _{n\textbf{c}}^{(n+1)\textbf{c}} \bar{\lambda }_{s}^{j,l} \,\textrm{d}s$ and note that $X_n$ is a bounded martingale increment, $\mathbb {E}[X_n | \theta _1, \dots , \theta _n] = 0$, so define the martingale $M_n:= \sum _{i=0}^n X_n$. Note that

$$\begin{aligned} \sup _{t \le T} \left| \int _0^t \bar{\lambda }^{j,l}_s (I^l_s - p^l_s)\,\textrm{d}s - M_{\left\lfloor t/\textbf{c} \right\rfloor }\right| = O(\textbf{c}), \end{aligned}$$

so if we can bound $\max _{n \le T/\textbf{c}} |M_n|$ then our result follows.

The increments $X_n$ are bounded by $A\textbf{c}$ for a constant *A* depending only on $T, K_0$ and η, so $\mathbb {E}[ |M_{\left\lfloor T/\textbf{c} \right\rfloor }|^2 ] \le A^2 \textbf{c}$. Therefore, by Doob’s submartingale inequality,

$$\begin{aligned} \mathbb {P}\left[ \max _{n \le T/\textbf{c}} |M_n| \ge \frac{1}{k}\textbf{c}^{1/4} \right] \le \frac{A \textbf{c}}{\textbf{c}^{1/2}}, \end{aligned}$$

for a constant *A*. So for sufficiently small $\textbf{c}$, on an event of probability at least $1 - A \textbf{c}^{1/2}$ we have $\sum _{l\not =j} \left| \int _0^t \bar{\lambda }^{j,l}_s (I^l_s - p^l_s)\,\textrm{d}s \right| \le 3\textbf{c}^{1/4}$ and the result follows. □

In the proof of Proposition 5.6 we will use some analytic techniques from [2, Section 4.2]. First we require a few further definitions, mostly following the notation of [2].

#### Definition 5.2.1

Fix t∈[0,T], and for s≤t define the *transition maps*

5.7 $$\begin{aligned} h_s := \Phi _s^{-1} \circ \Phi _t, \quad \bar{h}_s := \bar{\Phi }_s^{-1} \circ \bar{\Phi }_t. \end{aligned}$$

**Fig. 5:**
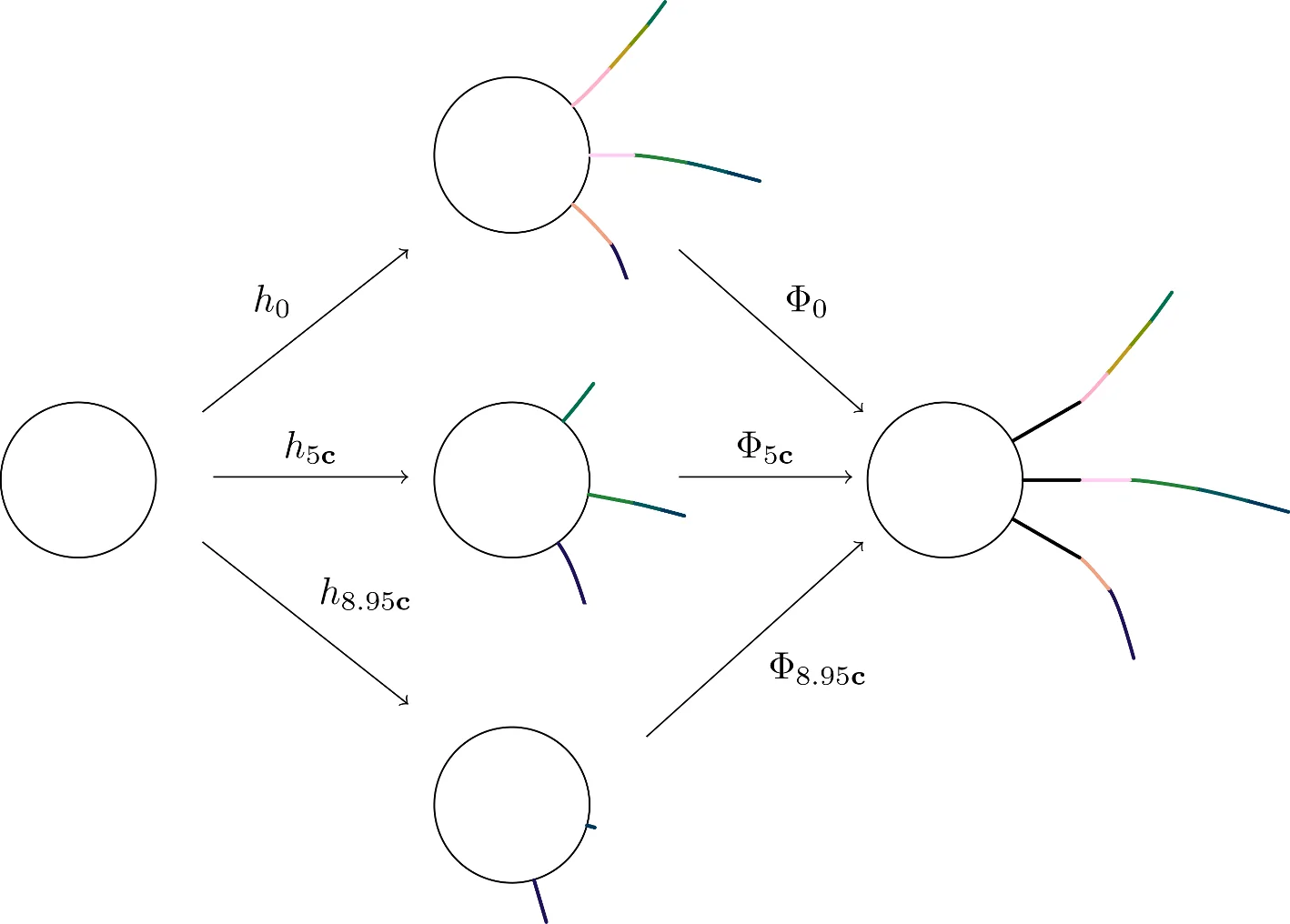
Several transition maps as in Definition 5.2.1 for a multinomial cluster with ten particles attached to an initial three slits. Notice that none of the transition maps attach the initial three slits to the circle, and that the image of $\mathbb {T}$ under the transition map $h_t$ is not monotone in *t*: although the capacity is always decreasing, the slits are also moving around

Several transition maps for one multinomial model cluster are shown in Figure 5. Note that if $t = n \textbf{c}$ for some $n \in \mathbb {N}$, then $h_0 = f_1 \circ \dots \circ f_n$ and $h_{k\textbf{c}} = f_{k+1} \circ \dots \circ f_n$ for $k \in \mathbb {N}$ with k<n.

For s∈[0,t] and $j \in \{1, \dots , k\}$, define

5.8 $$\begin{aligned} w_j(s) := h_s( x_j(t) ), \quad \bar{w}_j(s) = \bar{h}_s( \bar{x}_j(t) ). \end{aligned}$$

Also define

5.9 $$\begin{aligned} \kappa _j(s) := h_s''(x_j(t)), \quad \bar{\kappa }_j(s) := \bar{h}_s''(\bar{x}_j(t)). \end{aligned}$$

#### Remark 22

The transition map $h_s$ is related to the backward equation (3.3). If started from the trivial initial condition $\Phi _0 = \textrm{Id}_\Delta$, then for t=T, $h_s = u_{T-s}$ for all s∈[0,T], where *u* is the solution to (3.3). We have, however, used the notation $h_s$ rather than $u_{t-s}$ to avoid confusion, because our $\Phi _0$ is non-trivial and the endpoint may be any t∈(0,T], not just the final *T*.

#### Definition 5.2.2

Let $j \in \{1,\dots ,k\}$ and $n \le \left\lfloor T/\textbf{c} \right\rfloor$. Let p>0 be such that $p^j_{m\textbf{c}} \ge p$ for all $m \le \left\lfloor T/\textbf{c} \right\rfloor$ almost surely (we know that such a deterministic constant *p* must exist). Define the event

$$\begin{aligned} H_j(n) := \left\{ I^j_{n\textbf{c}} = 1 {\text { and, for all }} m \le n, \sum _{l=m}^{n} I^j_{l\textbf{c}} \ge \frac{1}{2}p(n-m+1) \right\} . \end{aligned}$$

#### Remark 23

For the rest of Section 5 we will repeatedly condition on $H_j(n)$, so the reader may wish to make a note of Definition 5.2.2 or mark its location.

#### Remark 24

Since $(I^j_{l\textbf{c}})_{l\le \left\lfloor T/\textbf{c} \right\rfloor }$ dominates a collection of iid Bernoulli(*p*) random variables, $\mathbb {P}(H_j(n))$ is bounded below by a constant depending only on *T*, η and $K_0$.

We will prove Proposition 5.6 via several intermediate results, all stated here before we prove them.

#### Lemma 5.7

Let $j \in \{1, \dots , k\}$, and let $t = n\textbf{c}$ for some $n \le \left\lfloor T/\textbf{c} \right\rfloor$. Let the event $H_j(n)$ be as in Definition 5.2.2. There exists a constant *A* depending on $K_0, T$ and η such that, conditional on $H_j(n)$ we almost surely have

5.10 $$\begin{aligned} \left| \Phi _{t}''(x_j(t)) - \bar{\Phi }_{t}''(\bar{x}_j(t)) \right|&\le A |(w_j(0) - x_j(0)) - (\bar{w}_j(0) - \bar{x}_j(0))|\nonumber \\&\phantom {\le }+ A\left| \kappa _j(0)(w_j(0) - x_j(0)) - \bar{\kappa }_j(0)(\bar{w}_j(0) - \bar{x}_j(0))\right| . \end{aligned}$$

#### Lemma 5.8

Let *j*, *n* and *t* be as above. Conditional on $H_j(n)$,

5.11 $$\begin{aligned} \log [\kappa _j(0)( w_j(0) - x_j(0) )]&= 2t - \sum _{l=1}^{k} \int _0^t I^l_s Q_s^{j,l} \end{aligned}$$

almost surely, where

5.12 $$\begin{aligned} Q^{j,j}_s&:= \frac{-3x_j(s)}{w_j(s) - x_j(s)}, \end{aligned}$$

and

5.13 $$\begin{aligned} Q^{j,l}_s&:= \frac{2x_l(s)^2}{(w_j(s)-x_l(s))^2} + \frac{2x_l(s)^2}{(w_j(s)-x_l(s))(x_j(s)-x_l(s))} \end{aligned}$$

for l≠j. Similarly,

5.14 $$\begin{aligned} \log [\bar{\kappa }_j(0)( \bar{w}_j(0) - \bar{x}_j(0) )]&= 2t - \sum _{l=1}^{k}\int _0^t \bar{p}^l_s \bar{Q}^{j,l}_s \,\textrm{d}s, \end{aligned}$$

where the quantities $\bar{Q}_s^{j,l}$ are the LPM equivalents of (5.12) and (5.13). Then we can bound the final term in (5.10),

5.15 $$\begin{aligned} \left| \kappa _j(0)(w_j(0) - x_j(0)) - \bar{\kappa }_j(0)(\bar{w}_j(0) - \bar{x}_j(0))\right| \le A \sum _{l=1}^{k} \left| \int _0^t (I_s^l Q_s^{j,l} - \bar{p}^l_s \bar{Q}^{j,l}_s) \,\textrm{d}s \right| . \end{aligned}$$

We can linearise the integrand in the line above to bound it by something involving $|(w_j(s)-x_j(s)) - (\bar{w}_j(s) - \bar{x}_j(s))|$:

#### Proposition 5.9

For some constant R≥9 there exists an event $E_{\textrm{p}}^t$ with $\mathbb {P}[E_{\textrm{p}}^t \,|\, H_j(n) ] \ge 1 - A \textbf{c}^{4/R}\log (T/\textbf{c})$ such that on $E_{\textrm{p}}^t \cap H_j(n)$, (5.10) is almost surely bounded by a constant multiple of

$$\begin{aligned} \textbf{c}^{\frac{1}{2} - \frac{2}{R}} + \int _0^t \frac{\delta _{\textrm{total}}(s)}{\sqrt{t-s}}\,\textrm{d}s + |(w_j(0) - x_j(0)) - (\bar{w}_j(0) - \bar{x}_j(0))|\\ + \int _0^t \frac{|(w_j(s) - x_j(s)) - (\bar{w}_j(s) - \bar{x}_j(s))|}{t-s}\,\textrm{d}s. \end{aligned}$$

Now if we can control $|(w_j(s)-x_j(s)) - (\bar{w}_j(s) - \bar{x}_j(s))|$, Proposition 5.6 will follow.

#### Lemma 5.10

Let *j*, *n* and *t* be as above. For s∈[0,t] let

$$\begin{aligned} u_s := (w_j(s) - x_j(s)) - (\bar{w}_j(s) - \bar{x}_j(s)). \end{aligned}$$

Then, conditional on $H_j(n)$, *u* almost surely satisfies the integral inequality

$$\begin{aligned} |u_s| \le X(s) + A \int _s^t \frac{X(r)}{t-r}\,\textrm{d}r, \end{aligned}$$

for all s∈[0,t], where

$$\begin{aligned} X(s) := A\int _s^t \frac{\delta _{\textrm{total}}(r)}{\sqrt{t-r}}\,\textrm{d}r + \sum _{l=1}^k \left| \int _s^t C^l_r (I^l_r - p^l_r)\,\textrm{d}r \right| , \end{aligned}$$

and the $C^l_r$ are deterministic functions (depending on *t*) such that $|C^l_r| \le A(t-r)^{-1/2}$ for all r∈[0,t).

#### Corollary 5.11

For *j*, *n* and *t* as above, and for the *u* defined in Lemma 5.10, conditional on $H_j(n)$, on an event of conditional probability at least $1 - A\textbf{c}^{4/R}\log (1/\textbf{c})$, where *R* is some positive universal constant, we have

$$\begin{aligned} |u_0|&\le A\int _0^t \frac{\delta _{\textrm{total}}(s)}{(t-s)^{51/100}}\textrm{d}s + A\textbf{c}^{1/R}\log (1/\textbf{c}), \end{aligned}$$

and

$$\begin{aligned} \int _0^t \frac{|u_s|}{t-s}\,\textrm{d}s&\le A\int _{0}^{t} \frac{\delta _{\textrm{total}}(s)}{(t-s)^{51/100}} + A\textbf{c}^{1/R}(\log (1/\textbf{c}))^2. \end{aligned}$$

Now we will prove the above five results in turn.

#### Proof of Lemma 5.7

By the chain rule we have $\Phi _t''(x_j(t)) = \kappa _j(0)\Phi _0'(w_j(0))$, and so

$$\begin{aligned} \Phi _{t}''(x_j(t))&= \frac{\Phi _0'(w_j(0))}{w_j(0) - x_j(0)} \times \kappa (0) (w_j(0) - x_j(0)). \end{aligned}$$

We will repeatedly make use of expansions of the form $ab - \bar{a} \bar{b} = (a - \bar{a})b + \bar{a} (b - \bar{b})$ to linearise the difference of complex expressions. Applying this to the above decomposition of $\Phi _t''(x_j(t))$ and its LPM equivalent, we have

5.16 $$\begin{aligned} \left| \Phi _{t}''(x_j(t)) - \bar{\Phi }_{t}''(\bar{x}_j(t)) \right|&\le \left| \frac{\Phi _0'(w_j(0))}{w_j(0)-x_j(0)} - \frac{\Phi _0'(\bar{w}_j(0))}{\bar{w}_j(0)-\bar{x}_j(0)} \right| |\kappa (0) (w_j(0) - x_j(0))|\nonumber \\&+ \left| \frac{\Phi _0'(\bar{w}_j(0))}{\bar{w}_j(0)-\bar{x}_j(0)} \right| \left| \kappa _j(0)(w_j(0)-x_j(0)) - \bar{\kappa }_j(0)(\bar{w}_j(0)-\bar{x}_j(0)) \right| . \end{aligned}$$

We claim that both $\left| \frac{\Phi _0'(\bar{w}_j(0))}{\bar{w}_j(0)-\bar{x}_j(0)}\right|$ and $|\kappa _j(0)(w_j(0) - x_j(0))|$ are bounded. The former is bounded by $\sup _{\zeta \in [x_j(0),\bar{w}_j(0)]} | \Phi _0''(\zeta ) |$. The Schwarz–Christoffel representation [6, equation (1)] tells us $\Phi _0'(z) = \frac{\Phi _0(z)}{z} S(z)$, where $S(z):= \frac{\prod _{l=1}^k (z - x_j(0))}{\sqrt{\prod _{l=1}^{k} (z - b_{+}^l)(z - b_{-}^l) }}$, and $b_{\pm }^j$ are the two preimages of the base of the *j*th slit. We can differentiate this to verify that $|\Phi _0''(\zeta )|$ is bounded when ζ is not close to any of the $b_{\pm }^l$, which is the case for $\zeta \in [x_j(0), \bar{w}_j(0)]$. Next note that $\sum _{l=1}^{k} |Q_{s}^{j,l}| = O((t-s)^{-1/2})$, which is integrable, and so we can establish a finite upper bound and positive lower bound on $|\kappa _j(0)(w_j(0) - x_j(0))|$ using (5.11) (the proof of which does not rely on (5.10)).

To simplify the first term on the right-hand side of (5.16), since $\Phi _0'(x_j(0)) = 0$ we can use the fundamental theorem of calculus to write

$$\begin{aligned} \Phi _0'(w_j(0)) = (w_j(0)-x_j(0))\int _0^1 \Phi _0''( \alpha w_j(0) + (1-\alpha )x_j(0) )\,\textrm{d}\alpha , \end{aligned}$$

and similarly for $\Phi _0'(\bar{w}_j(0))$. Then, using the Schwarz–Christoffel representation again to bound $|\Phi _0^{(3)}(\zeta )|$ and noting that $\bar{x}_j(0) = x_j(0)$, we can bound the first term on the RHS of (5.16) by

$$\begin{aligned} A\int _0^1 \left| \Phi _0''(p w_j(0) + (1-p)x_j(0)) - \Phi _0''(p \bar{w}_j(0) + (1-p)\bar{x}_j(0)) \right| \textrm{d}p&\le A |w_j(0) - \bar{w}_j(0)|, \end{aligned}$$

which is equal to $A|(w_j(0) - x_j(0)) - (\bar{w}_j(0) - \bar{x}_j(0))|$, so (5.10) holds. □

#### Proof of Lemma 5.8

As *t* is fixed, we will denote $\frac{\partial }{\partial s}h_s(z)$ by $\dot{h}_s(z)$, etc. The main effect of conditioning on $H_j(n)$ is that $h_s'(x_j(t)) = 0$ for all s∈[0,t) (the same is always true for the LPM). Where it is unambiguous we will write $x_j = x_j(s)$, $w_j = w_j(s)$, etc. We will prove (5.11), and omit the proof of (5.14) as it is very similar.

For any *z*, $h_s(z)$ is continuous in s∈[0,t] and the inverse Loewner equation describes the evolution of $h_s$ for 0<s<t:

5.17 $$\begin{aligned} \frac{\partial }{\partial s} h_s(z)&= - h_s(z) \sum _{l=1}^{k} I_s^l \frac{h_s(z) + x_l(s)}{h_s(z) - x_l(s)}. \end{aligned}$$

Using this to track $z = x_j(t)$, we have for s∈(0,t) that

5.18 $$\begin{aligned} \dot{w}_j(s) = - \sum _{l=1}^{k} I^l_s w_j\frac{w_j + x_l}{w_j - x_l} = - \sum _{l=1}^{k} I^l_s \left( w_j + 2x_l + \frac{2x_l^2}{w_j - x_l} \right) . \end{aligned}$$

Note that $$w_j(0) = \Phi _0^{-1}( \Phi _t(x_j(t)) )$$

and $w_j(t) = x_j(t)$. Since $h_s'(x_j(t)) = 0$ for s<t, differentiating the right-hand side of (5.17) twice with respect to *z* and evaluating at $x_j(t)$ gives us

$$\begin{aligned} \frac{\dot{\kappa }(s)}{\kappa (s)}&= - \sum _{l=1}^{k} I^l_s \left( 1 - \frac{2x_l(s)^2}{(w_j(s) - x_l(s))^2} \right) . \end{aligned}$$

As s↑t, since we have conditioned on $H_j(n)$ we have $|w_j(s) - x_j(s)|^{-2} \approx \frac{1}{t-s}$ as s↑t. Therefore the l=j term above has a non-integrable singularity, which we would like to eliminate. Subtracting (5.5) from (5.18) gives

5.19 $$\begin{aligned} \dot{w}_j - \dot{x}_j&= -\sum _{l} I^l_s \left( w_j + 2x_l + \frac{2x_l^2}{w_j - x_l} \right) +\sum _{l \not = j} I^l_s x_j\frac{x_j+x_l}{x_j-x_l}\nonumber \\&= - I^j_s \left( w_j + 2x_j + \frac{2x_j^2}{w_j-x_j} \right) + \sum _{l\not =j} I^l_s \left( \frac{2x_l^2}{(x_j-x_l)(w_j-x_l)} - 1 \right) (w_j - x_j), \end{aligned}$$

and so

$$\begin{aligned} \frac{\dot{w}_j - \dot{x}_j}{w_j - x_j}&= -I^j_s \left( \frac{w_j + 2x_j}{w_j - x_j} + \frac{2x_j^2}{(w_j-x_j)^2} \right) + \sum _{l\not =j} I^l_s \left( \frac{2x_l^2}{(x_j-x_l)(w_j-x_l)} - 1\right) . \end{aligned}$$

Hence

$$\begin{aligned} \frac{\dot{\kappa }}{\kappa } + \frac{\dot{w}_j - \dot{x}_j}{w_j-x_j}&= -I^j_s \left( 2 + \frac{3 x_j}{w_j-x_j} \right) + \sum _{l\not =j} I^l_s \left( \frac{2x_l^2}{(w_j-x_l)^2} + \frac{2x_l^2}{(w_j-x_l)(x_j-x_l)} - 2 \right) \\&= -2 -I^j_s \frac{3 x_j}{w_j-x_j} + \sum _{l\not =j} I^l_s \left( \frac{2x_l^2}{(w_j-x_l)^2} + \frac{2x_l^2}{(w_j-x_l)(x_j-x_l)} \right) \\&= -2 + \sum _{l=1}^{k} I_s^l Q_s^{j,l}. \end{aligned}$$

There is still a singularity in the $Q_{s}^{j,j}$ term as s↑t, but it is integrable. Therefore

$$\begin{aligned} \log \frac{\lim _{s \uparrow t} \kappa _j(s)( w_j(s) - x_j(s) )}{\kappa (0)( w_j(0) - x_j(0) ) }&= -2 t + \sum _{l=1}^{k}\int _0^{t} I^l_s Q^{j,l}_s \,\textrm{d}s. \end{aligned}$$

We claim that the numerator on the left-hand side is equal to 1. For $s \in (t-\textbf{c},t)$, $h_s = f^{\theta _n, t-s}$ as in Definition 2.2.1, and $x_j(s) = x_j(t) = e^{i\theta _n}$. Then

$$w_j(s) - x_j(s) = f^{\theta _n,t-s}(e^{i\theta _n}) - e^{i\theta _n} = e^{i\theta _n} d(t-s),$$

and using (4.19) we have

$$\kappa _j(s) = e^{-i\theta _n} (f^{t-s})''(1) = e^{-i\theta _n} \frac{1 + d(t-s)}{2\sqrt{1 - e^{-(t-s)}}}.$$

Since $d(t-s) \sim 2\sqrt{t-s}$ as s↑t, we get $\kappa _j(s)(w_j(s) - x_j(s)) = (1 + d(t-s))\frac{d(t-s)}{2\sqrt{1 - e^{-(t-s)}}} \rightarrow 1$ as s↑t, so (5.11) follows.

We have already shown in the proof of Lemma 5.7 that $A^{-1} \le |\kappa _j(0)(w_j(0) - x_j(0))| \le A$, and using (5.14) we obtain similar upper and lower bounds on $|\bar{\kappa }_j(0)(\bar{w}_j(0) - \bar{x}_j(0))|$. Therefore we can use the mean value theorem to bound the left-hand side of (5.15) by a constant multiple of $\Big |\log [\kappa _j(0)(w_j(0) - x_j(0))] - \log [\bar{\kappa }_j(0)(\bar{w}_j(0) - \bar{x}_j(0))]\Big |$ and obtain (5.15). □

#### Proof of Proposition 5.9

We only need to bound the right-hand side of (5.15). We decompose the integrands into three terms,

$$\begin{aligned} I^l_s Q^{j,l}_s - \bar{p}^l_s \bar{Q}^{j,l}_s&= (I^l_s - p^l_s) \bar{Q}^{j,l}_s + (p^l_s - \bar{p}^l_s) \bar{Q}^{j,l}_s + I^l_s (Q^{j,l}_s - \bar{Q}^{j,l}_s), \end{aligned}$$

so the *l*th term on the right-hand side of (5.15) is bounded by a constant multiple of

5.20 $$\begin{aligned} \left| \int _0^{t} (I^l_s - p^l_s) \bar{Q}^{j,l}_s \,\textrm{d}s \right| + \int _0^{t} |p^l_s - \bar{p}^l_s| |\bar{Q}^{j,l}_s| \,\textrm{d}s + \int _0^{t} |Q^{j,l}_s - \bar{Q}^{j,l}_s| \,\textrm{d}s. \end{aligned}$$

As in the proof of Proposition 5.5, for $s \in [m\textbf{c},(m+1)\textbf{c})$ we can write $I^l_s - p^l_s = I^l_{m\textbf{c}} - p^l_{m\textbf{c}} + O(\textbf{c})$, and so the first term in (5.20) can be written

$$ \left| \sum _{m=0}^{n-1} (I^l_{m\textbf{c}} - p^l_{m\textbf{c}}) \int _{m\textbf{c}}^{(m+1)\textbf{c}} \bar{Q}^{j,l}_s\,\textrm{d}s \right| + O(\textbf{c}). $$

Since $\bar{Q}^{j,l}_s$ is deterministic and $(I_{m\textbf{c}}^l - p_{m\textbf{c}}^l)_{m \ge 0}$ are martingale increments with respect to $(\theta _m)_{m\ge 0}$, this sum resembles a martingale, so we could try to control it by bounding its second moment. However, by conditioning on $H_j(n)$ we can no longer use adaptedness. Fortunately, for all $n \le \left\lfloor T/\textbf{c} \right\rfloor$, $\mathbb {P}(H_j(n)) \ge A^{-1}$ and so for each $l \in \{1,\dots ,k\}$,

$$\begin{aligned}&\mathbb {E}\left[ \left| \sum _{m=0}^{n-1} (I^l_{m\textbf{c}} - p^l_{m\textbf{c}}) \int _{m\textbf{c}}^{(m+1)\textbf{c}} \bar{Q}^{j,l}_s\,\textrm{d}s \right| ^2 \,\Bigg |\, H_j(n) \right] \\&\quad \le A \mathbb {E}\left[ \left| \sum _{m=0}^{n-1} (I^l_{m\textbf{c}} - p^l_{m\textbf{c}}) \int _{m\textbf{c}}^{(m+1)\textbf{c}} \bar{Q}^{j,l}_s\,\textrm{d}s \right| ^2\right] \\&\quad \le 2A\mathbb {E}\left[ \sum _{m=0}^{n-1} \left( \int _{m\textbf{c}}^{(m+1)\textbf{c}} |\bar{Q}^{j,l}_s| \,\textrm{d}s \right) ^2 \right] , \end{aligned}$$

where the final inequality comes from the martingale property and the fact that $|I^l_{m\textbf{c}}-p^l_{m\textbf{c}}| \le 1$ (the factor of 2 is because we bound both the real and imaginary parts of $\bar{Q}^{j,l}_s$ by $|\bar{Q}^{j,l}_s|$). Then $|\bar{Q}^{j,l}_s| \le A/\sqrt{t-s}$, and so

$$\begin{aligned} \sum _{m=0}^{n-1} \mathbb {E}\left( \int _{m\textbf{c}}^{(m+1)\textbf{c}} |\bar{Q}^{j,l}_s| \,\textrm{d}s\right) ^2 \le A \textbf{c}\sum _{m=0}^{n-1}( \sqrt{m+1} - \sqrt{m})^2 \le A\textbf{c}\sum _{m=0}^{n - 1} \frac{A}{m+1} \le A \textbf{c}\log \frac{t}{\textbf{c}}. \end{aligned}$$

Define the event

5.21 $$\begin{aligned} E_{\textrm{p}}^t := \left\{ \sum _{l=1}^{k}\left| \int _0^t (I_s^l - p_s^l) \bar{Q}^{j,l}_s \,\textrm{d}s \right| \le \textbf{c}^{\frac{1}{2} - \frac{2}{R}} \right\} , \end{aligned}$$

then by Markov’s inequality, $\mathbb {P}(E_{\textrm{p}}^t) \ge 1 - A \textbf{c}^{4/R}\log (T/\textbf{c})$ for sufficiently small $\textbf{c}$.

For the second term in (5.20), we use the bound $|\bar{Q}^{j,l}_s| \le A /\sqrt{t-s}$ to get

5.22 $$\begin{aligned} \sum _{l=1}^{k} \int _0^t |p_s^l - \bar{p}_s^l| |\bar{Q}_s^{j,l}| \le A \int _0^t \frac{\delta _{\textrm{p}}(s)}{\sqrt{t-s}}. \end{aligned}$$

For the final term with l≠j, the denominators in (5.13) stay away from 0 so we can linearise

$$\begin{aligned} |Q^{j,l}_s - \bar{Q}^{j,l}_s|&\le A\left( |(w_j(s) - x_j(s)) - (\bar{w}_j(s) - \bar{x}_j(s))| + |x_j(s) - \bar{x}_j(s)| + |x_l(s) - \bar{x}_l(s)| \right) \\&\le A |(w_j(s) - x_j(s)) - (\bar{w}_j(s) - \bar{x}_j(s))| + A \delta _{\textrm{x}}(s). \end{aligned}$$

The l=j term is linearised similarly,

$$\begin{aligned} |Q^{j,j}_s - \bar{Q}^{j,j}_s|&\le \frac{3|x_j(s)-\bar{x}_j(s)|}{|\bar{w}_j(s) - \bar{x}_j(s)|} + \frac{|(w_j(s) - x_j(s)) - (\bar{w}_j(s) - \bar{x}_j(s))|}{|w_j(s) - x_j(s)| |\bar{w}_j(s) - \bar{x}_j(s)|}\\&\le A\frac{\delta _{\textrm{x}}(s)}{\sqrt{t-s}} + A \frac{|(w_j(s) - x_j(s)) - (\bar{w}_j(s) - \bar{x}_j(s))|}{t - s}, \end{aligned}$$

which absorbs the l≠j terms. Note that we have used $|w_j(s) - x_j(s)| \ge A^{-1}\sqrt{t-s}$ for all s≤t, which follows from the displayed part of Definition 5.2.2. Combined with (5.22), on the event $E_{\textrm{p}}^t \cap H_j(n)$ this gives our claimed bound. □

#### Proof of Lemma 5.10

Since the *s*-derivative of $u_s$ is singular as s↑t, we will carefully analyse and control the singularity. To simplify the notation we will write $w_j = w_j(s)$, etc. Subtract (5.19) from its LPM equivalent to obtain

5.23 $$\begin{aligned} \dot{u}_s =&-((w_j - x_j) - (\bar{w}_j - \bar{x}_j))\nonumber \\&+ \sum _{l\not =j}\left( -I^l_s \frac{2x_l^2}{(x_j-x_l)(w_j-x_l)} (w_j - x_j) +\bar{p}^l_s \frac{2 \bar{x}_l^2}{(\bar{x}_j-\bar{x}_l)(\bar{w}_j-\bar{x}_l)}(\bar{w}_j - \bar{x}_j) \right) \nonumber \\&-I^j_s \left( 3x_j + \frac{2x_j^2}{w_j - x_j} \right) +\bar{p}^j_s \left( 3\bar{x}_j + \frac{2\bar{x}_j^2}{\bar{w}_j - \bar{x}_j}\right) . \end{aligned}$$

We will linearise all these terms to write (5.23) in the form $\dot{u}_s = \gamma (s)u_s + \alpha (s)$ for suitable functions γ and α. The first line on the right-hand side is clearly $-u_s$. The *l*th summand on the second line is a difference of the form $\bar{a} \bar{b} \bar{c} - abc$, so can be linearised as $(\bar{a} - a)\bar{b} \bar{c} +a(\bar{b} - b)\bar{c} + ab(\bar{c} - c)$, giving

$$\begin{aligned} (\bar{p}^l_s - I^l_s) \frac{2\bar{x}_l^2(\bar{w}_j - \bar{x}_j)}{(\bar{x}_j-\bar{x}_l)(\bar{w}_j-\bar{x}_l)}&+ I^l_s \left( \frac{2x_l^2}{(x_j-x_l)(w_j-x_l)} - \frac{2 \bar{x}_l^2}{(\bar{x}_j-\bar{x}_l)(\bar{w}_j-\bar{x}_l)} \right) (\bar{w}_j - \bar{x}_j)\\&+ I^l_s \frac{2 \bar{x}_l^2}{(\bar{x}_j-\bar{x}_l)(\bar{w}_j-\bar{x}_l)} ((\bar{w}_j - \bar{x}_j) - (w_j - x_j)). \end{aligned}$$

If we write $\bar{p}^l_s = p^l_s + (\bar{p}^l_s - p^l_s)$, then the first term becomes the sum of $-(I^l_s - p^l_s) \frac{2\bar{x}_l^2(\bar{w}_j - \bar{x}_j)}{(\bar{x}_j-\bar{x}_l)(\bar{w}_j-\bar{x}_l)}$ and something bounded by $A \delta _{\textrm{total}}(s)$. The final term is also bounded by a multiple of $u_s$, and so for two bounded functions $A^l_1$ and $A^l_2$ we can write the *l*th summand as

5.24 $$\begin{aligned} -(I^l_s - p^l_s)\frac{2\bar{x}_l^2(\bar{w}_j - \bar{x}_j)}{(\bar{x}_j-\bar{x}_l)(\bar{w}_j-\bar{x}_l)} + A_1^l(s) \delta _{\textrm{total}}(s) + A_2^l(s) u_s. \end{aligned}$$

Similarly we can write the last line of (5.23) as

$$\begin{aligned} -(I^j_s - p^j_s)\left( 3 \bar{x}_j + \frac{2 \bar{x}_j^2}{\bar{w}_j - \bar{x}_j} \right) + (\bar{p}^j_s - p^j_s)\left( 3 \bar{x}_j + \frac{2 \bar{x}_j^2}{\bar{w}_j - \bar{x}_j} \right) \\ + 3 I^j_s \left( \bar{x}_j - x_j \right) + I^j_s \left( \frac{2\bar{x}_j^2}{\bar{w}_j - \bar{x}_j} - \frac{2x_j^2}{w_j-x_j} \right) . \end{aligned}$$

Linearising the final term on the line above by writing $\bar{x}_j^2 = \bar{x}_j x_j + \bar{x}_j(\bar{x}_j - x_j)$, and $x_j^2 = x_j \bar{x}_j + x_j(x_j - \bar{x}_j)$, we find that it is equal to

$$ \frac{2 I^j_s x_j \bar{x}_j}{(w_j-x_j)(\bar{w}_j-\bar{x}_j)} u_s + I^j_s\left( \frac{2\bar{x}_j}{\bar{w}_j - \bar{x}_j} + \frac{2 x_j}{w_j - x_j} \right) (\bar{x}_j - x_j). $$

On $H_j(n)$ we have $|w_j(s) - x_j(s)| \ge A^{-1} \sqrt{t-s}$ for all s<t and $|\bar{w}_j(s) - \bar{x}_j(s)| \ge A^{-1}\sqrt{t-s}$, so we can bound all the terms involving $|\bar{p}^j_s - p^j_s|$ and $|\bar{x}_j - x_j|$ by a constant multiple of $\frac{\delta _{\textrm{total}}(s)}{\sqrt{t-s}}$. Therefore the last line of (5.23) can be rewritten

5.25 $$\begin{aligned} -(I^j_s - p^j_s)\left( 3 \bar{x}_j + \frac{2 \bar{x}_j^2}{\bar{w}_j - \bar{x}_j} \right) + \frac{2 I^j_s x_j \bar{x}_j}{(w_j-x_j)(\bar{w}_j-\bar{x}_j)} u_s + A^j(s) \frac{\delta _{\textrm{total}}(s)}{\sqrt{t-s}} \end{aligned}$$

for a bounded function $A^j$. Substituting (5.24) and (5.25) back into (5.23), and noting that the $A^l_1$ terms can be absorbed by the $A^j$ term, we obtain

5.26 $$\begin{aligned} \dot{u}_s&= \left( \frac{2 I^j_s x_j \bar{x}_j}{(w_j-x_j)(\bar{w}_j-\bar{x}_j)} + \sum _{l\not =j}A^l_2(s) - 1 \right) u_s + A^j(s) \frac{\delta _{\textrm{total}}(s)}{\sqrt{t-s}}\nonumber \\&\phantom {=} -\sum _{l\not =j} \left( (I^l_s - p^l_s) \frac{2\bar{x}_l^2(\bar{w}_j - \bar{x}_j)}{(\bar{x}_j-\bar{x}_l)(\bar{w}_j-\bar{x}_l)} \right) - (I^j_s - p^j_s) \left( 3 \bar{x}_j + \frac{2 \bar{x}_j^2}{\bar{w}_j - \bar{x}_j}\right) . \end{aligned}$$

Let

$$\gamma (s) := \frac{2 I^j_s x_j \bar{x}_j}{(w_j-x_j)(\bar{w}_j-\bar{x}_j)} + \sum _{l\not =j}A^l_2(s) - 1$$

and let α(s) denote the remaining terms in (5.26),

$$\alpha (s) := A^j(s)\frac{\delta _{\textrm{total}}(s)}{\sqrt{t-s}} + \sum _{l=1}^k C^l_s (I^l_s - p^l_s),$$

where $C^j_s:= - (3\bar{x}_j + \frac{2\bar{x}_j^2}{\bar{w}_j - \bar{x}_j})$ and $C^l_s := -\frac{2\bar{x}_l^2 (\bar{w}_j - \bar{x}_j)}{(\bar{x}_j - \bar{x}_l)(\bar{w}_j - \bar{x}_l)}$ for l≠j.

The ODE becomes $\dot{u}_s = \gamma (s) u_s + \alpha (s)$. Let

$$\begin{aligned} Y(s) := \int _s^t \alpha (r)\,\textrm{d}r, \end{aligned}$$

then we can write *u* using an integrating factor as

$$\begin{aligned} u_s = -Y(s) + \int _s^t Y(r)\gamma (r)\exp \left( -\int _s^r \gamma (v)\,\textrm{d}v\right) \!\textrm{d}r, \end{aligned}$$

and so by the triangle inequality

5.27 $$\begin{aligned} |u_s| \le |Y(s)| + \int _s^t |Y(r)||\gamma (r)|\exp \left( -\int _s^r \textrm{Re}\gamma (v)\,\textrm{d}v\right) \!\textrm{d}r. \end{aligned}$$

Note that since $\arg (w_j(s) - x_j(s)) - \arg x_j(s) \rightarrow 0$ and $\arg ( \bar{w}_j(s) - \bar{x}_j(s) ) - \arg \bar{x}_j(s) \rightarrow 0$ as s↑t, the real part of γ(s) is bounded below, and so (5.27) gives us a simpler bound

$$\begin{aligned} |u_s| \le |Y(s)| + A\int _s^t |Y(r)||\gamma (r)|\textrm{d}r. \end{aligned}$$

Conditional on $H_j(n)$ we almost surely have $|\gamma (s)| \le A/(t-s)$ for all s∈[0,t), so |Y(s)|≤X(s) for the *X* defined in Lemma 5.10, and the result follows. □

#### Proof of Corollary 5.11

Let $M_s^l:= \int _s^t C_r^l (I^l_r - p^l_s)\,\textrm{d}s$. All *k* terms $|M_s^l|$ can be bounded in exactly the same way, so write $M_s = M^j_s$ and assume that $X(s) = A\int _s^t \frac{\delta _{\textrm{total}}(r)}{\sqrt{t-r}}\,\textrm{d}r + |M(s)|$. Let $G(s):= \frac{\delta _{\textrm{total}}(s)}{\sqrt{t-s}}$. We have the bounds

5.28 $$\begin{aligned} |u_0|&\le |X(0)| + A\int _0^t \frac{|X(s)|}{t-s}\textrm{d}s\nonumber \\&\le A\underbrace{\int _0^t |G(s)|\,\textrm{d}s}_{(A1)} + \underbrace{|M_0|}_{(A2)} + A\underbrace{\int _0^t \frac{1}{t-s}\left( \int _s^t |G(r)|\,\textrm{d}r\right) \textrm{d}s}_{(A3)} + A\underbrace{\int _0^t \frac{\left| M_s \right| }{t-s}\textrm{d}s}_{(A4)}, \end{aligned}$$

and

5.29 $$\begin{aligned} \int _0^t \frac{|u_s|}{t-s}\,\textrm{d}s&\le \int _0^t \frac{|X(s)|}{t-s}\,\textrm{d}s + A \int _0^t \frac{1}{t-s}\int _s^t \frac{|X(r)|}{t-r}\,\textrm{d}r \textrm{d}s\nonumber \\&\le \int _0^t \frac{|X(s)|}{t-s}\,\textrm{d}s \nonumber \\&\phantom {= }+ A \underbrace{\int _0^t \frac{1}{t-s} \int _{s}^t \frac{1}{t-r} \int _r^t |G(v)|\,\textrm{d}v \, \textrm{d}r \, \textrm{d}s}_{(B1)} + A \underbrace{\int _0^t \frac{1}{t-s} \int _s^t \frac{|M_r|}{t-r}\,\textrm{d}r \,\textrm{d}s}_{(B2)}. \end{aligned}$$

We will first bound the terms involving *G*: (A1), (A3) and (B1). Almost by definition, (A1) is bounded by $A \int _0^t \frac{\delta _{\textrm{total}}(s)}{\sqrt{t-s}}\,\textrm{d}s \le A \int _0^t \frac{\delta _{\textrm{total}}(s)}{(t-s)^{51/100}}\,\textrm{d}s$.

By Tonelli’s theorem, (A3) in (5.28) can be written

$$\begin{aligned} \int _0^t \int _s^t \frac{|G(r)|}{t-s}\,\textrm{d}r \textrm{d}s&= \int _0^t \int _0^r \frac{|G(r)|}{t-s}\,\textrm{d}s \textrm{d}r\\&= \int _0^t |G(r)| \log \frac{t}{t-r}\,\textrm{d}v\\&\le A \int _0^t \delta _{\textrm{total}}(r) \frac{\log \frac{t}{t-r}}{\sqrt{t-r}}\,\textrm{d}r\\&\le A \int _0^t \frac{\delta _{\textrm{total}}(r)}{(t-r)^{51/100}}\textrm{d}r, \end{aligned}$$

where the last inequality holds, modifying the constant *A*, because $\log \frac{t}{t-r} \le 100 \left( \frac{t}{t-r}\right) ^{1/100}$ for all r∈(0,t).

We can also bound (B1) in (5.29) by repeated applications of Tonelli’s theorem:

$$\begin{aligned}&\int _0^t \frac{1}{t-s} \int _{s}^{t} \frac{1}{t-r} \int _{r}^t |G(v)|\,\textrm{d}v\, \textrm{d}r\, \textrm{d}s\\&\quad \le A\int _{s=0}^t \frac{1}{t-s} \int _{r=s}^{t} \frac{1}{t-r} \int _{v=r}^t \frac{\delta _{\textrm{total}}(v)}{\sqrt{t-v}}\,\textrm{d}v\, \textrm{d}r\, \textrm{d}s\\&\quad = A \int _{v=0}^{t} \int _{r=0}^{v} \frac{\delta _{\textrm{total}}(v)}{\sqrt{t-v}}\frac{1}{t-r} \int _{s=0}^{r} \frac{1}{t-s}\,\textrm{d}s \, \textrm{d}r \, \textrm{d}v\\&\quad = A \int _{v=0}^{t} \int _{r=0}^{v} \frac{\delta _{\textrm{total}}(v)}{\sqrt{t-v}}\frac{1}{t-r}\log \frac{t}{t-r}\,\textrm{d}r \,\textrm{d}v\\&\quad \le A \int _{v=0}^t \int _{r=0}^v \frac{\delta _{\textrm{total}}(v)}{\sqrt{t-v}} \frac{t^{1/100}}{(t-r)^{1 + \frac{1}{100}}} \,\textrm{d}r \,\textrm{d}v\\&\quad \le A \int _0^t \frac{\delta _{\textrm{total}}(v)}{(t-v)^{51/100}}\,\textrm{d}v. \end{aligned}$$

Now we will bound the terms in (5.28) and (5.29) involving $M_s$: (A2), (A4) and (B2). First write $I^j_r - p^j_r = I^j_{\textbf{c}\left\lfloor r/\textbf{c} \right\rfloor } - p^j_r = (I^j_{\textbf{c}\left\lfloor r/\textbf{c} \right\rfloor } - p^j_{\textbf{c}\left\lfloor r/\textbf{c} \right\rfloor }) + (p^j_{\textbf{c}\left\lfloor r/\textbf{c} \right\rfloor } - p^j_r)$. Let

$$\begin{aligned} M_s' := \int _s^t (I^j_{\textbf{c}\left\lfloor r/\textbf{c} \right\rfloor } - p^j_{\textbf{c}\left\lfloor r/\textbf{c} \right\rfloor }) C_r\,\textrm{d}r, \end{aligned}$$

then by Proposition 5.4 we have $|p^j_{\textbf{c}\left\lfloor r/\textbf{c} \right\rfloor } - p^j_r| \le A\textbf{c}$ for all r∈[0,T], so

$$\begin{aligned} |M_s - M_s'| = \left| \int _s^t (p^j_{\textbf{c}\left\lfloor r/\textbf{c} \right\rfloor } - p^j_r) C_r \,\textrm{d}r \right|&\le \int _s^t |p^j_{\textbf{c}\left\lfloor r/\textbf{c} \right\rfloor } - p^j_r| |C_r| \,\textrm{d}r\\&\le A \int _s^t \textbf{c}(t-r)^{-1/2}\,\textrm{d}r \le A \textbf{c}\sqrt{t-s}. \end{aligned}$$

Therefore in all of (A2), (A4) and (B2) we can replace *M* with $M^{\prime }$ by adding an extra error term bounded by $$A\sqrt{t}\,\textbf{c}$$. Recall that $$t = n\textbf{c}$$. If $$t-s < 10\textbf{c}$$ then we have the deterministic bound $$|M'_s| \le A\int _{0}^{t-s}r^{-1/2}\,\textrm{d}r \le A \sqrt{t-s}$$. If $$t-s \ge 10\textbf{c}$$, it is easy to bound the second moment of $$|M'_s|$$ since $$(I^j_{m\textbf{c}} - p^j_{m\textbf{c}})_{m \ge 1}$$ are martingale increments:

$$\begin{aligned} \mathbb {E}( |M_s'|^2 \,|\, H_j(n) ) \le A\mathbb {E}( |M_s'|^2 )&= A\sum _{m=\left\lfloor s/\textbf{c} \right\rfloor }^{n-1} \mathbb {E}( (I^j_{m\textbf{c}} - p^j_{m\textbf{c}})^2 ) \left( \int _{m\textbf{c}\vee s}^{(m+1)\textbf{c}} |C_r| \,\textrm{d}r\right) ^2\\&\le A \sum _{m=\left\lfloor s/\textbf{c} \right\rfloor }^{n-1} \left( \int _{m\textbf{c}\vee s}^{(m+1)\textbf{c}} (n\textbf{c}-r)^{-1/2} \,\textrm{d}r \right) ^2\\&= A \sum _{m=0}^{n - \left\lfloor s/\textbf{c} \right\rfloor -1} \left( \int _{m\textbf{c}}^{(m+1)\textbf{c}\wedge (t-s)} r^{-1/2}\,\textrm{d}r \right) ^2\\&\le A\textbf{c}\log \left( \frac{t-s}{\textbf{c}}\right) \le A\textbf{c}\log (1/\textbf{c}). \end{aligned}$$

For $s \in [0,t-10\textbf{c}]$, define the event

$$\begin{aligned} E_s := \{ |M_s'| \le \textbf{c}^{\frac{1}{2} - \frac{4}{R}} \}, \end{aligned}$$

then by Markov’s inequality

$$ 1 - \mathbb {P}(E_s | H_j(n) ) \le \mathbb {P}( |M_s'|^2 > \textbf{c}^{1 - \frac{8}{R}} | H_j(n) ) \le \frac{A\textbf{c}\log (1/\textbf{c})}{\textbf{c}^{1 - \frac{8}{R}}} \le A \textbf{c}^{\frac{8}{R}} \log (1/\textbf{c}). $$

We will choose a particular sequence $(s_m)_{1 \le m \le N_t}$ in $[0,t-10\textbf{c}]$ such that $\bigcap _{m=1}^{N_t} E_{s_m}$ holds with high probability, and on this event $|M_s'|$ is bounded uniformly on $[0,t-10\textbf{c}]$. Let $r_1 = 10\textbf{c}$ and $s_1 = t - r_1$. for m≥1 set $r_{m+1} = r_m + \textbf{c}^{\frac{1}{R}}\sqrt{r_m}$ and $s_m = \max (t-r_m,0)$. Then $r_{m+1} - r_m \ge \textbf{c}^{\frac{1}{R}} \sqrt{r_m - r_{m-1}}$, and so $r_{m+1} - r_m \ge \textbf{c}^{\frac{1}{2^{m}} + \frac{1}{R}(2 - \frac{1}{2^{m}})} \ge \textbf{c}^{\frac{4}{R}}$ for *m* greater than some constant $m_{R}$. Let $N_t:= \min \{m: r_m \ge t \}$ and it follows from the above that $N_t \le A\textbf{c}^{-\frac{4}{R}}$. Let $s \in (s_{m+1},s_m)$ for some m≥1, then

$$\begin{aligned} |M'_s - M'_{s_m}| \le \int _{s}^{s_m} |C_r|\,\textrm{d}r&\le (s_m - s_{m+1}) \sup _{r \in [s_{m+1},s_m]} |C_r|\\&\le A(r_{m+1} - r_m) r_m^{-1/2}\\&= A \textbf{c}^{1/R}. \end{aligned}$$

Therefore, on $\bigcap _{m=1}^{N_t} E_{s_m}$, $|M'_s| \le A\textbf{c}^{1/R}$ for all $s \in [0,t-10\textbf{c}]$, and by the union bound $\mathbb {P}(\bigcap _{m=1}^{N_t} E_{s_m} | H_j(n)) \ge 1 - N_t A \textbf{c}^{8/R}\log (1/\textbf{c}) \ge 1 - A \textbf{c}^{4/R} \log (1/\textbf{c})$.

On this event we can bound (A4) of (5.28),

$$\begin{aligned} \int _0^t \frac{|M_s'|}{t-s}\,\textrm{d}s&\le \int _0^{t-10\textbf{c}} \frac{\textbf{c}^{1/R}}{t-s}\,\textrm{d}s + \int _{t-10\textbf{c}}^t \frac{A\sqrt{t-s}}{t-s}\,\textrm{d}s\\&\le A\textbf{c}^{1/R}\log (1/\textbf{c}) + A \textbf{c}^{1/2}, \end{aligned}$$

and similarly (B2) of (5.29) has a bound

$$\begin{aligned} \int _0^t \frac{1}{t-s} \int _{s}^{t} \frac{|M_r'|}{t-r}\,\textrm{d}r \,\textrm{d}s \le A \textbf{c}^{1/R}( \log (1/\textbf{c}))^2 + A \textbf{c}^{1/2}. \end{aligned}$$

We can then use these bounds in (5.28) and (5.29) to complete our proof. □

#### Proof of Proposition 5.6

The five results from Lemma 5.7 to Corollary 5.11 show that for a given $n \le \left\lfloor T/\textbf{c} \right\rfloor$, the bound $\delta _\textrm{p}(t) \le A \int _0^t \frac{\delta _{\textrm{total}}(s)}{(t-s)^{51/100}}\textrm{d}s + A \textbf{c}^{1/R}(\log (1/\textbf{c}))^2$ holds with high (conditional) probability if $H_j(n)$ occurs. Let *B*(*t*) be the event that this bound holds at time *t*. We will derive the claimed global bound from the fact that $\mathbb {P}(B(n\textbf{c}) | H_j(n))$ is close to 1 for fixed *n*.

For simplicity assume that $\textbf{c}^{-1 + \frac{1}{2R}}$ and $T/\textbf{c}^{\frac{1}{2R}}$ are integers. For $i \in \{0, \dots , T\textbf{c}^{-\frac{1}{2R}}-1\}$ the probability that $H_j(n)$ does not hold for any $n \in \{ i{\textbf{c}^{-1+ \frac{1}{2R}}} + 1, i{\textbf{c}^{-1+ \frac{1}{2R}}} + 2, \dots , (i+1){\textbf{c}^{-1+ \frac{1}{2R}}} \}$ is bounded by $(1-p)^{\textbf{c}^{-1 + \frac{1}{2R}}}$ for a positive constant *p*, and so the expected number of such *i* is less than $T\textbf{c}^{-\frac{1}{2R}}(1-p)^{\textbf{c}^{-1+\frac{1}{2R}}} \ll 1$. Therefore by Markov’s inequality there exists, with very high probability, a sequence of times $\tau _0< \tau _1< \dots < \tau _{T\textbf{c}^{-\frac{1}{2R}}-1}$ such that for each *i*, $\tau _i \in (i\textbf{c}^{\frac{1}{2R}},(i+1)\textbf{c}^{\frac{1}{2R}}]$, $\tau _i/\textbf{c}$ is an integer, $H_j(\tau _i/\textbf{c})$ holds, and $\tau _{i+1} - \tau _i \le 2 \textbf{c}^{\frac{1}{2R}}$. Since $\mathbb {P}(B(n\textbf{c}) | H_j(n)) \ge 1 - A \textbf{c}^{4/R}\log (1/\textbf{c})$ for a fixed *n*, the conditional probability of $\bigcap _{i=0}^{T\textbf{c}^{-\frac{1}{2R}}-1}B(\tau _i)$ given $(\tau _i)_{0 \le i \le T\textbf{c}^{-\frac{1}{2R}}-1}$ is at least $1 - A \textbf{c}^{\frac{7}{2R}}\log (1/\textbf{c})$.

Therefore with high probability we have a sequence of times $(\tau _i)_{0 \le i \le T\textbf{c}^{-\frac{1}{2R}}-1}$ as above such that $B(\tau _i)$ holds for each *i*.

Let $t \in (\tau _i, \tau _{i+1})$ for some $i \le T\textbf{c}^{-\frac{1}{2R}}-1$. Then by Proposition 5.4,

$$\begin{aligned} \delta _{\textrm{p}}(t)&= \delta _{\textrm{p}}(\tau _i) + (\delta _{\textrm{p}}(t) - \delta _{\textrm{p}}(\tau _i))\\&\le \delta _{\textrm{p}}(\tau _i) + A ( \tau _{i+1} - \tau _i )\\&\le \delta _{\textrm{p}}(\tau _i) + A \textbf{c}^{\frac{1}{2R}}\\&\le A \int _0^{\tau _i}\frac{\delta _{\textrm{total}}(s)}{(\tau _i-s)^{51/100}}\textrm{d}s + A \textbf{c}^{\frac{1}{2R}}, \end{aligned}$$

where the extra $A \textbf{c}^{1/R}(\log (1/\textbf{c}))^2$ term is absorbed by the $A\textbf{c}^{1/2R}$. Then since $t > \tau _i$, we have $(\tau _i - s)^{-51/100} < (t - s)^{-51/100}$ for all $s \in (0,\tau _i)$, and so

$$ \delta _{\textrm{p}}(t) \le A \int _0^t \frac{\delta _{\textrm{total}}(s)}{(t-s)^{51/100}}\textrm{d}s + A \textbf{c}^{\frac{1}{2R}} $$

for all t∈[0,T], as claimed. □

#### Proof of Theorem 5.2

Propositions 5.5 and 5.6 give us $\delta _{\textrm{total}}(t) \le A \int _0^t \frac{\delta _{\textrm{total}}(s)}{(t-s)^{51/100}}\,\textrm{d}s + A\textbf{c}^{1/2R}$ for all t∈[0,T]. Using a singular version of Grönwall’s inequality (see [16, Corollary 2]), this implies $\delta _{\textrm{total}}(T) \le A \textbf{c}^{1/2R}$ as required. □

## Proof of main theorem

We did most of the hard work in Section 5, and will now bring together the intermediate convergence results to show convergence of the ALE to the LPM.

### Proof of Theorem 2.1

Let $\xi _t$ be the driving function of the ALE, and $\bar{\mu }_t$ the driving measure of the LPM. To show that the two converge in distribution, we will show that $d_{\textrm{BW}}(\delta _{\xi _t} \otimes m_{[0,T]}, \bar{\mu }_t \otimes m_{[0,T]}) \rightarrow 0$ in probability as $\textbf{c}\rightarrow 0$.

Note that another way of writing (4.1) for the auxiliary process is

$$\begin{aligned} \Phi _n^* = R_{\delta _1 + \dots + \delta _n} \circ \left( \Phi _0 \circ f_{\theta _1^*-\delta _1} \circ f_{\theta ^*_2 - (\delta _1 + \delta _2)} \circ \dots \circ f_{\theta ^*_n - (\delta _1 + \dots + \delta _n)} \right) \circ R_{-(\delta _1 + \dots +\delta _n)} \end{aligned}$$

So let $\xi _t^*$ be the driving measure for the angle sequence $(\theta ^*_n - (\delta _1 + \dots + \delta _n) )_{n \le \left\lfloor T/\textbf{c} \right\rfloor }$. By Theorem 4.1 we have a coupling between $\xi ^*$ and ξ, such that if we define the event $E_1 = \{ \tau _D \wedge \tau _{\textrm{coupling}}> \left\lfloor T/\textbf{c} \right\rfloor \}$, then $\mathbb {P}(E_1) \ge 1 - A\textbf{c}^{1/2}$. On $E_1$ note that

$$\begin{aligned} \sup _{t \in [0,T]} |\xi _t - \xi ^*_t| \le \left( \frac{T}{\textbf{c}} + 2\right) D. \end{aligned}$$

Next, to pass from the auxiliary model to the multinomial model, let $\xi ^{\textrm{multi}}_t$ be the driving measure of the multinomial model, define the event $E_2 = \{ \tau _{\not =} > \left\lfloor T/\textbf{c} \right\rfloor \}$, and note that on $E_2$ the models coincide: $\xi ^{\textrm{multi}}_t = \xi ^*_t$ for all t∈[0,T]. By Corollary 4.8, $\mathbb {P}(E_2) \ge 1 - AT\textbf{c}^{-2}D$.

Finally, by Corollary 5.3,

$$\begin{aligned} d_{\textrm{BW}}(\delta _{\xi ^{\textrm{multi}}_t} \otimes m_{[0,T]}, \bar{\mu }_t \otimes m_{[0,T]}) \rightarrow 0 \end{aligned}$$

in probability as $\textbf{c}\rightarrow 0$.

Then by the triangle inequality,

$$\begin{aligned} d_{\textrm{BW}}(\delta _{\xi _t} \otimes m_{[0,T]}, \bar{\mu }_t \otimes m_{[0,T]})&\le d_{\textrm{BW}}(\delta _{\xi _t} \otimes m_{[0,T]}, \delta _{\xi ^*_t} \otimes m_{[0,T]})\\&\phantom {\le }+ d_{\textrm{BW}}(\delta _{\xi ^*_t} \otimes m_{[0,T]}, \bar{\mu }_t \otimes m_{[0,T]}), \end{aligned}$$

and on $E_1 \cap E_2$ this is bounded by

$$\begin{aligned} T \left( \frac{T}{\textbf{c}} + 2 \right) \! D + d_{\textrm{BW}}(\delta _{\xi ^{\textrm{multi}}_t} \otimes m_{[0,T]}, \bar{\mu }_t \otimes m_{[0,T]}). \end{aligned}$$

Since $\textbf{c}^{-1}D = o(1)$, this upper bound tends to zero in probability as $\textbf{c}\rightarrow 0$, and $\mathbb {P}( E_1 \cap E_2 ) \rightarrow 1$, giving us $d_{\textrm{BW}}(\delta _{\xi _t} \otimes m_{[0,T]}, \bar{\mu }_t \otimes m_{[0,T]}) \overset{p}{\rightarrow }\ 0$ as required. □

## Data Availability

No datasets were generated or analysed during the current study.

## Footnotes

1 This follows from the fact that $c(K) = \int _0^1 \log |f_K(e^{2\pi i t})|\,\textrm{d}t$.

2 A function $f: [0,T] \rightarrow \mathbb {R}$ is said to be càdlàg (right-continuous with left limits) if, for every x∈[0,T] and every decreasing sequence $x_n \downarrow x$ and increasing sequence $y_n \uparrow x$, $f(x_n) \rightarrow f(x)$ as $n \rightarrow \infty$ and $\lim _{n\rightarrow \infty }f(y_n)$ exists (but is not necessarily equal to *f*(*x*)).

3 https://www.youtube.com/playlist?list=PLiaV5rk6Gk7pCjwGQaVOZ1edfbU3xK6td

4 The backward equation appears in [13] in the proof of Theorem 4.13.
